# The disease resistance protein SNC1 represses the biogenesis of microRNAs and phased siRNAs

**DOI:** 10.1038/s41467-018-07516-z

**Published:** 2018-11-29

**Authors:** Qiang Cai, Chao Liang, Suikang Wang, Yingnan Hou, Lei Gao, Li Liu, Wenrong He, Wenbo Ma, Beixin Mo, Xuemei Chen

**Affiliations:** 1 Guangdong Provincial Key Laboratory for Plant Epigenetics, College of Life Sciences and Oceanography, Shenzhen University, Shenzhen, 518060 China; 2 Key Laboratory of Optoelectronic Devices and Systems of Ministry of Education and Guangdong Province, College of Optoelectronic Engineering, Shenzhen University, Shenzhen, 518060 China; 3 Department of Botany and Plant Sciences, Institute of Integrative Genome Biology, University of California, Riverside, CA 92521 USA; 4 Department of Microbiology and Plant Pathology, University of California, Riverside, CA 92521 USA

## Abstract

Plants evolved an array of disease resistance genes (*R* genes) to fight pathogens. In the absence of pathogen infection, *NBS-LRR* genes, which comprise a major subfamily of *R* genes, are suppressed by a small RNA cascade involving microRNAs (miRNAs) that trigger the biogenesis of phased siRNAs (phasiRNAs) from *R* gene transcripts. However, whether or how *R* genes influence small RNA biogenesis is unknown. In this study, we isolate a mutant with global defects in the biogenesis of miRNAs and phasiRNAs in *Arabidopsis thaliana* and trace the defects to the over accumulation and nuclear localization of an R protein SNC1. We show that nuclear SNC1 represses the transcription of miRNA and phasiRNA loci, probably through the transcriptional corepressor TPR1. Intriguingly, nuclear SNC1 reduces the accumulation of phasiRNAs from three source *R* genes and concomitantly, the expression of a majority of the ~170*R* genes is up-regulated. Taken together, this study suggests an *R* gene-miRNA-phasiRNA regulatory module that amplifies plant immune responses.

## Abstract (web summary)

A small RNA-based signaling cascade prevents the induction of plant resistance genes (*R*-genes) in the absence of pathogen challenge. Here Cai *et al*. show that nuclear accumulation of the R protein SNC1 can activate immunity by suppressing small RNA production and releasing *R*-gene repression.

## Introduction

Plants often suffer from attacks by various pathogens and have evolved a series of defense mechanisms. One important mechanism is *Resistance* gene (*R* gene)-mediated disease resistance^1^. R proteins are activated upon pathogen infection and cause local programmed cell death named hypersensitive response (HR)^2^. Local HR often leads to systemic and broad-spectrum resistance to pathogens called systemically acquired resistance^3,4^.

*R* genes are classified into at least five groups according to domain composition^5^. Among them, the *NBS-LRR* family containing a nucleotide binding site (NBS) and a C-terminal leucine rich repeat domain (LRR) is the largest. *NBS-LRR* genes are widespread in plant genomes, usually numbering in hundreds^6,7^. The genome of *Arabidopsis thaliana* contains approximately 170 *NBS-LRR* genes^8^.

While *R* genes confer resistance to pathogens, their constitutive expression is detrimental to plant growth such that their expression must be tightly regulated. Some *NBS-LRR* genes are silenced by microRNAs (miRNAs)^9,10^; a notable example is the tobacco *N* gene that confers resistance toward tobacco mosaic virus (TMV)^11^. But repression of *R* gene expression by small RNAs is not limited to individual miRNA–*R* gene interactions and entails global effects mediated by phased siRNAs (phasiRNAs). PhasiRNAs are a class of small RNAs that is widespread in the plant kingdom. PhasiRNA biogenesis is triggered by miRNA-guided cleavage of noncoding transcripts or certain protein coding transcripts. A cleavage fragment from these transcripts becomes the source of phasiRNAs^12^. In *Arabidopsis*, eight noncoding *TAS* loci generate phasiRNAs, and these small RNAs were originally named trans-acting small interfering RNAs (tasiRNAs) as they regulate targets *in trans*^13–16^. Emerging evidence indicates that plants utilize specific miRNAs to target some *NBS-LRR* genes and trigger the production of phasiRNAs from these transcripts. The phasiRNAs are thought to regulate *NBS-LRR* genes either in *cis* or in *trans*, forming a self-reinforcing regulatory network that globally suppresses *R* gene expression^9,17^. In *Arabidopsis*, miR472 and miR825^*^ target two *NBS-LRR* genes and trigger the production of phasiRNAs from these transcripts^18–20^.

Given that *R* genes are globally repressed by a miRNA–phasiRNA cascade, how do plants overcome this repression to induce and maintain *R* gene expression during defense? Do *R* genes also influence small RNA biogenesis? In a genetic screen designed to uncover players of miRNA biogenesis, we isolated a mutant with global defects in miRNA biogenesis. Rather unexpectedly, the defects in miRNA biogenesis in this mutant were traced to the over accumulation and nuclear localization of the *R* gene product SNC1. We showed that nuclear SNC1 likely inhibited miRNA biogenesis by repressing the transcription of *MIR* genes together with its interacting protein TPR1, a transcriptional co-repressor. SNC1 and TPR1 also repressed the accumulation of phasiRNAs from three *R* genes and led to a global induction of the *R* family. This work revealed a mechanism whereby an activated R protein may relieve the global repression of *R* genes by small RNAs to enable defense. While the previously known miRNA–phasiRNA–*R* genes module represses *R* gene expression in the absence of pathogens, the *R* gene–miRNA–phasiRNA module discovered in this study likely enables *R* gene expression during defense.

## Results

### Isolation of a mutant with reduced abundance of miRNAs

**Fig. 1:**
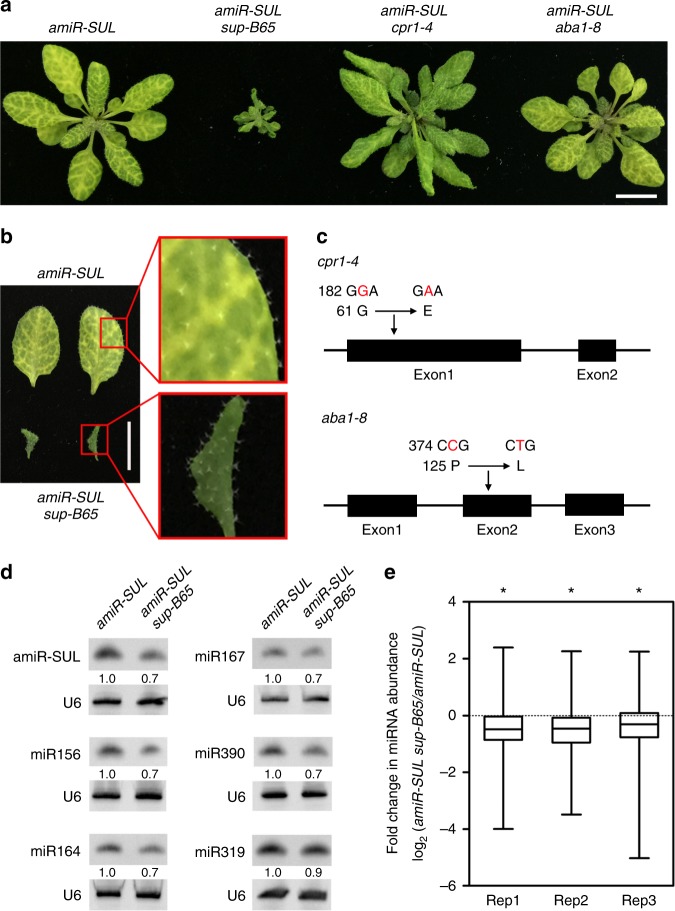
Isolation and characterization of a mutant with reduced accumulation of miRNAs. **a** Plants of the following genotypes: *amiR-SUL*, *amiR-SUL sup-B65*, *amiR-SUL cpr1–4*, and *amiR-SUL aba1–8*. In addition to reduced leaf bleaching, *amiR-SUL sup-B65* plants exhibit pleiotropic phenotypes, including dwarfism and twisted leaves. Bar = 1 cm. **b** Rosette leaves from *amiR-SUL* and *amiR-SUL sup-B65* plants. Portions of the leaves (as marked by the rectangles) were magnified to show the phenotypes of vein-centered bleaching. Bar = 1 cm. **c** Two candidate mutated sites were identified in *amiR-SUL sup-B65*, one being a transition of G to A, which results in the substitution of Gly182 for Glu, in *CPR1* (At4g12560) and the other being a transition of C to T, which leads to the substitution of Pro125 for Leu, in *ABA1* (At5g63070). The two alleles were named *cpr1–4* and *aba1–8*. **d** Northern blots to detect six miRNAs (amiR-SUL, miR156, miR164, miR167, miR319, and miR390) in 15-day-old seedlings of *amiR-SUL* and *amiR-SUL sup-B65*. U6 served as an internal control. The numbers indicate the relative abundance of the miRNAs in the two genotypes. **e** Small RNA sequencing from 15-day-old seedlings of *amiR-SUL* and *amiR-SUL sup-B65* in three biological replicates (Rep1, Rep2 and Rep3). A global reduction in miRNA accumulation was observed in *amiR-SUL sup-B65* relative to *amiR-SUL* (^*^Student’s *t* test: *P* < 0.05). Source data are provided as a Source Data file

An ethylmethane sulfonate (EMS) mutagenesis screen was performed with the *Arabidopsis* line *pSUC2:amiR-SUL*^21^, which will be referred to as *amiR-SUL* thereafter, to identify genes required for miRNA biogenesis and/or activity. In this *amiR-SUL* line, an artificial miRNA is expressed in the vasculature and silences the endogenous chlorophyll biosynthesis gene *CHLORINA42* (also known as *SULFUR, SUL*) in mesophyll cells to result in vein-centered leaf bleaching. We isolated a suppressor mutant (*amiR-SUL sup-B65*) with reduced vein-centered bleaching, a phenotype implying compromised amiR-SUL activity (Fig. 1a, b). In addition to reduced leaf bleaching, *amiR-SUL sup-B65* showed pleiotropic phenotypes, including dwarfism and twisted leaves (Fig. 1a, b). The *amiR-SUL sup-B65* mutant was backcrossed with the parental *amiR-SUL* line, and plants resembling *amiR-SUL sup-B65* in morphology (reduced plant size and reduced leaf bleaching) constituted 1/16 of the F2 population (20 out of 337 F2 plants); this segregation ratio is consistent with the phenotype being caused by two unlinked, recessive mutations (*χ*^2^ = 0.05; *P* = 0.822; Table 1). The *amiR-SUL sup-B65* line was also crossed with wild type (Col) to remove the *amiR-SUL* transgene. Plants with morphology (reduced plant size) similar to *amiR-SUL sup-B65* but without the *amiR-SUL* transgene were recovered and will be referred to as *sup-B65* (Supplementary Fig. 1a).

**Table 1:** Segregation analysis in the F2 population

F2 backcross (female × male)	Number of plants with *amiR-SUL sup-B65* phenotype	Number of total F2 plants	Plants with *amiR-SUL sup-B65* phenotype/total (expected)	*χ*^2^ (*P* value) for 1:16
*amiR-SUL* × *amiR-SUL sup-B65*	20	337	5.93% (6.25%)	0.05 (0.822)

**Fig. 2:**
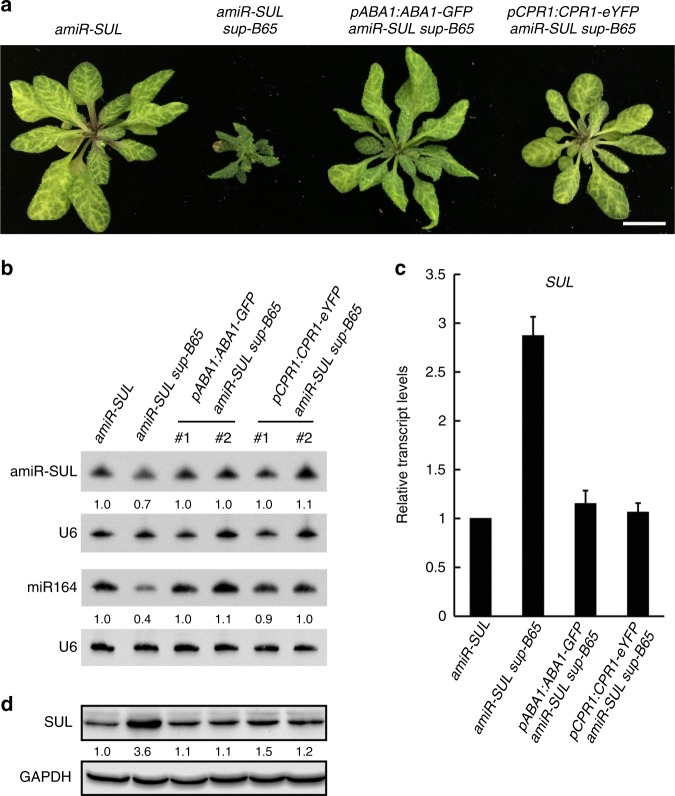
Simultaneous mutations in *CPR1* and *ABA1* cause the phenotypes of *amiR-SUL sup-B65*. **a** Plants of the indicated genotypes. *amiR-SUL sup-B65* plants were transformed with *pCPR1:CPR1-eYFP* or *pABA1:ABA1-GFP*. Either transgene fully rescued the morphological defects of *amiR-SUL sup-B65*. Bar = 1 cm. **b** Northern blots to determine the abundance of amiR-SUL and an endogenous miRNA in plants of the indicated genotypes. Two independent transgenic lines of *pCPR1:CPR1-eYFP* and *pABA1:ABA1-GFP* were used. U6 served as the internal control. The numbers indicate relative abundance of the miRNAs. **c** Relative transcript levels of the amiR-SUL target gene *SUL* in the indicated genotypes as determined by quantitative RT-PCR. The levels of *SUL* transcript in *amiR-SUL sup-B65* were restored to those in *amiR-SUL* by either the *pCPR1:CPR1-eYFP* or *pABA1:ABA1-GFP* transgene. Error bars represent standard deviations calculated from three biological replicates. **d** Western blots to determine SUL protein levels in plants of the same genotypes as in **b**. GAPDH served as a loading control. SUL protein levels were elevated in *amiR-SUL sup-B65* but restored by either *pCPR1:CPR1-eYFP* or *pABA1:ABA1-GFP* in two independent transgenic lines. Source data are provided as a Source Data file

The reduced leaf bleaching in *amiR-SUL sup-B65* could be caused by lower levels of amiR-SUL. Indeed, northern blotting showed that the levels of amiR-SUL were reduced to approximately 70% of wild type levels (Figs. 1d and 2b). The expression of *SUL* was anticorrelated with that of amiR-SUL—the levels of both *SUL* mRNA (Fig. 2c) and protein (Fig. 2d) were increased in *amiR-SUL sup-B65* as compared to *amiR-SUL*.

In order to investigate whether this mutant had a general defect in miRNA biogenesis, we performed northern blotting to examine endogenous miRNAs. The levels of all tested miRNAs (miR156, miR164, miR167, miR319, and miR390) were reduced by 10–30% in *amiR-SUL sup-B65* relative to *amiR-SUL* (Fig. 1d). To obtain a global perspective on miRNAs, we performed small RNA sequencing from 15-day-old seedlings of *amiR-SUL* and *amiR-SUL sup-B65* in three biological replicates. A global reduction in miRNA accumulation was observed in *amiR-SUL sup-B65* (Fig. 1e and Supplementary Data 1). Therefore, the *amiR-SUL sup-B65* mutant is partially defective in miRNA biogenesis.

### Mutations in *CPR1* and *ABA1* underlie the mutant phenotypes

Whole genome resequencing was performed to identify the mutations in *amiR-SUL sup-B65*. The *amiR-SUL sup-B65* mutant was backcrossed with the parental *amiR-SUL* line, and F2 plants exhibiting the phenotypes of *amiR-SUL sup-B65* (reduced plant size and reduced leaf bleaching) were pooled for resequencing. Two candidate mutated sites were identified, one being a transition of G to A, which results in the substitution of Gly182 for Glu, in *CPR1* (At4g12560) (Fig. 1c) and the other being a transition of C to T, which leads to the substitution of Pro125 for Leu, in *ABA1* (At5g63070) (Fig. 1c). These two mutations were named *cpr1–4* and *aba1–8*, respectively. We first tested the linkage of these two mutations with the *amiR-SUL sup-65* phenotype (dwarf morphology and reduced area of vein-centered bleaching). In the F2 progeny of the backcross with the parental *amiR-SUL* line, 72 plants with the *amiR-SUL sup-B65* phenotype were genotyped for *cpr1–4* and *aba1–8*. All 72 plants were homozygous for both mutations, indicating that the visible phenotype of *amiR-SUL sup-B65* was linked to *cpr1–4* and *aba1–8*. From the same F2 population, we also genotyped plants that do not have the *amiR-SUL sup-B65* phenotype and identified homozygous plants containing only the *cpr1–4* or the *aba1–8* mutation. The *aba1–8* mutation had no effect on the leaf bleaching phenotype, and the *cpr1–4* mutation had a weak effect, as reflected by the “greener” look of the *amiR-SUL cpr1–4* plants in comparison to *amiR-SUL* plants (Fig. 1a). Neither mutation by itself caused any drastic changes in plant size (Fig. 1a).

We identified *cpr1–4* and *aba1–8* single mutants as well as the *cpr1–4 aba1–8* double mutant without the *amiR-SUL* transgene in the F2 progeny of a cross between *amiR-SUL sup-B65* and Col (Supplementary Fig. 1a). The two single mutants were larger than the double mutant (Supplementary Fig. 1a). To confirm that the combination of mutations in *CPR1* and *ABA1* leads to a drastic reduction in plant size, we obtained two T-DNA insertion lines, SALK_045148 (*cpr1–3*)^22^ and SALK_059469 (which we named *aba1–7*), and generated the double mutant. The *cpr1–3 aba1–7* double mutant, but not either single mutant, had drastically reduced plant size resembling *cpr1–4 aba1–8* (Supplementary Fig. 1b).

We performed complementation tests to confirm that the reduced plant size in *cpr1–4 aba1–8* and the reduced leaf bleaching in *amiR-SUL sup-B65* were due to mutations in both *CPR1* and *ABA1*. Both *cpr1–4 aba1–8* and *amiR-SUL sup-B65* plants were transformed with *pCPR1:CPR1-eYFP* or *pABA1:ABA1-GFP*. Either transgene fully rescued the morphological defects of *amiR-SUL sup-B65* (Fig. 2a) and *cpr1–4 aba1–8* (Supplementary Fig. 1c), indicating that only the combination of *cpr1* and *aba1* mutations led to drastically reduced plant size. The reduced leaf bleaching in *amiR-SUL sup-B65* was also rescued by either transgene (Fig. 2a). Consistently, either transgene restored the levels of amiR-SUL (Fig. 2b), as well as the levels of *SUL* RNA (Fig. 2c) and SUL protein (Fig. 2d), to wild-type levels. Moreover, either transgene rescued the defect in miR164 accumulation in the *amiR-SUL sup-B65* mutant (Fig. 2b). Therefore, the miRNA biogenesis and plant size defects of *amiR-SUL sup-B65* were attributable to a combination of *cpr1–4* and *aba1–8*. For simplicity, we thereafter refer to the double mutant *cpr1–4 aba1–8* as *cpr1 aba1*.

### *MIR* promoter activities are reduced in *cpr1 aba1*

**Fig. 3:**
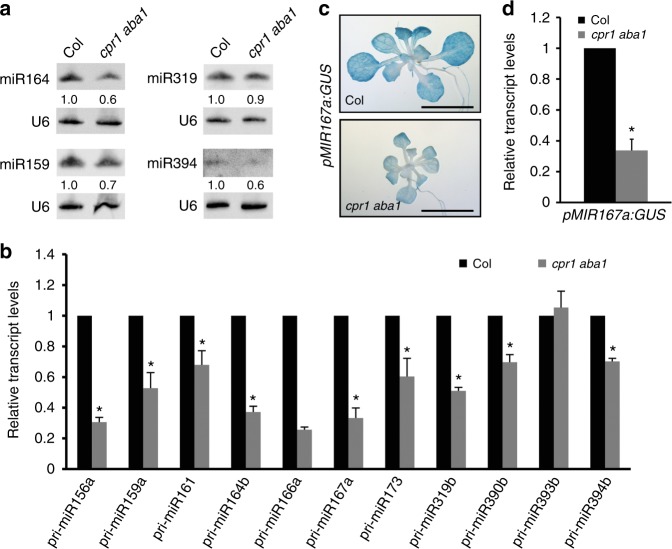
*MIR* promoter activities are reduced in *cpr1 aba1*. **a** Northern blots for four miRNAs in 15-day-old seedlings of wild type (Col) and *cpr1 aba1*. U6 served as the loading control. The numbers indicate relative abundance. **b** The levels of eleven pri-miRNAs in wild type (Col) and *cpr1 aba1* as determined by quantitative RT-PCR. Error bars represent standard deviation calculated from three biological replicates (^*^Student’s *t* test: *P* < 0.05). **c** GUS staining of *pMIR167a:GUS* and *pMIR167a:GUS cpr1 aba1* seedlings. Note that the *pMIR167a:GUS* transgene was at a single locus and was homozygous in the two lines. The staining was visibly lower in *pMIR167a:GUS cpr1 aba1*. Bar = 5 mm. **d** GUS transcript levels as determined by quantitative RT-PCR analysis of the genotypes in **c**. Error bars represent standard deviations calculated from three biological replicates (^*^Student’s *t* test: *P* < 0.05). Source data are provided as a Source Data file

We sought to determine how miRNA biogenesis is affected by mutations in *CPR1* and *ABA1*. We first tested whether the levels of endogenous miRNAs are affected by *cpr1* and *aba1* mutations when the *amiR-SUL* transgene is not present. We determined the levels of miR159, miR164, miR319, and miR394 in 15-day-old seedlings of *cpr1 aba1* and wild type by northern blotting. All of them showed a modest reduction in *cpr1 aba1* (Fig. 3a). Given that mature miRNAs are processed from pri-miRNAs (primary miRNAs), we next performed real-time RT-PCR to examine the levels of eleven pri-miRNAs in Col and *cpr1 aba1*. The levels of these pri-miRNAs were decreased in *cpr1 aba1* as compared to Col except for pri-miR393b (Fig. 3b). The reduced levels of pri-miRNAs may result from reduced transcription of *MIR* genes or enhanced degradation of pri-miRNAs. We tested whether the reduction in pri-miRNA levels is attributable to impaired *MIR* promoter activity. The *cpr1 aba1* double mutant was crossed with a transgenic line in which a *GUS* reporter gene driven by the *MIR167a* promoter (*pMIR167a:GUS*) is inserted into a single locus^23,24^. In the F3 generation, we obtained *cpr1 aba1* plants containing homozygous *pMIR167a:GUS*. GUS activity was visibly lower in *cpr1 aba1* than in Col as revealed by GUS staining (Fig. 3c). Real-time RT-PCR analysis confirmed that the *cpr1 aba1* double mutant had lower *GUS* transcript levels than wild type (Fig. 3d). Therefore, defects in miRNA biogenesis in *cpr1 aba1* are likely due to reduced transcription of *MIR* genes.

### Derepression of SNC1 suppresses miRNA biogenesis

*CPR1* encodes an F-Box protein that functions as a negative regulator of the R protein SNC1 likely through SCF (Skp1-cullin-F-box)-mediated protein degradation^22,25^. Loss of *CPR1* function leads to increased SNC1 protein levels and constitutive defense responses^25^. *ABA1* encodes a zeaxanthin epoxidase that functions in the first step of the biosynthesis of the abiotic stress hormone abscisic acid (ABA)^26^. While ABA is well known for its role in plants’ resistance to abiotic stresses^27^, a recent study revealed that ABA deficiency promotes the activity and nuclear localization of SNC1 and other R proteins such as RPS4^28^. Therefore, it is reasonable to speculate that *SNC1* underlies the *amiR-SUL* suppressor phenotype of *amiR-SUL cpr1 aba1*.

**Fig. 4:**
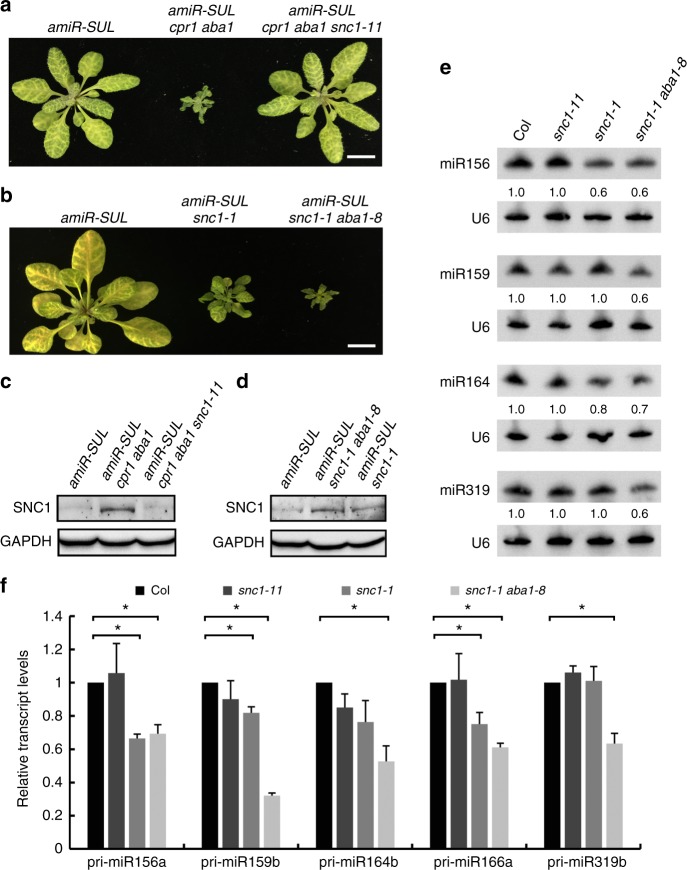
The R protein SNC1 underlies the *amiR-SUL* suppressor phenotype of *cpr1 aba1*. **a** Plants of the indicated genotypes. In contrast to *amiR-SUL cpr1 aba1*, *amiR-SUL cpr1 aba1 snc1–11* had the same phenotype as *amiR-SUL*, in terms of both plant size and leaf bleaching. Bar = 1 cm. **b** Plants of the indicated genotypes. The *amiR-SUL snc1–1* plants were smaller than *amiR-SUL* plants and showed a mild reduction in leaf bleaching. *amiR-SUL snc1–1 aba1–8* plants were similar to *amiR-SUL cpr1 aba1* (**a**) in both plant size and leaf bleaching. Bar = 1 cm. **c**–**d** Western blots to detect the SNC1 protein in the indicated genotypes. **c** The SNC1 protein was drastically elevated in abundance in *amiR-SUL cpr1 aba1* relative to *amiR-SUL*. **d** The SNC1 protein was elevated in abundance in *amiR-SUL snc1–1* relative to *amiR-SUL*, while the levels of SNC1 were not drastically different between *amiR-SUL snc1–1 aba1–8* and *amiR-SUL snc1–1*. GAPDH served as a loading control. **e** Northern blots for four miRNAs from 15-day-old seedlings of wild type (Col), *snc1–11*, *snc1–1* and *snc1–1 aba1–8*. U6 served as the loading control. The numbers indicate relative abundance. **f** The levels of five pri-miRNAs in wild type (Col), *snc1–11*, *snc1–1* and *snc1–1 aba1–8* as determined by real-time RT-PCR. Error bars represent standard deviation calculated from three biological replicates (^*^Student’s *t* test: *P* < 0.05). Source data are provided as a Source Data file

To test this hypothesis, we first crossed *amiR-SUL cpr1 aba1* with *snc1–11*, a loss-of-function mutant of *SNC1*^29^. In contrast to *amiR-SUL cpr1 aba1*, *amiR-SUL cpr1 aba1 snc1–11* had the same phenotype as *amiR-SUL*, in terms of both plant size and leaf bleaching area (Fig. 4a). Thus, loss of function in *SNC1* fully rescued the *amiR-SUL cpr1 aba1* phenotype, indicating that *CPR1* and *ABA1* promote amiR-SUL accumulation by suppressing *SNC1* expression. Consistent with this, the SNC1 protein abundance was drastically elevated in *amiR-SUL cpr1 aba1* relative to *amiR-SUL* (Fig. 4c).

The gain-of-function mutant *snc1–1* exhibits derepressed *SNC1* expression and constitutive defense responses^30^. We introduced *snc1–1* into the *amiR-SUL* background to evaluate its effects on amiR-SUL. As expected, the abundance of the SNC1 protein was elevated in *amiR-SUL snc1–1* relative to *amiR-SUL* (Fig. 4d). The *amiR-SUL snc1–1* plants were smaller than *amiR-SUL* plants and showed a mild reduction in leaf bleaching (Fig. 4b), but the phenotypes were much weaker than those of *amiR-SUL cpr1 aba1* (Fig. 4a). We performed small RNA sequencing of *amiR-SUL* and *amiR-SUL snc1–1* to determine the global profiles of miRNAs. Although some miRNAs were reduced in abundance in *amiR-SUL snc1–1* as compared to *amiR-SUL* (Supplementary Data 2), no significant global reduction in miRNA levels was observed (Supplementary Fig. 2). On the other hand, *amiR-SUL cpr1 aba1* showed a global reduction in miRNA accumulation as compared to either *amiR-SUL* or *amiR-SUL snc1–1* (Supplementary Fig. 2). Given that *aba1* mutations are expected to enhance the nuclear localization of SNC1, we reasoned that *aba1* mutations would enhance the *snc1–1* phenotype. Indeed, *amiR-SUL snc1–1 aba1–8* plants had stronger phenotypes than *amiR-SUL snc1–1* in both plant size and leaf bleaching (Fig. 4a, b). The levels of the SNC1 protein were not drastically different between *amiR-SUL snc1–1 aba1–8* and *amiR-SUL snc1–1* (Fig. 4d), consistent with the hypothesis that *aba1–8* promoted the nuclear localization rather than the abundance of the SNC1 protein.

To further evaluate the effects of *snc1–11*, *snc1–1*, and *snc1–1 aba1–8* mutations on endogenous miRNAs, we performed northern blotting in these genotypes for miR156, miR159, miR164, and miR319. These miRNAs were unaffected in *snc1–11*, but all of them showed a 30–40% reduction in *snc1–1 aba1–8*, and two of them (miR156 and miR164) were also reduced in *snc1–1* (Fig. 4e). We performed real-time RT-PCR to examine the levels of five pri-miRNAs (pri-miR156a, pri-miR159b, pri-miR164b, pri-miR166a, and pri-miR319b) in these genotypes. The pri-miRNAs were unaffected in *snc1–11*, while all of them were decreased by 35–60% in *snc1–1 aba1–8*. Three pri-miRNAs showed a significant reduction in *snc1–1* (Fig. 4f). Thus, the *aba1–8* mutation enhanced *snc1–1* in terms of its miRNA biogenesis defects. Taken together, these results suggested that the over accumulation and nuclear localization of SNC1 decrease the abundance of miRNAs by repressing the transcription of *MIR* genes.

### Induced SNC1 nuclear accumulation inhibits *MIR* transcription

Previous studies revealed a correlation between the nuclear abundance of SNC1 and its activity in disease resistance^28,31^. Our genetic studies led to the hypothesis that nuclear SNC1 represses miRNA biogenesis. To test this hypothesis, we sought to induce the nuclear accumulation of SNC1 at the transcriptional level through a β-estradiol-inducible promoter. A SNC1–NLS–GFP fusion was generated by inserting a nuclear localization signal (NLS) from the simian virus (SV40) large T-antigen^32^ between SNC1 and GFP, while NLS–GFP and SNC1–GFP were also generated for comparison.

**Fig. 5:**
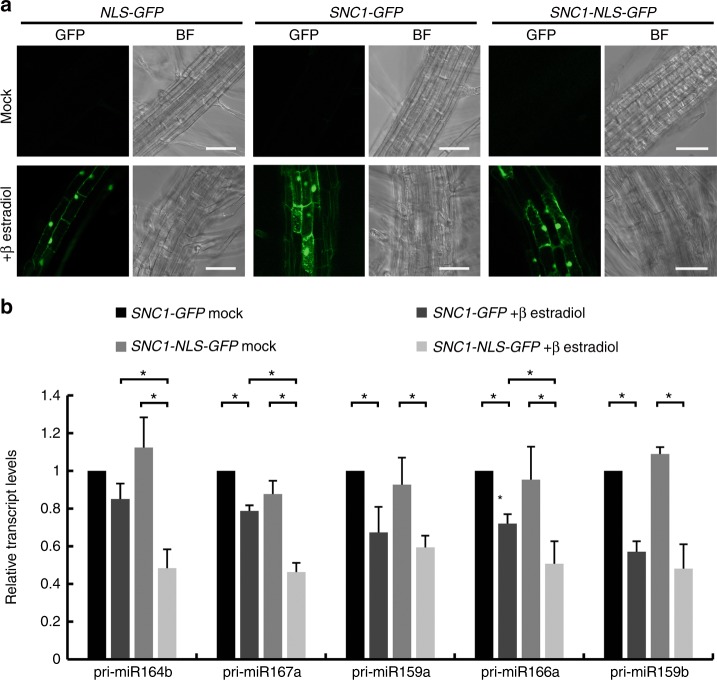
Induced SNC1 nuclear accumulation is sufficient to inhibit *MIR* gene expression. **a** *NLS-GFP*, *SNC1-GFP*, and *SNC1-NLS-GFP* transgenic seedlings were treated with DMSO (mock) or 50 μM β-estradiol (dissolved in DMSO). GFP fluorescence was detectable in roots of β-estradiol-treated but not mock-treated plants. BF bright field. Bar = 50 μm. **b** Relative levels of five pri-miRNAs in *SNC1-NLS-GFP* and *SNC1-GFP* after 6 h of mock and 50 μM β-estradiol treatment as determined by real-time RT-PCR. Error bars represent standard deviations calculated from three biological replicates (^*^Student’s *t* test: *P* < 0.05). Source data are provided as a Source Data file

*NLS–GFP, SNC1–GFP*, and *SNC1–NLS–GFP* transgenic seedlings were treated with DMSO or 50 μM β-estradiol (dissolved in DMSO). GFP fluorescence was detectable in root cells of β-estradiol-treated but not mock-treated plants (Fig. 5a). As expected, NLS–GFP was largely nuclear localized (Fig. 5a). SNC1–GFP and SNC1–NLS–GFP signals were present in both the nucleus and the cytoplasm, with the latter being more enriched in the nucleus (Fig. 5a). RNA was extracted from these plants and real-time RT-PCR was performed to determine the levels of five pri-miRNAs (pri-miR159a, pri-miR159b, pri-miR164b, primiR166a, and pri-miR167a). For *SNC1–GFP*, the levels of four of the five pri-miRNAs were reduced by 15–40% in β-estradiol-treated plants relative to mock-treated plants. For *SNC1–NLS–GFP*, the levels of all five pri-miRNAs were reduced by 35–57% in β-estradiol-treated plants relative to mock-treated plants (Fig. 5b). While the pri-miRNAs were similar in abundance in mock-treated *SNC1–NLS–GFP* and *SNC1–GFP* plants, three of the five pri-miRNAs were more severely affected in β-estradiol-treated *SNC1–NLS–GFP* plants than β-estradiol-treated *SNC1–GFP* plants. Thus, the nuclear accumulation of SNC1 represses the biogenesis of some miRNAs.

### The corepressor TPR1 represses *MIR* transcription

**Fig. 6:**
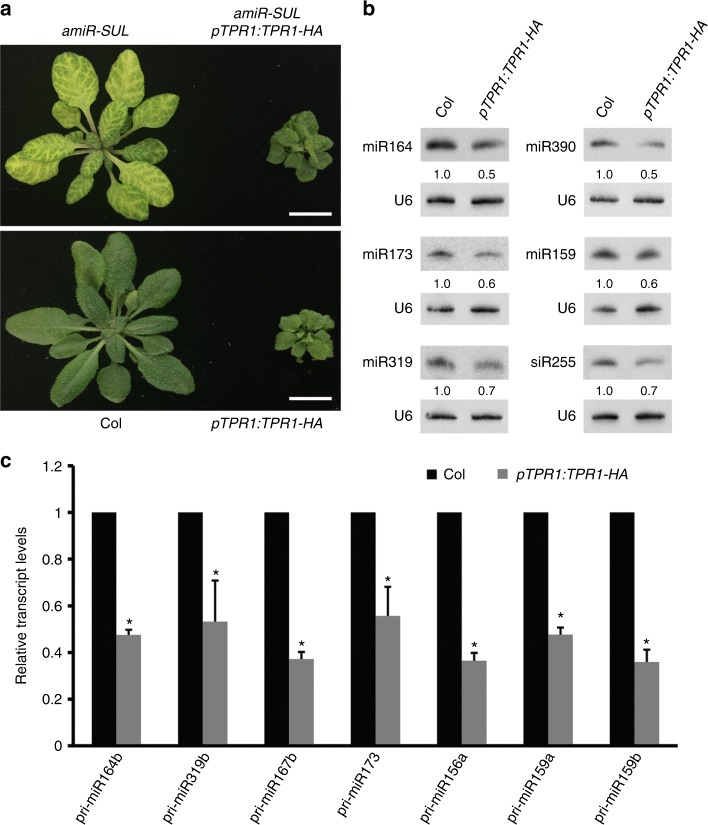
The transcriptional corepressor TPR1 represses the transcription of *MIR* genes. **a** Plants of the indicated genotypes. *pTPR1:TPR1-HA* plants were similar in plant size to *cpr1 aba1* plants (Fig. 2a). The *amiR-SUL pTPR1:TPR1-HA* plants displayed not only a dwarf phenotype but also reduced vein-centered bleaching. Bar = 1 cm. **b** Northern blots to detect five miRNAs and an endogenous siRNA (siR255 from *TAS1A/B/C*) in 15-day-old seedlings of wild type (Col) and *pTPR1:TPR1-HA* plants. Reduced accumulation was found for all these small RNAs in *pTPR1:TPR1-HA* plants. **c** Relative levels of seven pri-miRNAs in *pTPR1:TPR1-HA* vs. wild type (Col) as determined by real-time RT-PCR. Error bars represent standard deviations calculated from three biological replicates (^*^Student’s *t* test: *P* < 0.05). Source data are provided as a Source Data file

It was shown that nuclear SNC1 functions in defense responses through its association with TPR1, a transcriptional corepressor^33^. A *pTPR1:TPR1-HA* transgenic line with *TPR1* overexpression showed constitutive activation of defense responses^33^. *pTPR1:TPR1-HA* plants were similar in size to *cpr1 aba1* plants (Figs. 6a and 2a). To determine whether *TPR1* overexpression also phenocopies *cpr1 aba1* in terms of miRNA biogenesis defects, we crossed *amiR-SUL* with *pTPR1:TPR1-HA*. The *amiR-SUL pTPR1:TPR1-HA* plants displayed not only a dwarf phenotype but also reduced vein-centered bleaching (Fig. 6a). We investigated the abundance of endogenous miRNAs by northern blotting. The levels of miR164, miR173, miR319, miR390, and miR159 in 15-day-old seedlings of *pTPR1:TPR1-HA* were reduced to 50–70% of wild type levels (Fig. 6b). In addition, real-time RT-PCR showed that the levels of seven pri-miRNAs were markedly reduced in *pTPR1:TPR1-HA* (Fig. 6c). These results are consistent with the model that the nuclear accumulation of SNC1 in *cpr1 aba1* downregulates the abundance of miRNAs through the corepressor TPR1, which represses the transcription of *MIR* genes.

### Reduced phasiRNA production from *R* genes in *cpr1 aba1*

**Fig. 7:**
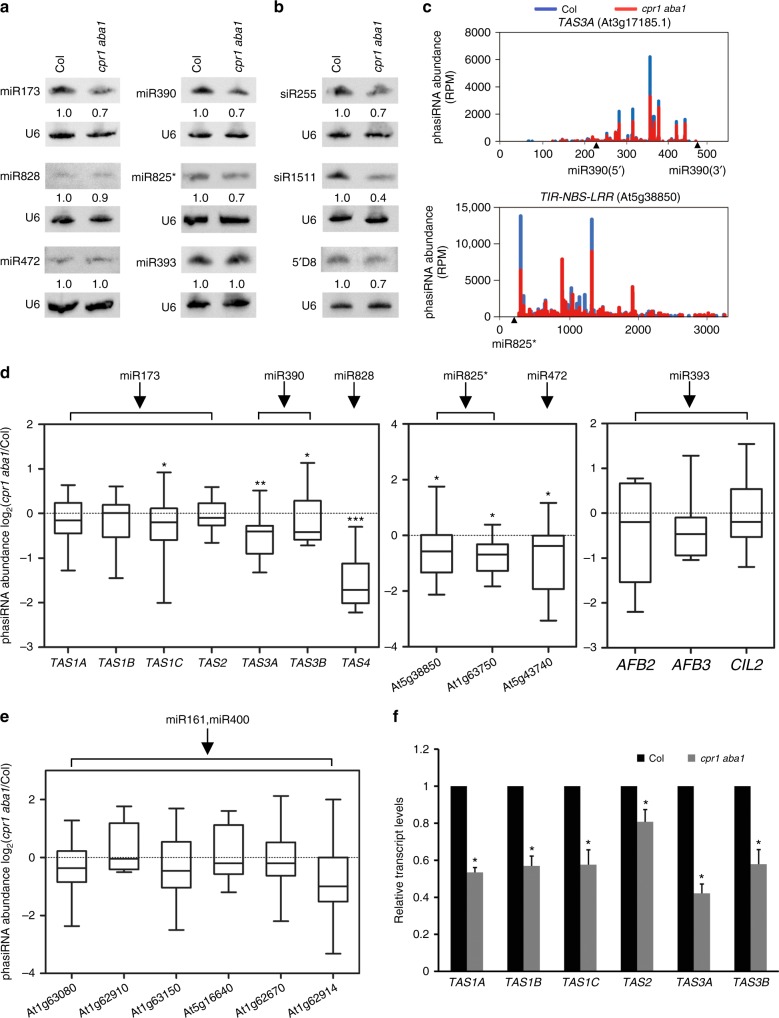
Accumulation of phasiRNAs from *R* genes is decreased in *cpr1 aba1*. **a** Northern blots to examine the accumulation of six miRNAs that are known to trigger phasiRNA biogenesis from their target transcripts. U6 served as a loading control. The numbers represent relative abundance. **b** Northern blots to determine the abundance of three tasiRNAs. siR255, siR1511 and 5’D8 are small RNAs from *TAS1A/B/C*, *TAS2*, and *TAS3A/B*, respectively. **c** PhasiRNAs from *TAS3A* (targeted by miR390) and an *R* gene (At5g38850; targeted by miR825*) in wild type (Col) and *cpr1 aba1* as determined by small RNA sequencing. The positions of the miRNA binding sites in the transcripts are indicated by the arrowheads. RPM (reads per million mapped reads). **d** Relative levels of phasiRNAs in *cpr1 aba1* vs. wild type (Col) as determined by small RNA sequencing. The phasiRNA-producing loci included seven *TAS* genes, three *NBS-LRR* genes (At5g38850, At5g43740 and At1g63750), *AFB2* (At3g26810), *AFB3* (At1g12820), and *CIL2* (At3g23690). The miRNAs that target these loci to trigger phasiRNA biogenesis are indicated. Significant difference between Col and *cpr1 aba1* is indicated by asterisks (Student’s *t* test: ^*^P < 0.05; ^**^P < 0.01; and ^***^P < 0.005). **e** The accumulation of phasiRNAs from six *PPR* genes (triggered by miR161/miR400) showed no significant difference between Col and *cpr1 aba1*. **f** The levels of *TAS* transcripts, which are precursors to tasiRNAs, were reduced in *cpr1 aba1*. Error bars represent standard deviations calculated from three biological replicates (^*^Student’s *t* test: *P* < 0.05). Source data are provided as a Source Data file

Some miRNAs target protein-coding or noncoding transcripts and trigger the production of phasiRNAs from these transcripts. Four families of noncoding *TAS* transcripts from eight loci generate phasiRNAs in *Arabidopsis*, among which *TAS1A/B/C* and *TAS2* are targeted by miR173, *TAS3A/B/C* are targeted by miR390, while *TAS4* is targeted by miR828^14,20,34–36^. Protein-coding loci also generate phasiRNAs, such as two *NBS-LRR* genes (At5g38850 and At5g43740) targeted by miR472 and/or miR825^*^^19,20^, the *PPR* family targeted by miR161 and/or miR400 (Fig. 7e), and *AFB2*, *AFB3*, and *CIL2* genes targeted by miR393^16,19,20^ (Fig. 7d). We tested the effect of *cpr1* and *aba1* mutations on the accumulation of three tasiRNAs, siR255 from *TAS1A/B/C*, siR1511 from *TAS2* and 5’D8 from *TAS3A/B*, by northern blotting. The levels of these small RNAs were lower in *cpr1 aba1* than those in Col (Fig. 7b). The levels of the miRNA triggers, such as miR173, miR390, and miR825^*^ were also deceased in *cpr1 aba1* (Fig. 7a), and this could be one factor that contributes to the reduced levels of tasiRNAs.

Next, we performed small RNA sequencing with Col and *cpr1 aba1* to determine the global effects of the *cpr1 aba1* mutations on phasiRNA biogenesis. The abundance of phasiRNAs generated from six *PPR* genes (triggered by miR161/miR400) as well as from *AFB2*, *AFB3*, and *CIL2* (triggered by miR393) showed no significant difference between *cpr1 aba1* and Col (Fig. 7d, e). However, the levels of tasiRNAs from four of the seven *TAS* loci were significantly reduced in *cpr1 aba1* (Fig. 7d). The reduction was obvious throughout the phasiRNA-generating regions of the *TAS* transcripts (Fig. 7c and Supplementary Fig. 3b), but phasing was not affected (Supplementary Fig. 3a). The reduction in tasiRNA levels was consistent with results from northern blotting (Fig. 7b).

The levels of *TAS* transcripts, which are precursors to tasiRNAs, were reduced in *cpr1 aba1*; this may be another factor that contributes to the reduced levels of tasiRNAs in *cpr1 aba1* (Fig. 7f). Besides, it is noteworthy that the levels of *TAS* transcripts were also reduced in *pTPR1:TPR1-HA* compared with Col (Supplementary Fig. 4), suggesting that overexpression of *TPR1* represses the transcription of *TAS* genes. The accumulation of siR255 (a tasiRNA from *TAS2*) was reduced in *pTPR1:TPR1-HA* (Fig. 6b). Therefore, it is reasonable to speculate that over-accumulated and nuclear localized SNC1 in *cpr1 aba1* acts with TPR1 to reduce tasiRNA biogenesis, by repressing the expression of both the miRNA triggers and the *TAS* loci.

In addition to At5g38850 and At5g43740, our small RNA sequencing revealed a third *R* gene, At1g63750, which generates phasiRNAs, with miR825^*^ serving as the potential trigger (see Supplementary Data 3 for a complete list of phasiRNA-generating loci analyzed in this study). The levels of phasiRNAs from the three *NBS-LRR* genes (At5g38850, At5g43740, and At1g63750) were all decreased in *cpr1 aba1* (Fig. 7c, d and Supplementary Fig. 3c).

### Widespread induction of *NBS-LRR* genes in *cpr1 aba1*

**Fig. 8:**
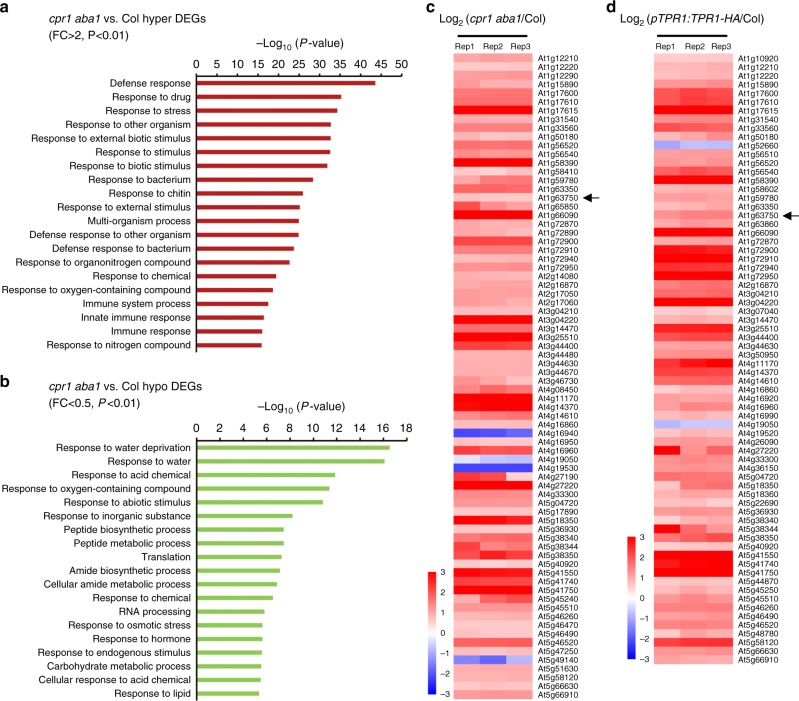
Widespread induction of *NBS-LRR* genes in *cpr1 aba1*. **a** The top 20 GO terms for up-regulated genes (hyper DEGs) in *cpr1 aba1* as determined by RNA-seq. *R* genes with RPM > 10 in either *cpr1 aba1* or wild type (average of three replicates) were used for the identification of hyper DEGs (fold-change > 2, *P* value < 0.01). The hyper DEGs are enriched for genes involved in stress responses, particularly defense responses. **b** The top 19 GO terms for downregulated genes (hypo DEGs) in *cpr1 aba1*. *R* genes with RPM > 10 in either *cpr1 aba1* or wild type (average of three replicates) were used for the identification of hypo DEGs (fold-change < 0.5, *P* value < 0.01). GO terms in various small molecule biosynthetic and metabolic processes are enriched in the hypo DEGs. **c** Analysis of *R* gene expression in three biological replicates (rep1–3) of RNA-seq of *cpr1 aba1* and wild type (Col). Only DEGs with fold-change > 1.5, *P* value < 0.05 and RPM > 10 in either genotype (average of three replicates) were included in the analysis. Among the DEGs, 70 and 4 were upregulated and downregulated, respectively, in *cpr1 aba1* relative to Col. The arrow marked an *R* gene (At1g63750) that could generate phasiRNAs. **d** Analysis of *R* gene expression in three biological replicates (Rep1–3) of RNA-seq of *pTPR1:TPR1-HA* and wild type (Col). Only DEGs with fold-change > 1.5, *P* value < 0.05 and RPM > 10 in either genotype (average of three replicates) were included in the analysis. Among the DEGs, 68 and 2 were upregulated and down-regulated, respectively, in *pTPR1:TPR1-HA* relative to Col. The arrow marked an *R* gene (At1g63750) that could generate phasiRNAs

To examine the effects of *cpr1* and *aba1* mutations on the transcriptome, either through SNC1-TPR1 or indirectly through phasiRNAs, we performed RNA-seq in wild type and *cpr1 aba1* in three biological replicates. Totally, 1743 and 1020 upregulated (hyper DEGs) and downregulated (hypo DEGs) genes, respectively, were identified in *cpr1 aba1* (Supplementary Data 4 and 5). The hyper DEGs were enriched in genes involved in stress responses including defense responses (Fig. 8a), consistent with previous studies on *snc1–1* or *cpr1*^22,30,37^. GO terms in various small molecule biosynthetic and metabolic processes were enriched in the hypo DEGs (Fig. 8b). RNA-seq was also performed for wild type and *pTPR1:TPR1-HA*, and 1719 upregulated genes (hyper DEGs) were found in the latter (Supplementary Data 6). The hyper DEGs were also enriched in GO terms related to stress responses (Supplementary Fig. 5a). Totally, 1000 genes were shared by the hyper DEGs in *cpr1 aba1* and those in *pTPR1:TPR1-HA*, and GO analysis showed that they were also related to defense responses and other stress responses (Supplementary Fig. 5b and Supplementary Data 7).

As we observed a reduction in phasiRNAs from three *NBS-LRR* genes in *cpr1 aba1*, and as the phasiRNAs may target other *R* genes, we interrogated the expression of all *NBS-LRR* genes. There were 165*R* genes with RNA-seq reads in *cpr1 aba1* or Col, and we chose 156*R* genes with reads per million mapped reads (RPM) > 10 in either genotype (average of three replicates) for further analysis (Supplementary Data 8). The transcript levels of most *R* genes were increased in *cpr1 aba1* relative to those in Col (Supplementary Fig. 6a). Totally, 70 and 4 upregulated and downregulated *R* genes in *cpr1 aba1* compared with Col, respectively, were identified at fold-change > 1.5 and *P* < 0.05 (Fig. 8c and Supplementary Data 9). We also examined *R* gene expression in *pTPR1:TPR1-HA* and Col. Among 166*R* genes with RNA-seq reads in *pTPR1:TPR1-HA* or Col, 151*R* genes with RPM > 10 in either genotype (average of three replicates) were chosen for further analysis (Supplementary Data 10). Most *R* genes were expressed at higher levels in *pTPR1:TPR1-HA* relative to Col (Supplementary Fig. 6b). Totally, 68 and 2 upregulated and downregulated *R* genes in *pTPR1:TPR1-HA* relative to Col, respectively, were identified at fold-change > 1.5 and *P* < 0.05 (Fig. 8d and Supplementary Data 11). Thus, an overwhelming increase in *R* gene expression occurred in both *cpr1 aba1* and *pTPR1:TPR1-HA*.

To determine whether phasiRNAs generated from the three source *R* genes can target other *R* genes, 47 top abundant phasiRNAs from the three loci in wild type (Col) were used for target prediction with psRNATarget^38,39^. The abundance cutoff was normalized RPM no less than 500; note that this abundance was comparable to, or higher than, that of tasiRNAs from *TAS3B* (Supplementary Fig. 3b). Totally, 93*R* genes (including the three source *R* genes) were found to be potential targets of these phasiRNAs (Supplementary Data 12). Therefore, phasiRNAs from the three source *R* genes can theoretically target many *R* genes *in trans*.

Among the three *R* genes (At1g63750, At5g38850, and At5g43740) that generate phasiRNAs, At1g63750, and At5g38850 were upregulated in both *cpr1 aba1* and *pTPR1:TPR1-HA* as determined by RNA-seq, while At5g43740 was downregulated (Supplementary Fig. 6a, b). However, the change in At5g38850 expression was small and not independently observed by real-time RT-PCR as for the other two genes (Supplementary Fig. 7a, b). We propose that the upregulation of At1g63750 and maybe At5g38850 in *cpr1 aba1* be caused by the reduced levels of miR825^*^ (Fig. 7a), which targets these genes. The downregulation of At5g43740 was rather unexpected and may be due to repression by SNC1 or indirect effects.

The widespread induction of *NBS-LRR* genes in *cpr1 aba1* indicated that *cpr1 aba1* may exhibit enhanced resistance to pathogens. We tested the resistance of *cpr1–4* and *aba1–8* plants to *Pst* DC3000 and *Pst* DC3000(*AvrRps4*). The *cpr1 aba1* double mutant was not included due to its small size, which makes it difficult to do bacterial inoculation. We observed that the *aba1–8* mutant was significantly more resistant to both bacterial strains compared to wild-type plants (Supplementary Fig. 8a–d). On the contrary, the *cpr1–4* mutant was similar to wild type (Supplementary Fig. 8a–d). The *cpr1–2* mutant was shown to be more resistant to *Pst* DC3000 and *Pst* DC3000(*AvrRpt2*)^25,40^. Perhaps, the lack of resistance of *cpr1–4* is due to its being a weaker allele. An *aba1* mutant (*aba1–6*) was found to be more resistant to *Pst* DC3000(*AvrRps4*)^28^. Thus, loss (or reduction) of function in *ABA1* leads to enhanced bacterial resistance.

## Discussion

NBS-LRR proteins (known as R proteins) recognize pathogen infection and trigger plant defense. It has been largely unknown how R proteins affect the small noncoding portion of the transcriptome. In this study, we isolated a *cpr1 aba1* double mutant in a genetic screen as defective in miRNA biogenesis. Defects in miRNA biogenesis in the double mutant are likely attributable to reduced transcription of *MIR* genes, as pri-miRNA levels as well as *MIR167a* promoter activity are reduced in the mutant. The F-box protein CPR1 negatively regulates the activation and accumulation of the R protein SNC1^25^. Nuclear localization of SNC1 is required for disease resistance^31^ and ABA deficiency promotes the activity and nuclear localization of SNC1^28^. This suggests that the phenotypes of the *cpr1 aba1* double mutant could be caused by over accumulation of nuclear SNC1. Indeed, SNC1 protein over accumulated in the double mutant and loss of function in *SNC1* fully rescued the double mutant’s phenotypes. Besides, induced nuclear accumulation of SNC1 (in *SNC1–NLS–GFP* plants) resulted in reduced levels of pri-miRNAs. Nuclear SNC1 functions in plant defense through its association with TPR1, a transcriptional corepressor^33^. In this study, we found that *TPR1* overexpression also led to reduced levels of miRNAs as well as pri-miRNAs. Thus, the R protein SNC1 probably represses *MIR* gene expression in the nucleus through TPR1.

We found that nuclear accumulation of SNC1 is also associated with reduced levels of phasiRNAs from noncoding *TAS* loci. This could be due to the combined effects of reduced abundance of the miRNAs that trigger phasiRNA biogenesis and repression of *TAS* transcription leading to decreased levels of phasiRNA precursors. In addition to noncoding *TAS* loci, some protein-coding genes including *R* genes give rise to phasiRNAs. In Legumes and Solanaceae species, phasiRNAs are produced from many *R* genes^9,11,17^. In *Arabidopsis*, three *R* genes (At5g38850, At5g43740, and At1g63750) among the ~170*R* genes produce phasiRNAs. The abundance of the phasiRNAs produced from the three loci was decreased in *cpr1 aba1*. On the other hand, a global de-repression of *R* gene expression was observed in the double mutant. Totally, 93*R* genes were predicted to be potential targets of some of the most abundant phasiRNAs produced from the three *R* genes. Thus, the upregulation of most *R* genes in *cpr1 aba1* may be caused, or at least conditioned, by the decreased levels of phasiRNAs from the three *R* genes.

Plants need to properly allocate resources toward growth and defense. In the absence of pathogen infection, *R* genes are usually repressed to suppress defense responses and enable growth. This is in part achieved through a miRNA-phasiRNA cascade, in which a few miRNAs target some *R* genes, leading to the production of phasiRNAs that perhaps target the entire *R* gene family. Here, we uncovered another side of the regulatory network. The activation of the R protein SNC1 leads to repression of miRNA and phasiRNA biogenesis to likely release the global repression of *R* gene expression to enable defense (Supplementary Fig. 9). The release of the repression itself may not be sufficient for global *R* gene induction, but allows for such induction to be triggered by other mechanisms.

## Methods

### Plant materials and growth conditions

The *amiR-SUL sup*-*B65* mutant was isolated from an EMS mutagenized population of a transgenic *Arabidopsis thaliana* line *proSUC2:amiR-SUL* (*amiR-SUL*; a gift from Dr. Detlef Weigel) in which an artificial miRNA (amiR-SUL) is expressed from the *SUCROSE-PROTON SYMPORTER 2* (*SUC2*) promoter^21^. The *cpr1* and *aba1* mutations from the *amiR-SUL sup*-*B65* line were named *cpr1–4* and *aba1–8*, respectively. The following transgenic lines or mutants were used in this study: *pMIR167a:GUS*^23,24^, *pTPR1:TPR1-HA*^33^, SALK_045148 (*cpr1–3*;^22^), SALK_059469 (which we named *aba1–7*), *snc1–11*^29^, and *snc1–1*^30^. The *amiR-SUL snc1–1 aba1–8* and *amiR-SUL cpr1 aba1 snc1–11* genotypes were obtained by crosses. The *cpr1–4 aba1–8* double mutant was generated by crossing *amiR-SUL sup-B65* with Columbia (Col) wild-type plants. All seeds were grown on petri dishes containing 1/2 Murashige and Skoog Basal Medium (Sigma-Aldrich, M5519) plus 2% Sucrose and 0.8% Agar. All plants were grown in growth chambers at 22 °C with 16 h light and 8 h dark cycles.

### Identification of mutated genes in *amiR-SUL sup-B65*

The *amiR-SUL sup-B65* mutant was backcrossed to its parental line *amiR-SUL*. In the F2 generation, 72 individual plants with the same phenotype as the *amiR-SUL sup-B65* mutant were used for genomic DNA extraction with the CTAB method^41^. An equal amount of DNA from each plant was pooled for genomic DNA library construction at Beijing Genomics Institute (BGI), Shenzhen, China. The library was sequenced with the paired-ended (PE150bp) scheme on an Illumina HiSeq4000 platform at 50x coverage at BGI. A mutation in *CPR1* (At4g12560), which we designated *cpr1–4*, and another mutation in *ABA1* (At5g63070), which we designated *aba1–8*, were identified in *amiR-SUL sup-B65*. The *cpr1–4* mutation abolishes a *Sac*I restriction enzyme site, while the *aba1–8* mutation generates a *Stu*I restriction enzyme site. This formed the basis of molecular genotyping of the mutations. The primers used to amplify genomic DNA to genotype the mutations are listed in Supplementary Table 1.

### DNA constructs and complementation tests

The genomic region of *CPR1* was amplified using primers proCPR1-KpnI-F and CPR1-PstI-R from Col genomic DNA; the resulting fragment contains the entire *CPR1* gene body without the stop codon plus 2000 bp of sequences upstream of the ATG start codon. The *proCPR1:CPR1* fragment was fused with eYFP in the binary vector pGWB640^42^ via LR clonase (Invitrogen) to generate *pCPR1:CPR1-eYFP*. The genomic region of *ABA1* was amplified from the Col genomic DNA with primers proABA1-KpnI-F and ABA1-SpeI-R; and the resulting fragment contains the entire *ABA1* gene body without the stop codon plus 945 bp of sequence upstream of the ATG start codon. The *proABA1:ABA1* fragment was fused with GFP in the binary vector pMDC107^43^ via LR clonase (Invitrogen) to generate *pABA1:ABA1-GFP*. The two plasmids were introduced into the *Agrobacterium tumefaciens* strain GV3101. The Agrobacteria were then used to transform *amiR-SUL cpr1 aba1* and *cpr1 aba1* by the floral-dip method^44^. The primers for cloning are listed in Supplementary Table 2.

A fragment (ATGGCGCCAAAAAAGAAGAGAAAGGTC) encoding an NLS^32^ was fused with GFP by PCR with primers PstI-NLS-BamHI-GFP-F and GFP-SacI-R to construct an NLS-GFP fragment. The NLS-GFP fragment was cloned into pMDC7^43^ via LR clonase II (Invitrogen) to generate pMDC7-NLS-GFP. The genomic region without the stop codon of *SNC1* (At4g16890) was amplified from Col genomic DNA with primers SNC1-KpnI-F and SNC1-PstI-R, and fused with NLS-GFP. The SNC1-NLS-GFP fragment and the SNC1 fragment were cloned into pMDC7 via LR clonase II (Invitrogen) to generate pMDC7-SNC1-NLS-GFP and pMDC7-SNC1-GFP, respectively. The pMDC7 vector allows for β-estradiol-inducible, ectopic expression. The three plasmids were used to transform Col plants through *Agrobacterium*-mediated transformation^44^. Transgenic plants were named *NLS*-*GFP*, *SNC1-NLS-GFP*, and *SNC1-GFP* for simplicity. The primers used for cloning are listed in Supplementary Table 2.

### Real-time RT-PCR

Real-time RT-PCR was performed to quantify pri-miRNA and mRNA levels. One microgram total RNA was reversed transcribed with oligo (dT) using the PrimeScript^™^ 1st Strand cDNA Synthesis Kit (TAKARA, 6110A) according to manufacturer’s instructions. Quantitative RT-PCR was performed with the 96-well StepOne Plus real-time system (Applied Biosystems) using the SYBR premix ExTaq II kit (TAKARA, RR820A). The following amplification scheme was used: 95 °C for 30 s and 40 cycles of 95 °C for 5 s and 60 °C for 30 s. The expression levels of target transcripts were normalized to the level of the internal standard *UBQ5* and the 2^−△△CT^ values of control samples were set to 1. All primers used in the assays are listed in Supplementary Table 3. Three independent biological replicates were carried out for each genotype or treatment, and Student’s *t* test was used to evaluate statistical significance.

### Small RNA northern blots

Total RNAs were isolated from 15-day-old seedlings with TRI reagent (Molecular Research Center, TR118). For northern blotting, 10 μg total RNA was resolved on 15% polyacrylamide gels with 8 M urea. After gel electrophoresis, the RNAs were transferred to a Hybond-NX nylon membrane (GE healthcare). Oligonucleotide probes complementary to the miRNAs or tasiRNAs to be detected were synthesized with both 5′ and 3′ end-labeled biotin. A probe complementary to U6 was used as an internal control. Hybridization was performed for 16 h at 55 °C followed by washes. Signals were detected using the chemiluminescent Nucleic Acid Detection Module (Thermo Fisher, 89880) with a Chemiluminescence imaging system (Clinx Science Instruments Co. Ltd., China). The probes used are listed in Supplementary Table 4.

### Western blots

Western blotting was performed using proteins from 15-day-old seedlings. Anti-SNC1 polyclonal antibody produced in rabbit was generated against a SNC1-specific peptide (RKTMTPSDDFGDC) at GenScript. Other antibodies used in immunoblotting experiments include anti-SUL^45^ (dilution, 1:1000) and anti-GAPDH (Santa Cruz Biotechnology, sc-365062) (dilution, 1:1000). The secondary antibodies were horseradish peroxidase-conjugated goat-anti-rabbit IgG (Bio-Rad, cat.#172-1019) (dilution, 1:2000) and goat-anti-mouse IgG (Bio-Rad, cat.#170-6516) (dilution, 1:2000). The protein signals were detected using the Amersham^TM^ ECL^TM^ Prime Western Blotting Detection Reagent (GE healthcare, RPN2232) using a Chemiluminescence imaging system (Clinx Science Instruments Co. Ltd., China).

### Histochemical GUS staining

Fifteen-day-old seedlings of *pMIR167a:GUS* and *cpr1 aba1 pMIR167a:GUS* (homozygous for both the transgene and the mutant genotypes) were subjected to histochemical GUS staining according to the standard protocol^46^.

### β-estradiol treatment

15-day-old transgenic seedlings of *SNC1-NLS-GFP* and *NLS-GFP* grown on solid medium were transferred to 1/2 MS liquid medium containing 50 μM β-estradiol and incubated for 6 h. The treated seedlings were then subjected to fluorescence observation or RNA extraction.

### Bacterial infection assays

Rosette leaves of five-week-old plants were infiltrated with bacterial suspensions of *Pst* DC3000 or *Pst* DC3000(*avrRps4*) at OD_600_ = 0.001 using a needleless syringe. Inoculated plants were kept in a growth chamber with high humidity (~90%). Leaf discs from inoculated leaves were collected at day 0 and day 3 and ground in 10 mM MgCl_2_. Serial dilutions were plated on selective medium, and bacterial populations were determined as colony forming units (cfu) per cm^2^ leaves^47^.

### Fluorescence microscopy

For the visualization of NLS-GFP, SNC1-GFP, and SNC1-NLS-GFP, roots of 15-day-old transgenic seedlings were observed under a Zeiss LSM 5 Pascal inverted confocal microscope.

### Small RNA sequencing and data analysis

For library construction, 30 μg total RNA was resolved on a 15% polyacrylamide gel with 8 M urea. Small RNAs in the 15–40 nt size range were recovered as described^48^. Small RNA libraries were prepared using the NEBNext Multiplex Small RNA Libraty Prep Set for Illumina (New England Biolabs, E7300), and sequenced with an Illumina HiSeq2500 platform at BerryGenomics, China. Raw reads (SE50) were trimmed using Perl scripts to remove adapters. The clean reads were mapped to the *Arabidopsis thaliana* genome TAIR10 using the Bowtie program^49^. The small RNA reads were calculated and normalized in reads per million mapped reads (RPM). The comparison between samples was conducted with the R package edgeR^50^.

For analysis of miRNA levels, small RNA reads were mapped to annotated miRNAs in miRBase v21. For analysis of tasiRNAs levels, small RNA reads were mapped to each of the eight annotated *TAS* genes (*TAS1A*/*1B*/*1C*, *TAS2*, *TAS3A*/*3B/3C*, and *TAS4*). For the analysis of phasiRNAs from protein coding genes, such as *R* genes and *PPR* genes, small RNA reads were mapped to the genes known to produce phasiRNAs and only uniquely mapped reads were included in subsequent analyses. Phasing analysis was performed as described^51^. Small RNA reads from sense and antisense strands were unified. Phasing score was calculated by the following formula:

$${\mathrm{Phasing}}\,{\mathrm{score}} = {\mathrm{ln}}\left[ {\left( {1 + 10 \times \frac{{\mathop {\sum }\nolimits_{i = 1}^{10} Pi}}{{1 + {\sum} U }}} \right)^{n - 2}} \right],{n} > 3,$$

where *n* = number of phase cycle positions occupied by no less than one read within a 10-cycle window; *P* = total number of all small RNA reads with start coordinates in a given phase within a 10-cycle window; *U* = total number of small RNA reads with start coordinates out of a given phase. In our analysis, phase cycle length was set at 21 nt.

For predicting *R* genes that can be potentially targeted by phasiRNAs generated from the three source *R* genes, 47 top abundant phasiRNAs (normalized reads with RPM no less than 500) from small RNA sequencing of wild type (Col) were used for target prediction with the online server psRNATarget (http://plantgrn.noble.org/psRNATarget/)^38,39^. The score for complementarity between phasiRNA and target mRNA sequences was set to be no more than 4.

### RNA-seq data analysis

Total RNAs extracted from 15-day-old seedlings of wild type and mutants were sent to Novogene, China, for mRNA-seq library construction and the libraries were sequenced on an Illumina Hiseq ×10 platform to generate single-end reads of 150 bp in length. The analysis was performed by a homemade pipeline PIHSDA (https://github.com/grubbybio/PIHSDA/blob/master/pihsda). Clean reads were trimmed to remove adapter sequences and mapped to *Arabidopsis* genome TAIR 10 using HISAT2 with default settings^52^. Transcript levels were calculated and normalized in RPM. Then gene expression levels were normalized and differentially expressed genes (DEGs) were calculated with the cut-off of fold-change > 1.5 (for hyper DEGs) or fold-change < 0.67 (for hypo-DEGs), and *P* < 0.05 using DEseq2^53^. For GO-terms analysis, only genes with fold-change > 2.0 (for hyper DEGs) or fold-change < 0.5 (for hypo-DEGs), *P* < 0.01, and RPM > 10 in either genotype (average of three replicates for each genotype) were included. For heatmap figures, *R* genes with fold-change > 1.5 (for hyper DEGs) or fold-change < 0.67 (for hypo-DEGs), *P* < 0.05, and RPM > 10 in either genotype (average of three replicates for each genotype) were included. The GO-term enrichment analysis was performed online at TAIR (http://www.arabidopsis.org/tools/go_term_enrichment.jsp). Only the top 20 terms were presented in this paper.

## Electronic supplementary material

Supplementary Information

Description of Additional Supplementary Files

Supplementary Data 1

Supplementary Data 2

Supplementary Data 3

Supplementary Data 4

Supplementary Data 5

Supplementary Data 6

Supplementary Data 7

Supplementary Data 8

Supplementary Data 9

Supplementary Data 10

Supplementary Data 11

Supplementary Data 12

Supplementary Data 13

Reporting Summary

## Data Availability

All raw data and processed data were deposited in NCBI GEO with the accession number GSE111240 [https://www.ncbi.nlm.nih.gov/geo/query/acc.cgi?acc = GSE111240]. All other data supporting the findings of this study are available within the manuscript and its supplementary files or are available from the corresponding authors upon request. A reporting summary for this Article is available as a Supplementary Information file. The source data underlying Figs. 1d, 2b–d, 3a, b, d, 4c–f, 5b, 6b, c, 7a, b and f, and Supplementary Figs. 4, 7a, 7b, 8b, and 8d are provided as a Source Data file (Supplementary Data 13).
