# Author Correction: The disease resistance protein SNC1 represses the biogenesis of microRNAs and phased siRNAs

**DOI:** 10.1038/s41467-019-08627-x

**Published:** 2019-02-04

**Authors:** Qiang Cai, Chao Liang, Suikang Wang, Yingnan Hou, Lei Gao, Li Liu, Wenrong He, Wenbo Ma, Beixin Mo, Xuemei Chen

**Affiliations:** 1 Guangdong Provincial Key Laboratory for Plant Epigenetics, College of Life Sciences and Oceanography, Shenzhen University, Shenzhen, 518060 China; 2 Key Laboratory of Optoelectronic Devices and Systems of Ministry of Education and Guangdong Province, College of Optoelectronic Engineering, Shenzhen University, Shenzhen, 518060 China; 3 Department of Botany and Plant Sciences, Institute of Integrative Genome Biology, University of California, Riverside, CA 92521 USA; 4 Department of Microbiology and Plant Pathology, University of California, Riverside, CA 92521 USA

Correction to: *Nature Communications*; 10.1038/s41467-018-07516-z; published online 29 November 2018.

The original version of this Article contained an error in the spelling of the author Beixin Mo, which was incorrectly given as Beixing Mo. This has now been corrected in both the PDF and HTML versions of the Article.

