# Supplementary material for: The disease resistance protein SNC1 represses the biogenesis of microRNAs and phased siRNAs
Source: Nat Commun. 2018 Nov 29;9:5080. doi: 10.1038/s41467-018-07516-z (PMC6265325; doi:10.1038/s41467-018-07516-z)
Supplement: Supplementary file 1 — Supplementary Information [file 41467_2018_7516_MOESM1_ESM.pdf]

The disease resistance protein SNC1 represses the biogenesis of microRNAs and phased siRNAs

Cai et al.

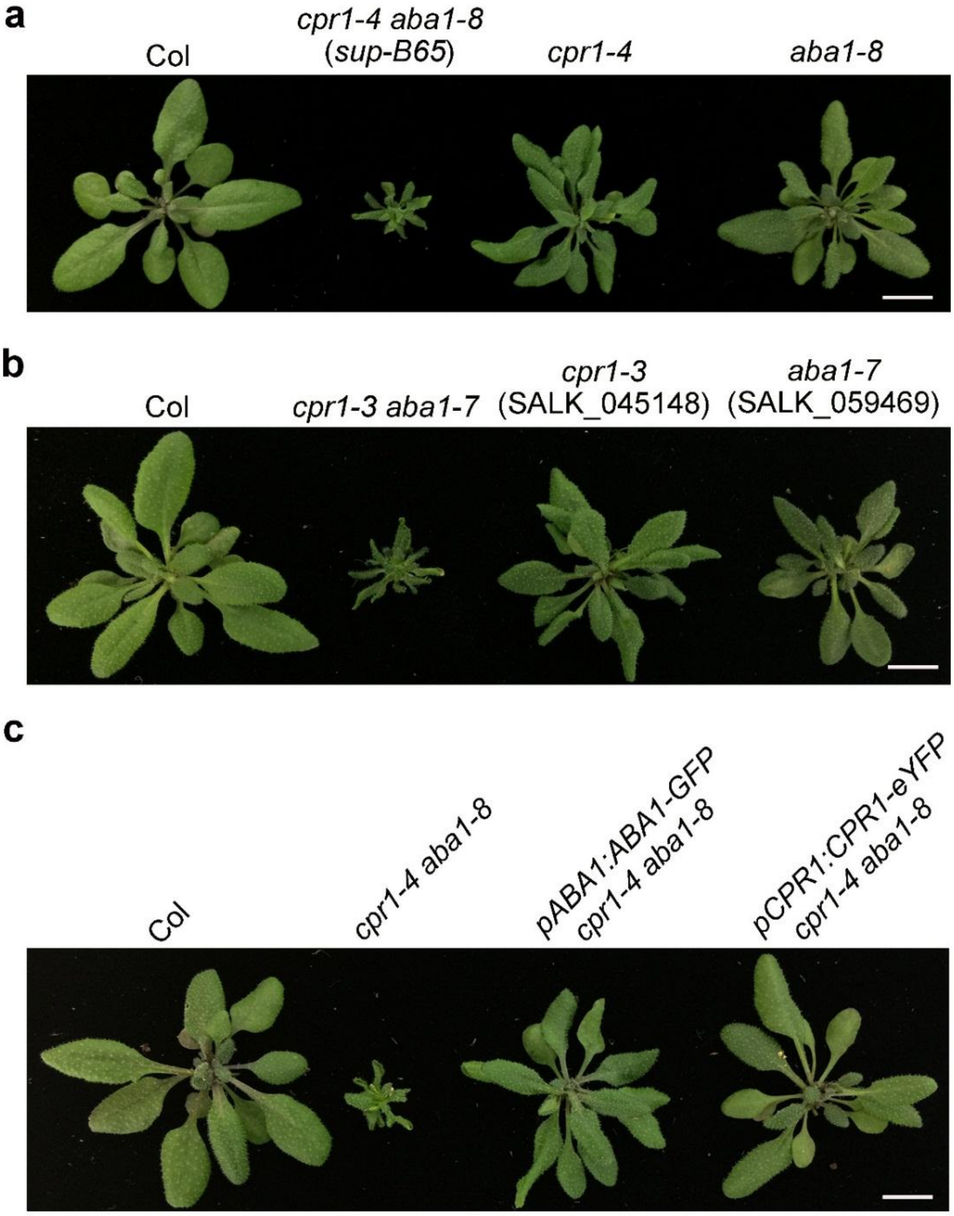

Supplementary Figure 1. Phenotypes of mutants and complementation lines. **a** Plants of the *cpr1-4*, *aba1-8* and *cpr1-4 aba1-8* genotypes were obtained from the cross between wild type and *amiR-SUL sup-B65*. Bar = 1cm. **b** Two T-DNA insertion lines, SALK_045148 (*cpr1-3*) and SALK_059469 (which we named *aba1-7*), were crossed with each other to generate the *cpr1-3 aba1-7* double mutant. The double mutant *cpr1-3 aba1-7*, but not either single mutant, had drastically reduced plant size resembling *cpr1-4 aba1-8* in **a**. Bar = 1cm. **c** The *cpr1-4 aba1-8* double mutant was transformed with *pCPR1:CPR1-eYFP* or *pABA1:ABA1-GFP*. Either transgene fully rescued the morphological defects of *cpr1-4 aba1-8*. Bar = 1cm.

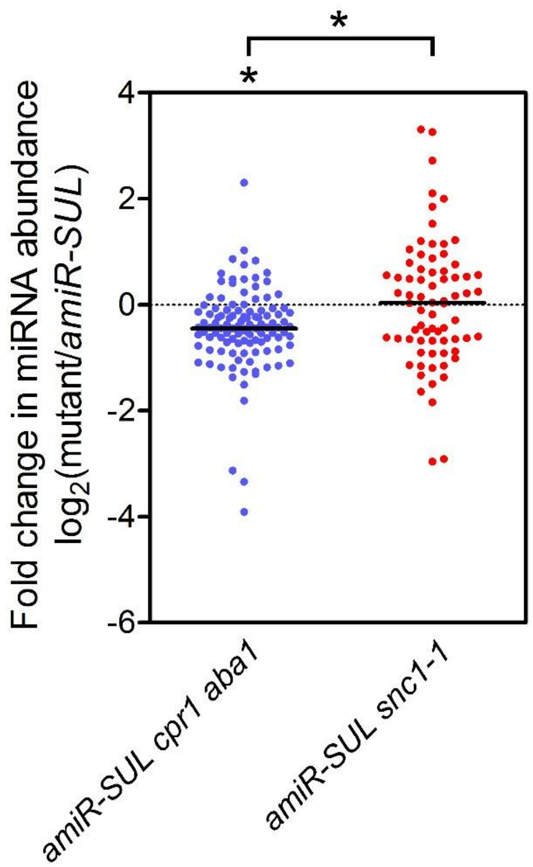

Supplementary Figure 2. Global abundance of miRNAs in *amiR-SUL*, *amiR-SUL cpr1 aba1* and *amiR-SUL snc1-1* as determined by small RNA sequencing. Small RNA libraries were generated from 15-day-old seedlings in three biological replicates. The $\log_2$ ratio of mutant/*amiR-SUL* was plotted, with each dot representing a miRNA. The $\log_2$ ratio for each miRNA was the mean from three biological replicates. The black lines represent the median. Statistical significance was evaluated by Student's *t* test, * $P < 0.05$.

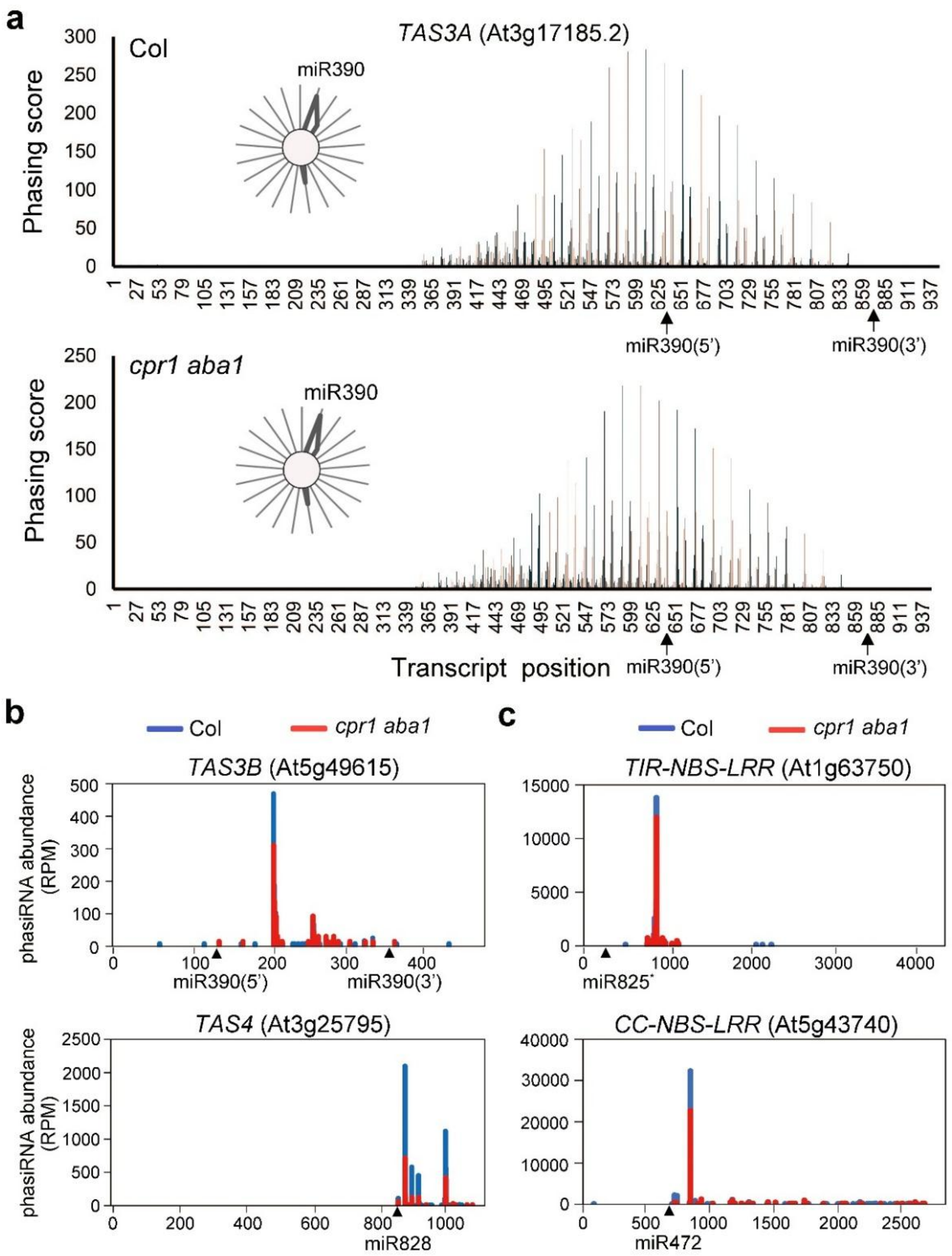

Supplementary Figure 3. Distribution and abundance of 21-nucleotide phasiRNAs from a few loci. **a** The phasing of tasiRNAs from *TAS3A* is unaffected in *cpr1 aba1*. The X-axis depicts the *TAS3A* locus with the positions of the two miR390 target sites marked by arrows. The radial graphs show that the 5' miR390 cleavage site fell in the most prominent phasing register of 21-nt small RNAs in Col as well as in *cpr1 aba1*. **b** The accumulation of tasiRNAs from *TAS3B* and *TAS4* as determined by small RNA sequencing. The abundance of tasiRNAs from these loci was reduced in *cpr1 aba1*

relative to Col. The positions of the miRNA target sites are marked. **c** The abundance of phasiRNAs from two *NB-LRR* loci (At1g63750 and At5g43740) was reduced in *cpr1 aba1* relative to Col. The positions of the miRNA target sites are marked.

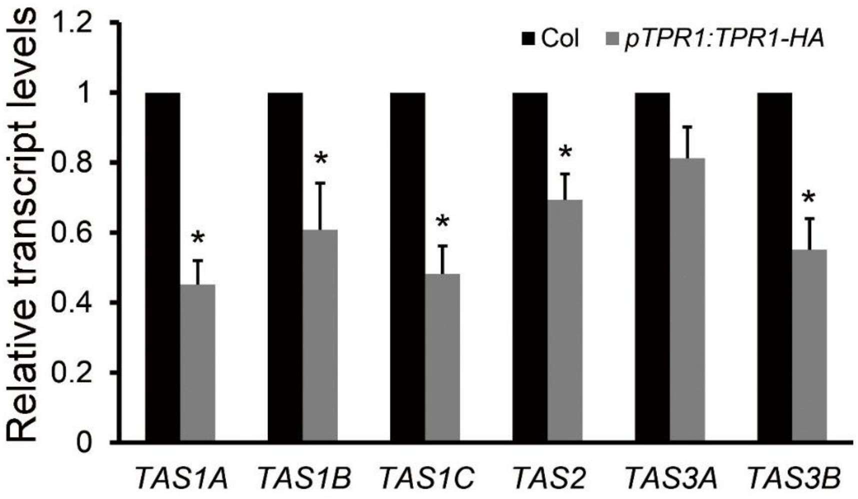

Supplementary Figure 4. The transcript levels of *TAS* genes in *pTPR1:TPR1-HA* and Col. The transcript levels of *TAS1A*, *TAS1B*, *TAS1C*, *TAS2*, *TAS3A* and *TAS3B* were determined by real-time RT-PCR in 15-day-old seedlings of *pTPR1:TPR1-HA* and Col. Error bars represent standard deviations calculated from three biological replicates (*Student's *t* test: $P < 0.05$). Source data are provided as a Source Data file.

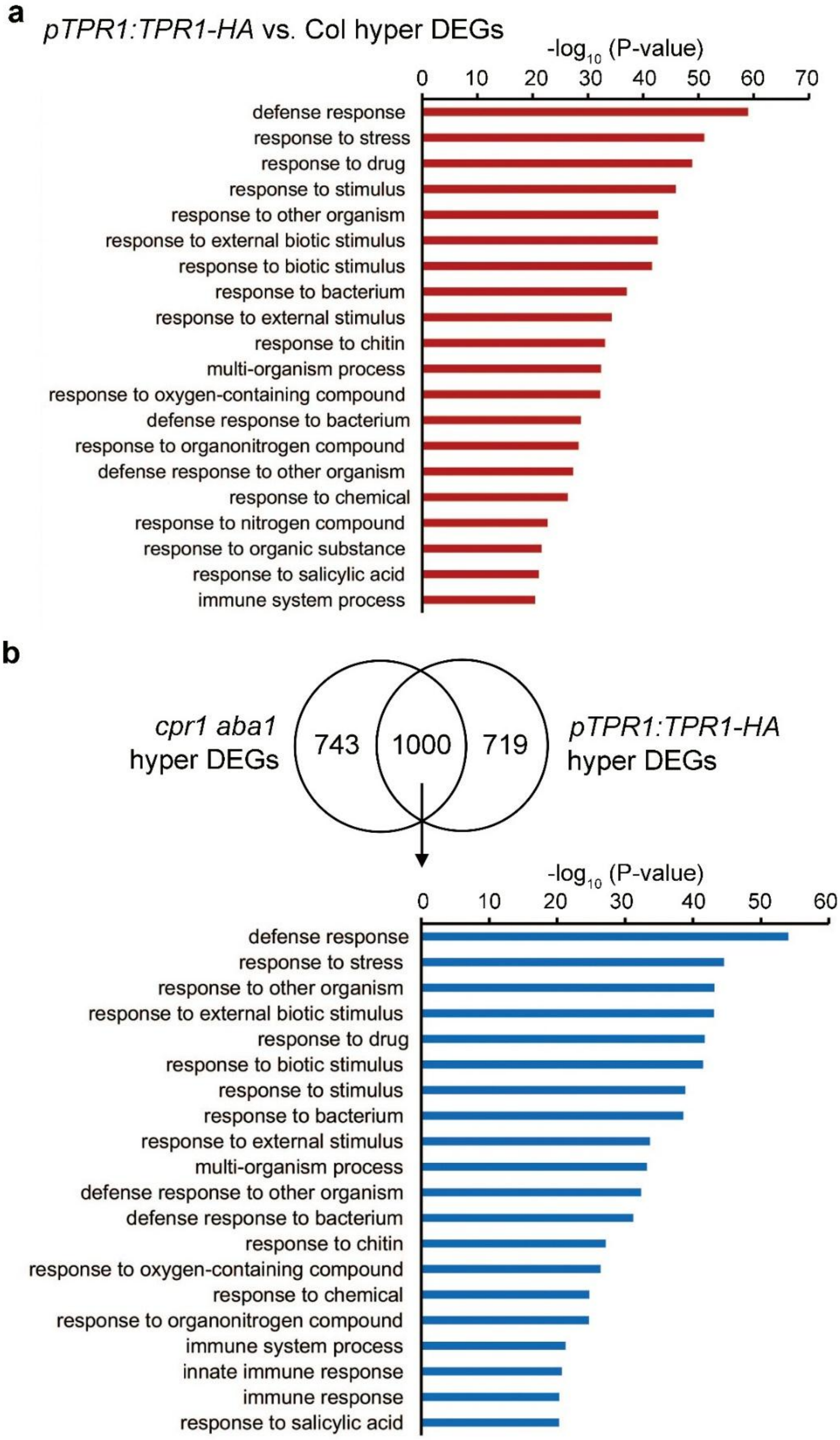

Supplementary Figure 5. Gene Ontology (GO) terms enriched in differentially expressed genes (DEGs). **a** GO enrichment analysis of 1,719 up-regulated genes in *pTPR1:TPR1-HA* as compared to Col with fold-change > 2.0, P < 0.01 and RPM > 10 in either genotype (average of three replicates). The top 20 GO terms are presented.

The 1,719 genes are listed in Supplementary Data 6. **b** GO enrichment analysis of the overlapping 1,000 up-regulated genes in *cpr1 aba1* and *pTPR1:TPR1-HA* as compared to Col with fold-change > 2.0, P < 0.01 and RPM > 10 in either genotype (average of three replicates). The top 20 GO terms are presented. The numbers and overlap in up-regulated genes in *cpr1 aba1* and *pTPR1:TPR1-HA* are shown in the Venn diagram. The 1,000 genes are listed in Supplementary Data 7.

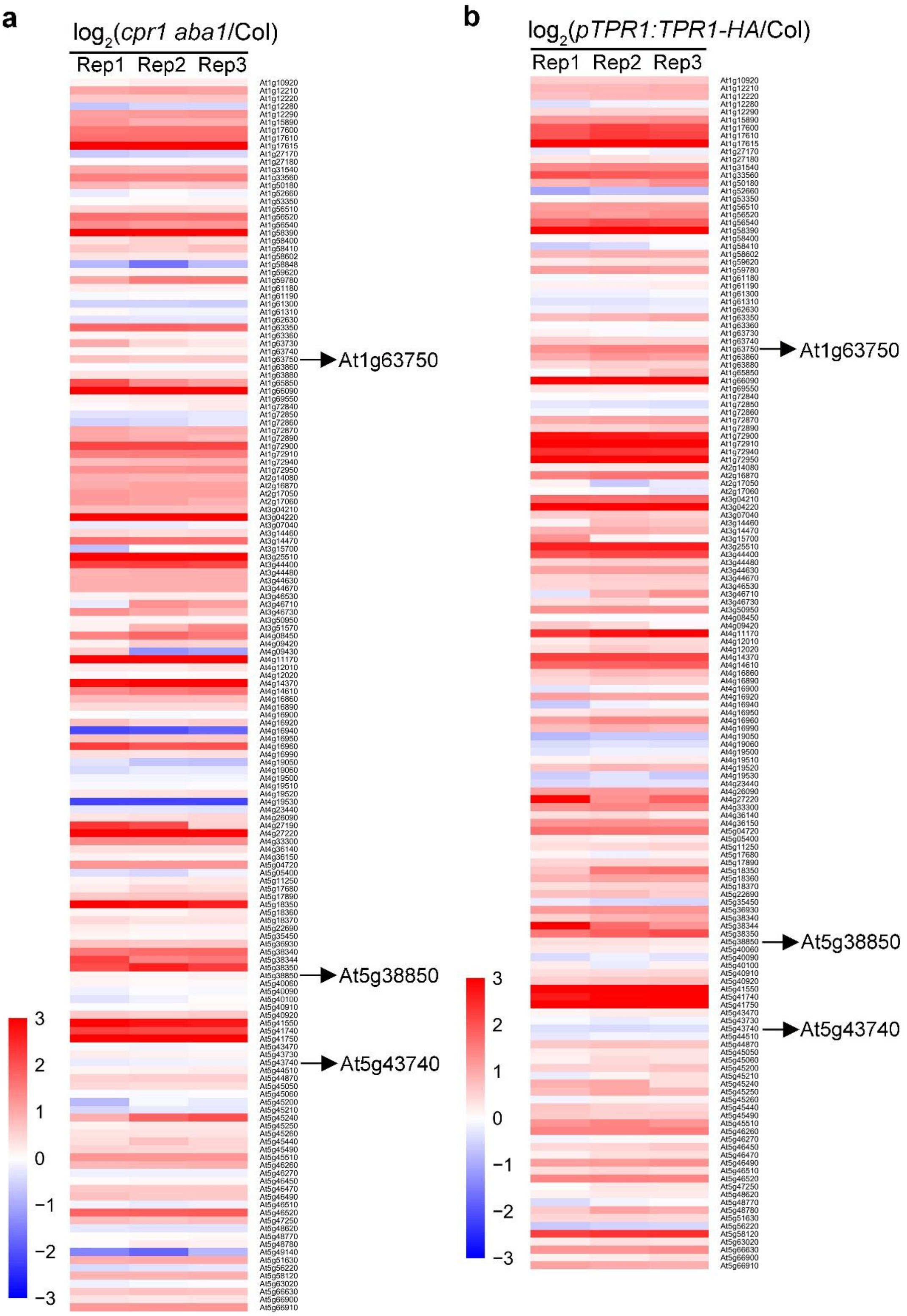

Supplementary Figure 6. Most *R* genes were up-regulated in *cpr1 aba1* and *proTPR1:TPR1-HA* relative to Col. **a** Analysis of *R* gene expression in wild type (Col) and *cpr1 aba1* as determined by RNA-seq. Three biological replicates of RNA-seq were performed for each genotype. 156 *R* genes with RPM > 10 in either genotype (average

of three replicates) were included in the analysis (Supplementary Data 8). The arrows mark the three source *R* genes (At5g43740, At5g38850 and At1g63750) that could generate phasiRNAs. **b** Analysis of *R* gene expression in wild type (Col) and *pTPR1:TPR1-HA* as determined by RNA-seq. Three biological replicates of RNA-seq were performed for each genotype. 151 *R* genes with RPM > 10 in either genotype (average of three replicates) were included in the analysis (Supplementary Data 10). The arrows mark the three source *R* genes (At5g43740, At5g38850 and At1g63750) that could generate phasiRNAs.

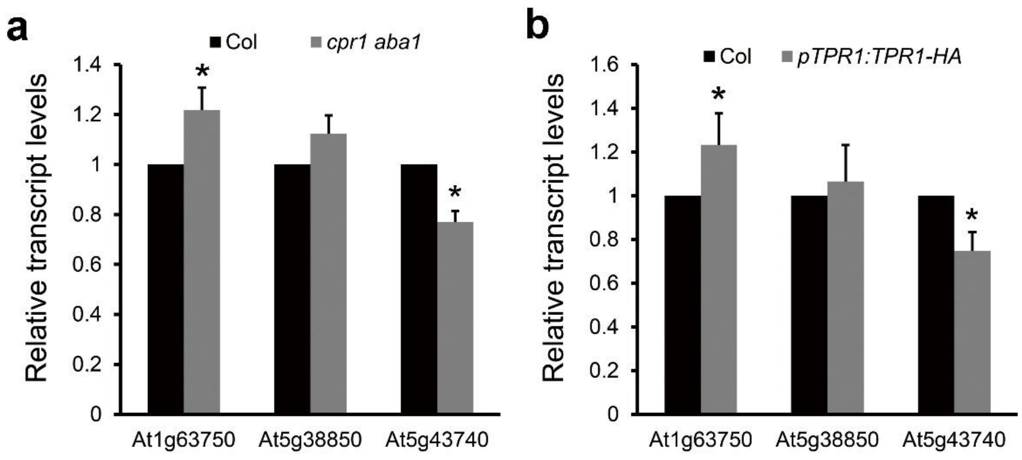

Supplementary Figure 7. The transcript levels of three *R* genes that could generate phasiRNAs. **a-b** The transcript levels of At1g63750, At5g38850 and At5g43740 were determined by real-time RT-PCR in 15-day-old seedlings of *cpr1 aba1* (**a**) and *pTPR1:TPR1-HA* (**b**), and compared to Col. Error bars represent standard deviations calculated from three biological replicates (*Student's *t* test: P < 0.05). Source data are provided as a Source Data file.

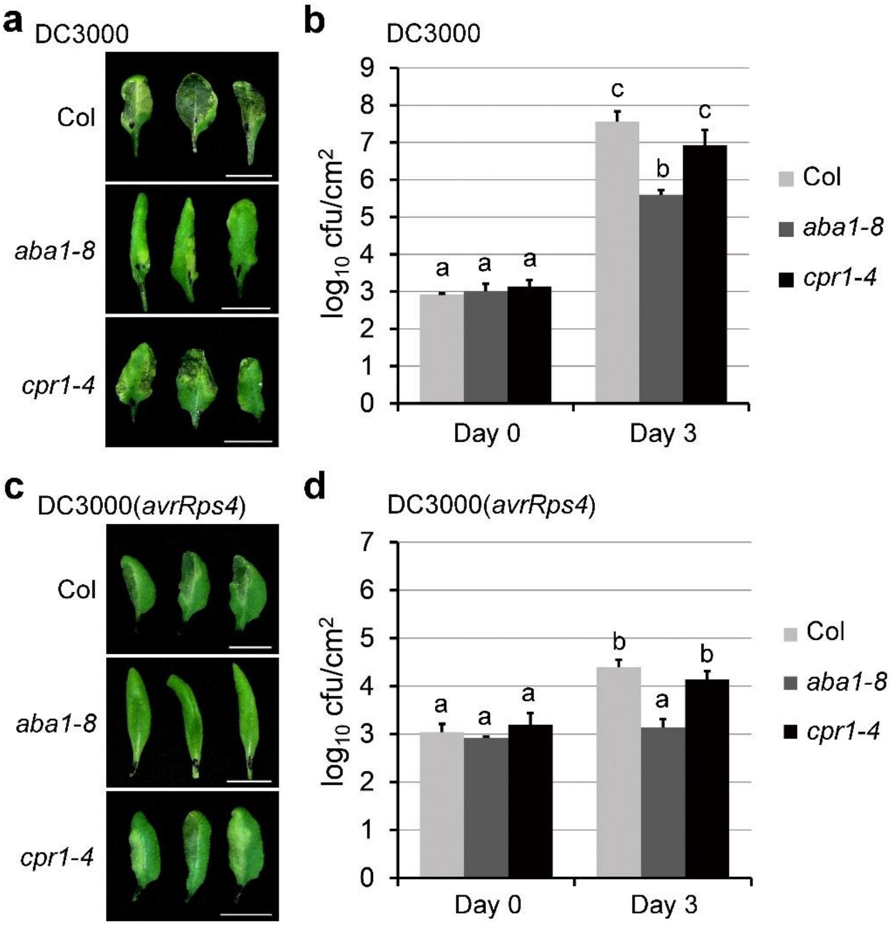

Supplementary Figure 8. Response of *aba1-8* and *cpr1-4* to *Pst* DC3000 and *Pst* DC3000(*avrRps4*). Rosette leaves of five-week-old plants of Col, *aba1-8* and *cpr1-4* were infiltrated with bacterial suspensions of *Pst* DC3000 or *Pst* DC3000(*avrRps4*) at OD_{600} = 0.001 using a needleless syringe. **a** Pictures of Arabidopsis leaves inoculated with *Pst* DC3000 were taken at 3 days post inoculation. Bar = 1 cm. **b** Bacterial populations of *Pst* DC3000 in Col, *aba1-8* and *cpr1-4* at day 0 and day 3 post inoculation. Error bars represent standard deviations calculated from three biological replicates. One-way ANOVA and post-hoc Tukey testing were used for statistical comparison. **c** Pictures of Arabidopsis leaves inoculated with *Pst* DC3000(*avrRps4*) were taken at 3 days post inoculation. Bar = 1 cm. **d** Bacterial populations of *Pst* DC3000(*avrRps4*) in Col, *aba1-8* and *cpr1-4* at day 0 and day 3 post inoculation. Error bars represent standard deviations calculated from three biological replicates. One-way ANOVA and post-hoc Tukey testing were used for statistical comparison. Source data are provided as a Source Data file.

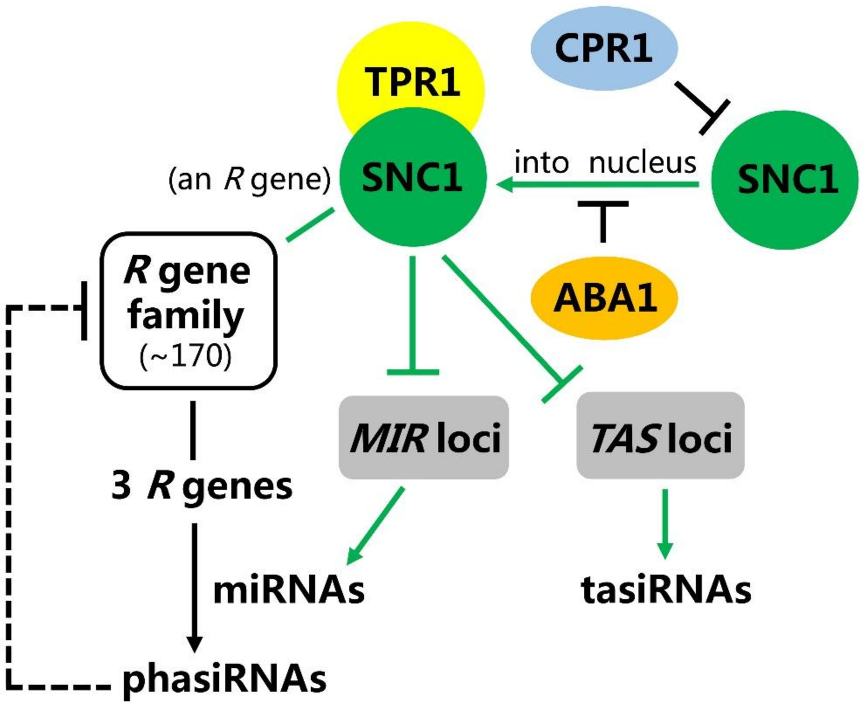

Supplementary Figure 9. A model of global *R* gene expression regulation. In the absence of pathogen infection, a small RNA cascade silences the expression of the *R* gene family. In this cascade, three *R* genes are targeted by miRNAs for phasiRNA production from their transcripts. The phasiRNAs likely in turn repress most *R* genes. In this study, we show that the over expression and nuclear localization of the R protein SNC1 (due to mutations of both CPR1 and ABA1) decreases the transcription of *MIR* loci and *TAS* loci, probably in concert with its interacting partner TPR1, resulting in reduced levels of miRNAs and tasiRNAs. SNC1 also represses the biogenesis of phasiRNAs from three source *R* genes, probably through its effects on the trigger miRNAs, leading to a release of the repression of *R* gene expression.

Supplementary Table 1. Primers used for genotyping

Oligonucleotide	Sequence (5'-3')
cpr1-4 genotyping-F	AACTCTAGTTCGTTGCCG
cpr1-4 genotyping-R	GAACCATCCTCACAACTT
aba1-8 genotyping-F	CTCAGGGAAACTCACCAAGT
aba1-8 genotyping-R	AGATGGGTTCAACTCCGTT
snc1-11-LP	TCGGCATAACATCGTAAGAGC
snc1-11-RP	CAAGCTTTCGTGGAGAAGATG
LBb1.3	ATTTTGCCGATTTCGGAAC
cpr1-3-LP	TTTCGTAAATTTTTACACAAAATCG
cpr1-3-RP	TGTGAGTAGCCTTGTCTTGGG
aba1-7-LP	GATGTTGGTGGTGGAAAAATG
aba1-7-RP	ACGTTCAAGAGCATCGTCATC

Supplementary Table 2. Primers used for plasmid construction

Oligonucleotide	Sequence (5'-3')
proCPR1-KpnI-F	CACGGTACCTCTATATATGTGTCTAAC
CPR1-PstI-R	GTTCTGCAGTAAGACCAGCTTGAATCC
proABA1-KpnI-F	CACGGTACCGGCTTAAACCTAACTCTA
ABA1-SpeI-R	GTTACTAGTAGCTGTCTGAAGTAATTTAT
SNC1-KpnI-F	GGAGGTACCATGATGGATACATCCAAAG
SNC1-PstI-R	GCCCTGCAGGTTACCAGAAACAGGAAA
PstI-NLS-BamHI-GFP-F	AATCTGCAGATGGCGCCAAAAAAGAAGAGAAAGGTCGG<br>ATCCATGGTGAGCAAGGGCGAGG
GFP-SacI-R	CCTGAGCTCTTACTTGTACAGCTCGTC

Supplementary Table 3. Primers used for quantitative RT-PCR

Oligonucleotide	Sequence (5'-3')
qPCR-pri-miR156a-F	GAAAGAGTTGGGACAAGAGAAACG
qPCR-pri-miR156a-R	AGAGAACGAAGACAGGCCAAAGA
qPCR-pri-miR158a-F	GACTTTAGATTAGAGGGC
qPCR-pri-miR159a-R	GAAACATTTACACGCTCG
qPCR-pri-miR159a-F	TCTTTACAGTTTGCTTATGTCAGATCCA
qPCR-pri-miR159a-R	ACCCTGCTCAACTCATGTTTGAA
qPCR-pri-miR159b-F	TGCTTGGATCTCTAATGCTGTTCA
qPCR-pri-miR159b-R	TCACCCTGCTAAACCCTCCA
qPCR-pri-miR164b-F	TGCGGAATTTTGTGATATAGATGTGT
qPCR-pri-miR164b-R	CTCGTCGTCATCGTCACCAA
qPCR-pri-miR166a-F	AGATATATATTCAGAAACCCTAG
qPCR-pri-miR166a-R	GGTTCATTCACTGGATCTGAAAC

qPCR-pri-miR167a-F	TGATCTGCTACGGTGAAGTCTATGG
qPCR-pri-miR167a-R	GAAACTGCGAACATGATCTAATCGA
qPCR-pri-miR167b-F	TCTTTGGTTAAGAGATGAATGTGGAA
qPCR-pri-miR167b-R	ATTTTTCTTTCAATCGGCATGTG
qPCR-pri-miR161-F	CTCGGTTTTTGACCAGTTT
qPCR-pri-miR161-R	AGGGTTGATTACTGTTTCC
qPCR-pri-miR173-F	GTACTTTCGCTTGCAGAG
qPCR-pri-miR173-R	TAGGGAGCAAGCTCTTTC
qPCR-pri-miR319b-F	ACGCACAGAGAGGAAGAT
qPCR-pri-miR319b-R	CCTCCACCAACTCAACAT
qPCR-pri-miR390b-F	CCTATGGATGTAAATTTCAGATTGAG
qPCR-pri-miR390b-R	CGAAGGAGGGAATGAAGTAGG
qPCR-pri-miR393b-F	GAGAGAGTTCTTCACAGCAA
qPCR-pri-miR393b-R	CATGATCCGGAAAAGTAAGC
qPCR-pri-miR394b-F	TCGACAGAAAGGAAATGAGTGA
qPCR-pri-miR394b-R	TCCTCTTACGAAACACACCGTAGATAC
qPCR-pri-miR396a-F	TGACCCTCTCTGTATTCT
qPCR-pri-miR396a-R	TCTGTCTGTATCTTCCCA
qPCR-TAS1A-F	CCTGTCTATTTGTCACCAGCC
qPCR-TAS1A-R	GAACGCTATGTTGGACTTAGGA
qPCR-TAS1B-F	CAGTCCCGTGCTCTCTGTAT
qPCR-TAS1B-R	GGTGAATGGTTAGATACCGATG
qPCR-TAS1C-F	ATGTGTCAGTTTCGTTCCTTCC
qPCR-TAS1C-R	CCACCGATAAATGGTCTATTCG
qPCR-TAS2-F	TAGGTTGGGTTTGGGAGTG
qPCR-TAS2-R	CATCATTCGCTTGGAGAGA
qPCR-TAS3A-F	CGAAGTTTCTCCAAGGCA
qPCR-TAS3A-R	CTCAGATAGGATAACACCGCT
qPCR-TAS3B-F	GGGAAGTGATTCAAGGCTT
qPCR-TAS3B-R	GCTCAGGTAAGATAGCGTCAT
qPCR-UBQ5-F	GGTGCTAAGAAGAGGAAGAA
qPCR-UBQ5-R	CTCCTTCTTTCTGGTAAACGT
qPCR-GUS-F	TCAGGAAGTGATGGAGCA
qPCR-GUS-R	AGAGCATTACGCTGCGAT
qPCR-SUL-F	GATCCAAAGATTGGTGGTGTTATG
qPCR-SUL-R	AACTTGCTCTCCTTTCTCAACTCT
qPCR-At1g63750-F	TCAAGGCAAGGGGATAAC
qPCR-At1g63750-R	GCCTTTTTGCGCTTCATG
qPCR-At5g38850-F	GCTCTCATACAAGCGATT
qPCR-At5g38850-R	CCTCTCCTCCTTAGTTTT
qPCR-At5g43740-F	AAACTGGTGGAAATGGCG
qPCR-At5g43740-R	CTTTCCCATTCCTTGTCAG

Supplementary Table 4. Northern blot probes

**Oligonucleotide**	**Sequence (5'-3')**
amiR-SUL probe-Biotin	AGGGATTTCCGTGACACTTAA
miR156 probe-Biotin	GTGCTCACTCTCTTCTGTCA
miR158 probe-Biotin	TGCTTTGTCTACATTTGGGA
miR159 probe-Biotin	TAGAGCTCCCTTCAATCCAAA
miR161 probe-Biotin	TAGTCACTTTCAATGCATTGA
miR164 probe-Biotin	TGCACGTGCCCTGCTTCTCCA
miR167 probe-Biotin	TAGATCATGCTGGCAGCTTCA
miR173 probe-Biotin	GTGATTTCTCTCTGTAAGCGA
miR319 probe-Biotin	GGGAGCTCCCTTCAGTCCAA
miR390 probe-Biotin	GGCGCTATCCCTCCTGAGCTT
miR393 probe-Biotin	GATCAATGCGATCCCTTTGGA
miR396 probe-Biotin	CAGTTCAAGAAAGCTGTGGAA
miR828 probe-Biotin	TGGAATACTCATTTAAGCAAGA
miR472 probe-Biotin	GGTATGGGCGGAGTAGGAAAAA
miR393 probe-Biotin	GATCAATGCGATCCCTTTGGA
miR825* probe-Biotin	GCTTCTTCGAGCTGGTGCTTGA
Tas1-siR255 probe-Biotin	TACGCTATGTTGGACTTAGAA
Tas2-siR1511 probe-Biotin	AAGTATCATCATTCGCTTGGA
Tas3-5'D8 probe-Biotin	AAAGGCCTTACAAGGTCAAGA
U6 probe-Biotin	AGGGGCCATGCTAATCTTCTCTG
