# Supplementary material for: The disease resistance protein SNC1 represses the biogenesis of microRNAs and phased siRNAs
Source: Nat Commun. 2018 Nov 29;9:5080. doi: 10.1038/s41467-018-07516-z (PMC6265325; doi:10.1038/s41467-018-07516-z)
Supplement: Supplementary file 2 — Description of Additional Supplementary Files [file 41467_2018_7516_MOESM2_ESM.docx]

**Description of Additional Supplementary Files**

**File Name**: Supplementary Data 1

**Description**: Levels of miRNAs in amiR-SUL sup-B65 and amiR-SUL as determined by small RNA sequencing

**File Name**: Supplementary Data 2

**Description**: Levels of miRNAs in amiR-SUL snc1-1 and amiR-SUL as determined by small RNA sequencing

**File Name**: Supplementary Data 3

**Description**: PhasiRNA loci analyzed in this study

**File Name**: Supplementary Data 4

**Description**: GO terms for up-regulated genes in cpr1 aba1 relative to Col

**File Name**: Supplementary Data 5

**Description**: GO terms for down-regulated genes in cpr1 aba1 relative to Col

**File Name**: Supplementary Data 6

**Description**: GO terms for up-regulated genes in pTPR1:TPR1-HA relative to Col

**File Name**: Supplementary Data 7

**Description**: GO terms for up-regulated genes in both cpr1 aba1 and pTPR1:TPR1-HA relative to Col

**File Name**: Supplementary Data 8

**Description**: Expression levels of 165 R genes in Col and cpr1 aba1 as determined by RNA-seq

**File Name**: Supplementary Data 9

**Description**: Transcript levels of 74 R genes that are differentially expressed between Col and cpr1 aba1

**File Name**: Supplementary Data 10

**Description**: Expression levels of 166 R genes in RNA-seq of Col and pTPR1:TPR1-HA

**File Name**: Supplementary Data 11

**Description**: Transcript levels of 70 R genes that are differentially expressed between Col and pTPR1:TPR1-HA

**File Name**: Supplementary Data 12

**Description**: A list of R genes that can be potentially targeted by phasiRNAs generated by the three source R genes

**File Name**: Supplementary Data 13

**Description**: Source Data
