# Supplementary material for: The disease resistance protein SNC1 represses the biogenesis of microRNAs and phased siRNAs
Source: Nat Commun. 2018 Nov 29;9:5080. doi: 10.1038/s41467-018-07516-z (PMC6265325; doi:10.1038/s41467-018-07516-z)
Supplement: Supplementary file 16 — Reporting Summary [file 41467_2018_7516_MOESM16_ESM.pdf]

nature research

Corresponding author(s): Xuemei Chen

Reporting Summary

Nature Research wishes to improve the reproducibility of the work that we publish. This form provides structure for consistency and transparency in reporting. For further information on Nature Research policies, see Authors & Referees and the Editorial Policy Checklist.

Statistical parameters

When statistical analyses are reported, confirm that the following items are present in the relevant location (e.g. figure legend, table legend, main text, or Methods section).

n/a	Confirmed	
☐	☒	The exact sample size (*n*) for each experimental group/condition, given as a discrete number and unit of measurement
☐	☒	An indication of whether measurements were taken from distinct samples or whether the same sample was measured repeatedly
☐	☒	The statistical test(s) used AND whether they are one- or two-sided<br>*Only common tests should be described solely by name; describe more complex techniques in the Methods section.*
☒	☐	A description of all covariates tested
☐	☒	A description of any assumptions or corrections, such as tests of normality and adjustment for multiple comparisons
☐	☒	A full description of the statistics including central tendency (e.g. means) or other basic estimates (e.g. regression coefficient) AND variation (e.g. standard deviation) or associated estimates of uncertainty (e.g. confidence intervals)
☐	☒	For null hypothesis testing, the test statistic (e.g. *F*, *t*, *r*) with confidence intervals, effect sizes, degrees of freedom and *P* value noted<br>*Give P values as exact values whenever suitable.*
☒	☐	For Bayesian analysis, information on the choice of priors and Markov chain Monte Carlo settings
☒	☐	For hierarchical and complex designs, identification of the appropriate level for tests and full reporting of outcomes
☒	☐	Estimates of effect sizes (e.g. Cohen's *d*, Pearson's *r*), indicating how they were calculated
☐	☒	Clearly defined error bars<br>*State explicitly what error bars represent (e.g. SD, SE, CI)*

Our web collection on statistics for biologists may be useful.

Software and code

Policy information about availability of computer code

Data collection

```
#!/usr/bin/perl
use strict;
use warnings;
use File::Basename;

############################################################################
#
#  This script is designed to identify candidate phasiRNA
#  loci based on small RNA sequencing data.
#
#  Author: Xuan Ma
# (Institute of Genetics and Devlopmental Biology, CAS)
#
#  If there exits any bugs, please report to xuanma@genetics.ac.cn
#
############################################################################


if (@ARGV < 2) {
```

```
 print "Usage: perl $0 sRNA.sam cdna.fa\n";
 exit;
}


my $sam_file = shift;
my (%data1, %data2, %phase_res);
print STDERR "Reading SAM ...\n";
open my $sam, '<', $sam_file or die $!;
while (<$sam>) {
 next if /^\s*$/;
 next if /^(\@HD|\@SQ|\@PG)/;
 next if /SAM header/;
 next if /\#/;
 chomp;
 my ($readID, $flag, $gene, $pos, $MAPQ, $len, $line) = split;
 next if !($len eq '21M' or $len eq '22M');  #21-22nt siRNA
 if ($flag == 0)     {$data1{$gene}{$pos}{0} ++;}  #forward strand
 elsif ($flag == 16) {$data2{$gene}{$pos}{16} ++;} #reverse strand
}
close $sam;


print STDERR "Combining strands ...\n";
for my $gene (keys %data2) {
 for my $pos (keys %{$data2{$gene}}) {
  # offset 2nt
  if (exists $data1{$gene}{$pos + 2}{0}) {
   $data1{$gene}{$pos + 2}{0} += $data2{$gene}{$pos}{16};
  } else {
   $data1{$gene}{$pos + 2}{0} = $data2{$gene}{$pos}{16};
  }
 }
}
%data2 = ();


print STDERR "Reading genome ...\n";
my $gene_fasta_file = shift;
my %len;
$/ ='>';
open my $gene_file, '<', $gene_fasta_file or die $!;
while (<$gene_file>) {
	chomp;
	my ($id, $seq) = /(.+?)\n(.+)/s or next;
  $id = $1 if $id =~ /(.+?)\s/; # remove info behind the 1st space
	$seq =~ s/\n//g;
	$len{$id} = length($seq);
}
close $gene_file;
$/ = "\n";


for my $gene (keys %data1) {
 for my $pos (1..$len{$gene}) {
  $data1{$gene}{$pos}{0} ||= 0;
 }
}


print STDERR "Phase scoring...\n";
for my $gene (keys %data1) {
 for my $pos (sort {$a <=> $b} keys %{$data1{$gene}}) {

  my ($n, $phased, $unphased, $phasing_score) = (0, 0, 0, 0);

  for my $cycle (0..9) { # 10 cycles B.Meyers lab, RNA, 2009
   my $new_pos = $pos + 21 * $cycle; # phased position
   if (exists $data1{$gene}{$new_pos}{0}  and
    $data1{$gene}{$new_pos}{0} > 0) {
    $n ++;  # num of pos plus 1
    $phased += $data1{$gene}{$new_pos}{0}; # phased cumulation
   }

   for my $unphased_nt_per_cycle (1..20) {
```

```
   $new_pos ++; # unphased positions
   if (exists $data1{$gene}{$new_pos}{0} and
    $data1{$gene}{$new_pos}{0} > 0) {
    $n ++; # num of pos plus 1
    $unphased += $data1{$gene}{$new_pos}{0};# unphased cumulation
   }
  }
 }

 if ($n < 3) {
  $phasing_score = 0;
 } else {
  $phasing_score = ($n - 2) * log (1 + 10 * $phased / ($unphased + 1)); # B.Meyers lab 2009
 }
 $phase_res{$gene}{$pos} = $phasing_score;
 }
}


print STDERR "Outputing ...\n";
(my $sam_file_base = basename $sam_file) =~ s/(.+)\.sam/$1/;
open my $phase_res_out, '>', "$sam_file_base\_phase_score_siRNA.txt";
for my $gene (keys %phase_res) {
 for my $pos (1..$len{$gene}) {
  print $phase_res_out "$gene\t$pos\t$phase_res{$gene}{$pos}\t$data1{$gene}{$pos}{0}\n";
 }
}
close $phase_res_out;

__END__

Notes:

1) After running this script, an output file named sRNA_phase_score_siRNA.txt is generated.
   The first column is gene ID, the second column is position, the third column is phasing score,
   and the last column is normalized sRNA counts.

2) References:
   De Paoli, et al. RNA, 2009, 15:1965-1970
   Miya D Howell, et al. Plant Cell, 2007, 19:926-942

#!/usr/bin/perl
use strict;
use Getopt::Long;
use Pod::Usage qw(pod2usage);

###Help information

=pod

=head1 NAME

PIHSDA - PLANT INTEGRATED HIGH-THROUGHPUT SEQUENCING DATA ANALYSIS

=head1 SYNOPSIS

USAGE:

 pihsda -m MODE [OPTION] [NAME1] [#1] [SEQ1] [SEQ2] .. [SEQ#1] [NAME2] [#2] [SEQ1] [SEQ2] .. [SEQ#2] ..

OPTIONS:

 -h,--help  print this help message

 -m,--mode  Specify a mode. Select from "srna", "mrna", "tt", "deg", and "clip"

 -a,--adapter  The sequence of 3' adapter. Default: AGATCGGAAGAGC or 1, TGGAATTCTCGGG or 2 is another frequently used adapter
sequence

 -o,--output  The directory for output files. Default: ./out

 -t,--thread  The number of processors used in bowtie mapping. Default: 4

 -g,--genome  The reference genome.
   ath for Arabidopsis thaliana TAIR10
```

```
    gma for Glycine max v109
    osa for Oryza sativa MSUv7..
    Default: ath

 -f,--foldchange  The threshold of fold change in DS analysis. Default: 1.5

 -p,--pvalue  The threshold of P value in DS analysis. Default: 0.01

 --no-mapping  Only perfome the statistic analysis

 --mapping-only  Only to do the mapping

SRNA MODE OPTIONS

 --mmap   The method used in mapping.
    n for unique, r for random, u for unique-seeded guide, and f for fractional-seeded guide.
    Default: u

 -n,--norm  The method used in read count normalization.
    t for total, r for rRNA, and b for both.
    Default: b

MRNA MODE OPTIONS

 --mismatch  The number of allowed mismatches used in mapping. Default: 1

 --multihits  The number of allowed multihits loci used in mapping. Default: 1

TRUNCATION AND TAILING MODE OPTIONS

DEGRADOME MODE OPTIONS

CLIP-SEQ MODE OPTIONS

 --premRNA  Use premRNA length cutoff

=head1 DESCRIPTION

To successully run this script, please ensure the correct installation of R packages 'DESeq2' and 'pheatmap', and avoid naming the samples starting with digits.

=head1 AUTHORS

You, Chenjiang

1/2/2017

=head1 SEE ALSO

See README.md for more details.

=cut

####Start


print STDERR "\nWelcome to use the Plant sRNA Analysis Pipeline!\nVersion 2.2\tLast update: 05/10/2017\n";
my $time = localtime;

####Reading parameters

die "\ntype \"-h\" or \"--help\" for more information" if ($#ARGV==-1);
print STDERR "\nCommand: @ARGV\n\n";

my $genome = "ath";
my $output = "out";
my $adapter = "";
my $core = 4;
my $foldchange = 1.5;
my $pvalue = 0.01;
my $mmap = "u";
my $mnorm = "b";
my $nophasi = 0;
my $nomapping = 0;
my $only = 0;
```

```
my $help = "";
my $mode = "";
my $mis = 1;
my $multi = 1;
my $pre="";

GetOptions(
 'm|mode=s' => \$mode,
 'h|help!' => \$help,
 'g|genome=s' => \$genome,
 'o|out=s' => \$output,
 'a|adapter=s' => \$adapter,
 't|thread=i' => \$core,
 'f|foldchange=s' => \$foldchange,
 'p|pvalue=s' => \$pvalue,
 'mmap=s' => \$mmap,
 'n|norm=s' => \$mnorm,
 'mismatch=i' => \$mis,
 'multihits=i' => \$multi,
 'no-mapping!' => \$nomapping,
 'mapping-only!' => \$only,
 'no-phasi!' => \$nophasi,
 'premRNA!' => \$pre,)
or die("Error in command line arguments\n");
pod2usage(-verbose => 2, -exitval => 2) if ($help);

open STDOUT, ">>log.txt" or die $!;
print STDOUT "Start: $time\n";
print STDOUT "\nCommand: @ARGV\n\n";

$0 =~ s/\/pihsda$//;
my $prefix = $0;

die "Please specify a mode!" if($mode !~ /srna|tt|mrna|deg|clip/);

die "Parameters confilicted: --no-mapping and --mapping-only!" if($only + $nomapping > 1);

if(!$only){
 if(-e $output){
  die "$output already exists! Please provide another output folder!";
 }
}

die "This genome is not supported!\ntype \"-h\" for more information" if($genome !~ /ath|osa|gma|zma/);

$adapter = "AGATCGGAAGAGC" if($adapter == 1);
$adapter = "TGGAATTCTCGGG" if($adapter == 2);
$adapter = "CCCAGATCGGAAG" if($mode eq "clip");
if($mode ne "mrna" && $adapter eq ""){
 die "Please specify the adapter sequence for cutadapt!\n";
}
$pre = "--premRNA" if($pre);
die "Please provide the correct method for read count normalization!\ntype \"-h\" for more information" if($mnorm !~ /[brt]/);

if($#ARGV < 2){
 print STDERR "Please provide enough parameters\n";
 print STDERR "type \"-h\" for more information\n";
 exit;
}

####Check dependent software

my $cutadapt = `cutadapt --version`;
my $samtools = `samtools --version`;
my $bowtie = `bowtie --version`;
my $shortstack = `ShortStack -v`;
my $bedtools = `bedtools --version`;
my $R = `R --version`;
my $hisat = `hisat2 --version`;
my $clipper = `clipper -h`;

if($cutadapt ne ""){
 print STDERR "cutadapt version $cutadapt";
}else{
 die "Please install cutadapt!\n";
```

```
}
if($samtools =~ /samtools (\d)\.(\d)/){
 if($1==1){
  print STDERR "samtools version $1\.$2\n";
 }else{
  die "Please install samtools v1.x!\n";
 }
}else{
 die "Please install samtools v1.x!\n";
}
if($bowtie =~ /bowtie version (\d\.\d\.\d+)/){
 print STDERR "bowtie version $1\n";
}else{
 die "Please install bowtie!\n";
}
if($shortstack =~ /version 3/){
 print STDERR "$shortstack";
}else{
 die "Please install ShortStack!\n";
}
if($bedtools ne ""){
 print STDERR "$bedtools";
}else{
 die "Please install bedtools!\n";
}
if($R =~ /R version (\d\.\d\.\d+)/){
 print STDERR "R version $1\n";
}else{
 die "Please install R\n";
}
if($hisat =~ /hisat2.+version (2.+)/){
 print STDERR "HISAT version $1\n";
}else{
 die "Please install HISAT2!";
}
if($clipper =~ /^Usage:/){
 print STDERR "CLIPper installed\n";
}else{
 die "Please install CLIPper!";
}

####Pre-processing

my (%geneinfo, %exon);
system ("gffread -w exons.fa -g ".$prefix."/reference/".$genome."_chr_all.fasta ".$prefix."/reference/".$genome."_genes.gff");
open EXON, "exons.fa" or die $!;
my ($gene, $tran);
while(my $exo = <EXON>){
 chomp $exo;
 if($exo =~ />(AT\w+)\.(\d+)/){
  $gene = $1;
  $tran = $2;
 }elsif($exo=~ />(AT\w+)/){
  $gene = $1;
  $tran = 1;
 }else{
  $exon{$gene}{$tran} .= $exo;
 }
}
close EXON;
unlink "exons.fa";
foreach $gene (sort keys %exon){
 my $long = 0;
 foreach $tran (keys %{$exon{$gene}}){
  if(length($exon{$gene}{$tran}) > $long){
   $long = length $exon{$gene}{$tran};
  }
 }
 $geneinfo{$gene}{"long"} = $long;
}

####Run

if($mode eq "srna"){
```

```
####Pre-processing

my %length =();
if(!$nomapping){

 die "Please provide the correct method for mapping!\ntype \"-h\" for more information" if($mmap !~ /[nurf]/);

 open FAI, "$prefix/reference/$genome"."_chr_all.fasta.fai" or die $!;
 while (my $fai = <FAI>){
  chomp $fai;
  my @row = split /\t/, $fai;
  $length{$row[0]} = int($row[1]/100);
 }
 close FAI;
 open GENE, "$prefix/reference/$genome"."_genes.gff" or die $!;
 open TE, "$prefix/reference/$genome"."_transposons.gff" or die $!;
 open TMP1, ">gene.gff" or die $!;
 open TMP2, ">promoter.gff" or die $!;
 while(my $aa = <GENE>){
  chomp $aa;
  my @row = split /\t/, $aa;
  if($row[2] =~ /gene/){
   if($row[8] =~ /ID=(\w+);/o){
    my $name = $1;
    if($row[8] =~ /Note=transposable_element_gene;/){
     $row[8] = $name."_TEG";
    }else{
     $row[8] = $name;
    }
    my $row = join "\t", @row;
    print TMP1 "$row\n";
    $row[8] .= "_promoter";
    if($row[6] eq "+"){
     $row[4] = $row[3] - 1;
     if($row[3] > 1000){
      $row[3] = $row[3] - 1000;
     }else{
      $row[3] = 1;
     }
    }else{
     $row[3] = $row[4] + 1;
     $row[4] = $row[4] + 1000;
    }
    $row = join "\t", @row;
    print TMP2 "$row\n";
   }
  }
 }
 close GENE;
 close TMP1;
 close TMP2;
 open TMP, ">te.gff" or die $!;
 while(my $bb = <TE>){
  chomp $bb;
  my @row = split /\t/, $bb;
  if($row[8] =~ /ID=(\w+);N/o){
   $row[8] = $1;
   my $row = join "\t", @row;
   print TMP "$row\n";
  }
 }
 close TMP;
 close TE;
}

####Mapping start

my @out;
while(@ARGV){
 my $sample = shift @ARGV;
 push @out, $sample;
 my $num = shift @ARGV;
 die "Please provide enough biological replicates\ntype \"-h\" for more information" if($num < 1);
 push @out, $num;
 if(!$nomapping){
```

```
  system ("awk '{print \$9}' ".$prefix."/reference/".$genome."_miRNA_miRNA_star.gff >1.new");
  open TMP, ">1.bin" or die $!;
  foreach my $chr (sort keys %length){
   for(my $bi=0;$bi<=$length{$chr};$bi++){
    print TMP "$chr\_$bi\n";
   }
  }
  close TMP;
  for(my $i=1;$i<=$num;$i++){
   my $file = shift @ARGV;
   my $tag = $sample."_".$i;
   print STDERR "Mapping $tag...\n";
   print STDOUT "Mapping $tag...\n";
   &trimming($file, $tag);
   if($genome eq "ath"){
    system ("awk -F \"\t\" \'{print \$3}\' ".$tag.".rRNA.out \| sort \|uniq -c \|sort -rn \| awk \'BEGIN{x=0}{if(\$2==
\"CP002686.1/14199748-14203300\"\|\|\$2==\"X52320.1/492-4032\"\|\|\$2==\"AC006837.16/4777-8329\"\|\|\$2==
\"X52322.1/6196-9736\"\|\|\$2==\"CP002685.1/5777-9329\"){x=x+\$1}}END{print \"rRNA\t\"x}\' > ".$tag.".nf");
   }
   #Step 3 Map filtered reads to the genome
   system ("ShortStack --outdir ShortStack_".$tag." --align_only --bowtie_m 1000 --ranmax 50 --mmap ".$mmap." --mismatches 0 --
bowtie_cores ".$core." --nohp --readfile ".$tag."-rRNA-free.fastq --genomefile ".$prefix."/reference/".$genome."_chr_all.fasta
2>>log.txt");
   system ("mv ".$tag."-rRNA-free.fastq ".$tag.".rRNA-free.fastq");
   print STDERR "Alignment  Completed!\n";
   system ("samtools view -h ShortStack_".$tag."/".$tag."-rRNA-free.bam > ".$tag.".rRNA-free");
   system ("awk '{if(\$0~/^@/) print > (FILENAME\".unmapped.sam\"); if(\$10!=\"*\" && \$3!=\"*\") print > (FILENAME\".sam\"); if(\$10!
=\"*\" && \$3==\"*\") print > (FILENAME\".unmapped.sam\")}' ".$tag.".rRNA-free");
   system ("samtools view -Sb ".$tag.".rRNA-free.unmapped.sam > ".$tag.".rRNA-free.unmapped.bam");
   system ("samtools view -Sb ".$tag.".rRNA-free.sam > ".$tag.".rRNA-free.bam");
   unlink ($tag.".rRNA-free", $tag.".rRNA-free.unmapped.sam", $tag.".rRNA-free.sam");
   print STDERR "BAM to BED...\n";
   system ("bamToBed -bed12 -i ".$tag.".rRNA-free.bam > ".$tag.".rRNA-free.bed");
   #Step 4 Generate bed file for each smRNA length (18-26nt)
   print STDERR "Generating individual files...\r";
   system ("awk -F \"\t\" '{a=substr(FILENAME,1,length(FILENAME)-3); if(\$11==18) print >> (a\"18.bed\"); else if(\$11==19) print >> (a
\"19.bed\"); else if(\$11==20) print >> (a\"20.bed\"); else if(\$11==21) print >> (a\"21.bed\"); else if(\$11==22) print >> (a\"22.bed\");
else if(\$11==23) print >> (a\"23.bed\"); else if(\$11==24) print >> (a\"24.bed\"); else if(\$11==25) print >> (a\"25.bed\"); else if(\
$11==26) print >> (a\"26.bed\"); close((a\"18.bed\")); close((a\"19.bed\")); close((a\"20.bed\")); close((a\"21.bed\")); close((a\"22.bed
\")); close((a\"23.bed\")); close((a\"24.bed\")); close((a\"25.bed\")); close((a\"26.bed\"))}' ".$tag.".rRNA-free.bed");
   #Step 5 Determine read distribution by length
   system ("awk '!a[\$4]++' ".$tag.".rRNA-free.bed \| awk '{print \$11}' \| sort \| uniq -c \| awk '{OFS=\"\t\"; print \$2, \$1}' > ".
$tag.".rRNA-free.len_dist.txt");
   system ("awk '{n+=\$2}END{print \"total\t\"n}' ".$tag.".rRNA-free.len_dist.txt >> ".$tag.".nf");
   print STDERR "Lenth distribution summary done!\n";
   #Step 6 Calculating the abundance of miRNA and bin
   print STDERR "Counting start...\r";
   opendir my $dir, "." or die $!;
   my @dir = grep {/$tag.*[0-9]\.bed/} readdir $dir;
   close $dir;
   open NF, "$tag.nf" or die $!;
   while (my $line = <NF>){
    chomp $line;
    my ($met, $rc) = split /\t/, $line;
    next if($met eq "rRNA" && $mnorm eq "t");
    next if($met eq "total" && $mnorm eq "r");
    foreach my $sbed (@dir){
     system ("bedtools intersect -a ".$prefix."/reference/".$genome."_miRNA_miRNA_star.gff -b ".$sbed." -wa -f 0.95 -c \|awk -v x=".$rc."
'{print \$10 * 1000000 / x}' > ".$sbed.".nnew");
     system ("bedtools intersect -a ".$prefix."/reference/".$genome."_miRNA_miRNA_star.gff -b ".$sbed." -wa -f 0.95 -c \|awk '{print \
$10}' > ".$sbed.".new");
     open BED, "$sbed" or die $!;
     my %hash = ();
     my %hash2 = ();
     my %hashp = ();
     my %hashn = ();
     while (my $bed = <BED>){
      chomp $bed;
      my @row = split /\t/, $bed;
         my $bin = int(($row[1]+1)/100);
         $hash{$row[0]}{$bin} ++;
      for(my $j=$row[1];$j<=$row[2];$j++){
             $hash2{$row[0]}{$j} ++;
       if($row[5] eq "+"){
        $hashp{$row[0]}{$j} ++;
```

```
   }else{
    $hashn{$row[0]}{$j} --;
   }
     }
}
close BED;
open TMP1, ">$sbed.bin" or die $!;
open TMP2, ">$sbed.nbin" or die $!;
foreach my $chr (sort keys %length){
    for(my $bi=0;$bi<=$length{$chr};$bi++){
  if(exists $hash{$chr}{$bi}){
      my $rpm = $hash{$chr}{$bi} * 1000000 / $rc;
         print TMP1 "$hash{$chr}{$bi}\n";
   print TMP2 "$rpm\n";
  }else{
      print TMP1 "0\n";
   print TMP2 "0\n";
  }
 }
   }
close TMP1;
close TMP2;
(my $bgp = $sbed) =~ s/bed$/p.bedgraph/;
system ("bedtools genomecov -split -strand + -bg -i ".$sbed." -g ".$prefix."/reference/".$genome."_chr_all.fasta.fai > ".$bgp);
open BGP, "<$bgp" or die $!;
(my $bgpo = $bgp) =~ s/bedgraph/$met\.bedgraph/;
open BGPO, ">$bgpo" or die $!;
while (my $rr = <BGP>){
 chomp $rr;
 my @row = split /\t/, $rr;
 $row[3] = $row[3] * 1000000 / $rc;
 print BGPO "$row[0]\t$row[1]\t$row[2]\t$row[3]\n";
}
close BGP;
close BGPO;
(my $bgn = $sbed) =~ s/bed$/n.bedgraph/;
system ("bedtools genomecov -split -strand - -bg -i ".$sbed." -g ".$prefix."/reference/".$genome."_chr_all.fasta.fai > ".$bgn);
open BGN, "<$bgn" or die $!;
(my $bgno = $bgn) =~ s/bedgraph/$met\.bedgraph/;
open BGNO, ">$bgno" or die $!;
while (my $rr = <BGN>){
 chomp $rr;
 my @row = split /\t/, $rr;
 $row[3] = $row[3] * 1000000 / $rc;
 print BGNO "$row[0]\t$row[1]\t$row[2]\t-$row[3]\n";
}
close BGN;
close BGNO;
(my $bg = $sbed) =~ s/bed$/bedgraph/;
system ("bedtools genomecov -split -bg -i ".$sbed." -g ".$prefix."/reference/".$genome."_chr_all.fasta.fai > ".$bg);
open BG, "<$bg" or die $!;
(my $bgo = $bg) =~ s/bedgraph/$met\.bedgraph/;
open BGO, ">$bgo" or die $!;
while (my $rr = <BG>){
 chomp $rr;
 my @row = split /\t/, $rr;
 $row[3] = $row[3] * 1000000 / $rc;
 print BGO "$row[0]\t$row[1]\t$row[2]\t$row[3]\n";
}
close BG;
close BGO;
unlink($bgp, $bgn, $bg);
system ("bedtools intersect -a gene.gff -b ".$sbed." -wa -c > ".$sbed.".gene.tmp");
system ("bedtools intersect -a promoter.gff -b ".$sbed." -wa -c > ".$sbed.".promoter.tmp");
system ("bedtools intersect -a te.gff -b ".$sbed." -wa -c > ".$sbed.".te.tmp");
open FILE, "$sbed.gene.tmp" or die $!;
my %out1 = ();
while(my $cc = <FILE>){
    chomp $cc;
    my @row = split /\t/, $cc;
    $out1{$row[8]} = $row[9];
}
close FILE;
open OUT1, ">$sbed\.gene" or die $!;
open OUT2, ">1.gene" or die $!;
```

```
    open OUT3, ">$sbed\.gene.norm" or die $!;
    foreach my $ge (sort keys %out1){
      print OUT1 "$out1{$ge}\n";
      print OUT2 "$ge\n";
      my $norm = $out1{$ge} * 1000000 / $rc;
      print OUT3 "$norm\n";
    }
    close OUT1;
    close OUT2;
    close OUT3;
    open FILE, "$sbed.te.tmp" or die $!;
    my %out2 = ();
    while(my $dd = <FILE>){
      chomp $dd;
      my @row = split /\t/, $dd;
      $out2{$row[8]} = $row[9];
    }
    close FILE;
    open OUT1, ">$sbed\.TE" or die $!;
    open OUT2, ">1.TE" or die $!;
    open OUT3, ">$sbed\.TE.norm" or die $!;
    foreach my $tt (sort keys %out2){
      print OUT1 "$out2{$tt}\n";
      print OUT2 "$tt\n";
      my $norm = $out2{$tt} * 1000000 / $rc;
      print OUT3 "$norm\n";
    }
    close OUT1;
    close OUT2;
    close OUT3;
    open FILE, "$sbed.promoter.tmp" or die $!;
    my %out3 = ();
    while(my $ee = <FILE>){
      chomp $ee;
      my @row = split /\t/, $ee;
      $out3{$row[8]} = $row[9];
    }
    close FILE;
    open OUT1, ">$sbed\.pro" or die $!;
    open OUT2, ">1.pro" or die $!;
    open OUT3, ">$sbed\.pro.norm" or die $!;
    foreach my $pp (sort keys %out3){
      print OUT1 "$out3{$pp}\n";
      print OUT2 "$pp\n";
      my $norm = $out3{$pp} * 1000000 / $rc;
      print OUT3 "$norm\n";
    }
    close OUT1;
    close OUT2;
    close OUT3;
    undef %out1;
    undef %out2;
    undef %out3;
    undef %hash;
    undef %hash2;
   }
   system ("paste 1.bin ".$tag."*.bin > ".$tag.".count");
     system ("paste 1.bin ".$tag."*.nbin > ".$tag.".".$met.".norm.count");
     system ("paste 1.new ".$tag."*.new > ".$tag.".miRNA.count");
     system ("paste 1.new ".$tag."*.nnew > ".$tag.".miRNA.".$met.".norm.count");
     system ("paste 1.gene ".$tag."*.gene > ".$tag.".gene.count");
   system ("paste 1.gene ".$tag."*.gene.norm > ".$tag.".gene.".$met.".norm.count");
   system ("paste 1.TE ".$tag."*.TE > ".$tag.".TE.count");
     system ("paste 1.TE ".$tag."*.TE.norm > ".$tag.".TE.".$met.".norm.count");
     system ("paste 1.pro ".$tag."*.pro > ".$tag.".promoter.count");
     system ("paste 1.pro ".$tag."*.pro.norm > ".$tag.".promoter.".$met.".norm.count");
   system ("rm ".$tag."*.new ".$tag."*.nnew ".$tag."*.tmp ".$tag."*.gene ".$tag."*.TE ".$tag."*.gene.norm ".$tag."*.TE.norm ".
$tag."*.bin ".$tag."*.nbin ".$tag."*.pro ".$tag."*.pro.norm");
   my (%mirna, %mi, %fas, $miseq, $nam);
   open MI, "$prefix/reference/".$genome."_chr_all.fasta" or die $!;
   while (my $mi = <MI>){
    chomp $mi;
    if($mi =~ />(.+)/){
     $nam = $1;
     $fas{$nam} = "";
```

```
  }else{
   $fas{$nam} .= $mi;
  }
 }
close MI;
open MI, "$prefix/reference/".$genome."_miRNA_miRNA_star.gff" or die $!;
while (my $mi = <MI>){
 chomp $mi;
 my @row = split /\t/, $mi;
 my $miseq = substr($fas{$row[0]}, $row[3]-1, $row[4]-$row[3]+1);
 if($row[6] eq "-"){
  $miseq =~ s/A/x/g;
  $miseq =~ s/T/A/g;
  $miseq =~ s/x/T/g;
  $miseq =~ s/C/x/g;
  $miseq =~ s/G/C/g;
  $miseq =~ s/x/G/g;
  $miseq = reverse $miseq;
 }
 $mirna{$miseq}{"name"} .= $row[8].";";
}
close MI;
foreach $miseq (keys %mirna){
 chop $mirna{$miseq}{"name"};
}
open MI, "$tag.miRNA.count" or die $!;
while (my $mi = <MI>){
 chomp $mi;
 my @row = split /\t/, $mi;
 foreach $miseq (keys %mirna){
  my @mi = split /;/, $mirna{$miseq}{"name"};
  for(my $i=0;$i<=$#mi;$i++){
   if($mi[$i] eq $row[0]){
    for(my $j=1;$j<=$#row;$j++){
     $mirna{$miseq}{"count"}{$j} += $row[$j];
    }
    last;
   }
  }
 }
}
close MI;
open MI, ">$tag.miRNA.count" or die $!;
foreach $miseq (sort keys %mirna){
 print MI "$mirna{$miseq}{name}";
 foreach my $count (sort {$a <=> $b} keys %{$mirna{$miseq}{"count"}}){
  print MI "\t$mirna{$miseq}{count}{$count}";
 }
 print MI "\n";
}
close MI;
open MI, "$tag.miRNA.$met.norm.count" or die $!;
while (my $mi = <MI>){
 chomp $mi;
 my @row = split /\t/, $mi;
 foreach $miseq (keys %mirna){
  my @mi = split /;/, $mirna{$miseq}{"name"};
  for(my $i=0;$i<=$#mi;$i++){
   if($mi[$i] eq $row[0]){
    for(my $j=1;$j<=$#row;$j++){
     $mirna{$miseq}{"count"}{$j} += $row[$j];
    }
    last;
   }
  }
 }
}
close MI;
open MI, ">$tag.miRNA.$met.norm.count" or die $!;
foreach $miseq (sort keys %mirna){
 print MI "$mirna{$miseq}{name}";
 foreach my $count (sort {$a <=> $b} keys %{$mirna{$miseq}{count}}){
  print MI "\t$mirna{$miseq}{count}{$count}";
 }
 print MI "\n";
```

```
    }
    close MI;
  }
  close NF;
  print STDERR "Counting Completed!\n";
  print STDOUT "Counting Completed!\n";
  #Clean up
  system ("rm -r ".$tag."*.fastq ".$tag.".rRNA.out ShortStack_".$tag." ".$tag."*.bed");
  print STDERR "Clean up complete!\n";
  print STDOUT "Clean up complete!\n";
}
if(!$nophasi){
  print STDERR "phasiRNA Analysis...\n";
  print STDOUT "phasiRNA Analysis...\n";
  my %hash3 = ();
  my $count = 0;
  my %data1 = ();
  my %data2 = ();
  my $command = "samtools merge ".$sample.".bam ";
  for(my $j=1;$j<=$num;$j++){
   $command .= $sample."_".$j.".rRNA-free.bam ";
      open my $rrna, "<$sample\_$j.nf" or die $!;
   while (my $r = <$rrna>){
    chomp $r;
    my ($rr, $base) = split /\t/, $r;
    next if($rr eq "rRNA" && $mnorm eq "t");
    next if($rr eq "total" && $mnorm eq "r");
    $hash3{$rr} += $base;
      }
      close $rrna;
  }
  system $command;
  system ("samtools view -h ".$sample.".bam > ".$sample.".sam");
  my (%gene_len, %phase_res);
  print STDERR "Reading SAM ...\n";
  print STDOUT "Reading SAM ...\n";
  open my $sam, "$sample.sam" or die $!;
  open PHASAM, ">$sample.phasi.sam" or die $!;
  while (my $ee = <$sam>) {
   chomp $ee;
   if ($ee =~ /^\s*$/){
    print PHASAM "$ee\n";
    next;
   }
   if ($ee =~ /^\@/){
    print PHASAM "$ee\n";
    next;
   }
   my ($readID, $flag, $chr, $pos, $MAPQ, $len, $line) = split /\t/, $ee;
   next if !($len eq '21M' or $len eq '22M');  #21-22nt siRNA
   print PHASAM "$ee\n";
   if ($flag == 0){
    $data1{$chr}{$pos}{0} ++;
    $count ++;
   }  #forward strand
   elsif ($flag == 16) {
    $data2{$chr}{$pos}{16} ++;
    $count ++;
   } #reverse strand
   if($count > 0 && $count % 100000 == 0){
    print STDERR "Read counts: $count\r";
   }
  }
  close PHASAM;
  system ("samtools view -Sb "."$sample".".phasi.sam > "."$sample".".phasi.bam");
  unlink $sample.".phasi.sam";
  print STDERR "Read counts: $count\n";
  print STDOUT "Read counts: $count\n";
  close $sam;
  print STDERR "Combining strands ...\n";
  print STDOUT "Combining strands ...\n";
  for my $chr (keys %data2) {
   for my $pos (keys %{$data2{$chr}}) {
    # 2-nt overhang
    if (exists $data1{$chr}{$pos + 2}{0}) {
```

```
    $data1{$chr}{$pos+2}{0} += $data2{$chr}{$pos}{16};
   } else {
    $data1{$chr}{$pos+2}{0} = $data2{$chr}{$pos}{16};
   }
  }
 }
 foreach my $norm (keys %hash3){
  my %data3 = ();
  my %phase_res = ();
     for my $chr (keys %data1) {
   for my $pos (keys %{$data1{$chr}}) {
       $data3{$chr}{$pos}{0} = $data1{$chr}{$pos}{0} * 1000000 / $hash3{$norm};
   }
     }
     print STDERR "Calculating phasing score ...\n";
  print STDOUT "Calculating phasing score ...\n";
     for my $chr (sort keys %data3) {
   print STDERR "$chr ...\r";
   for my $pos (sort {$a <=> $b} keys %{$data3{$chr}}) {
    my ($n, $phased, $unphased, $phasing_score) = (0, 0, 0, 0);
    for my $cycle (0..9) { # 10 cycles B.Meyers lab, RNA, 2009
     my $new_pos = $pos + 21 * $cycle; # phased position
     if (exists $data3{$chr}{$new_pos}{0}) {
      $n ++;  # num of pos plus 1
      $phased += $data3{$chr}{$new_pos}{0}; # phased cumulation
     }
     for my $unphased_nt_per_cycle (1..20) {
      $new_pos ++; # unphased positions
      if (exists $data3{$chr}{$new_pos}{0}) {
       $n ++; # num of pos plus 1
       $unphased += $data3{$chr}{$new_pos}{0};# unphased cumulation
      }
     }
    }
    if ($n < 3 || $phased < 10) {
     $phasing_score = 0;
    } else {
     $phasing_score = ($n - 2) * log (1 + 10 * $phased / ($unphased + 1)); # B.Meyers lab 2009
    }
    $phase_res{$chr}{$pos} = $phasing_score;
   }
     }
     my %hash2 = ();
     print STDERR "Outputing result ...\n";
     open my $phase_res_out, ">$sample.$norm.phasiRNA.txt";
     open my $bg, ">$sample.$norm.phasiRNA.bedgraph";
     open my $plotbg, ">$sample.$norm.plot.phasiRNA.bedgraph";
     print $phase_res_out "#BIN,Phase_score\n";
  for my $chr (sort keys %phase_res) {
   for my $pos (sort {$a <=> $b} keys %{$phase_res{$chr}}) {
    print $plotbg "$chr\t$pos\t$pos\t$phase_res{$chr}{$pos}\n";
    if($phase_res{$chr}{$pos} >= 50 && $data3{$chr}{$pos}{0} >= 1){
     print $bg "$pos\t$pos\t$phase_res{$chr}{$pos}\n";
     my $bin = int($pos/100);
     if(!exists $hash2{$chr}{$bin}){
      $hash2{$chr}{$bin} = $phase_res{$chr}{$pos};
     }elsif($phase_res{$chr}{$pos} > $hash2{$chr}{$bin}){
      $hash2{$chr}{$bin} = $phase_res{$chr}{$pos};
     }
    }
   }
  }
  for my $chr (sort keys %hash2) {
   for my $bin (sort {$a <=> $b} keys %{$hash2{$chr}}) {
    print $phase_res_out "$chr\_$bin,$hash2{$chr}{$bin}\n";
   }
  }
  close $phase_res_out;
  close $bg;
  close $plotbg;
  open TMP1, "$prefix/reference/".$genome.".annotation" or die $!;
  open TMP2, "$sample.$norm.phasiRNA.txt" or die $!;
  open TMP3, ">tmp3" or die $!;
  my %hash = ();
  while (my $aa = <TMP1>){
```

```
    chomp $aa;
    my @row = split /\t/, $aa;
    my $id = shift @row;
    $hash{$id} = join "\t", @row;
   }
   while(my $bb = <TMP2>){
    chomp $bb;
    my @row = split /,/, $bb;
    $row[0] =~ s/"//g;
    if(exists $hash{$row[0]}){
     print TMP3 "$bb,$hash{$row[0]}\n";
    }else{
     print TMP3 "$bb,NA\n";
    }
   }
   close TMP1;
   close TMP2;
   close TMP3;
   system ("mv tmp3 ".$sample.".".$norm.".phasiRNA.txt");
   undef %hash;
   undef %hash2;
   undef %data3;
   undef %phase_res;
  }
  undef %data1;
  undef %data2;
  undef %hash3;
  unlink $sample.".sam";
 }
 }
}
unlink ("gene.gff", "te.gff", "promoter.gff", "1.bin", "1.new", "1.gene", "1.TE", "1.pro");

####Statistic analysis
if(!$only){
 unshift @out, $foldchange;
 unshift @out, $pvalue;
 unshift @out, $mnorm;
 my $out = join " ", @out;
 print STDERR "DSR analysis...\n";
 print STDOUT "DSR analysis...\n";
 print STDERR "\tFold Change $foldchange\tP Value $pvalue\n";
 print STDOUT "\tFold Change $foldchange\tP Value $pvalue\n";
 system ("Rscript --vanilla ".$prefix."/scripts/DSR.R ".$out);
 opendir my $dir, "." or die $!;
 my @dir = grep {/.+hyper.csv$|.+hypo.csv$/} readdir $dir;
 closedir $dir;
 foreach my $hcsv (@dir){
  open CSV, "$hcsv" or die $!;
  my %hash = ();
  while (my $ff = <CSV>){
   chomp $ff;
   my @row = split /,/, $ff;
   if($row[0]=~/(Chr\w)_(\d+)/){
    my $chr = $1;
    my $bin = $2 * 100;
    $hash{$chr}{$bin}{"end"} = $bin + 99;
    $hash{$chr}{$bin}{"value"} = $row[2];
   }
  }
  (my $bg = $hcsv) =~ s/csv$/bedgraph/;
  open TMP, ">$bg" or die $!;
  foreach my $chr (sort keys %hash){
   foreach my $loc (sort {$a <=> $b} keys %{$hash{$chr}}){
    print TMP "$chr\t$loc\t$hash{$chr}{$loc}{end}\t$hash{$chr}{$loc}{value}\n";
   }
  }
  close TMP;
  close CSV;
 }
 opendir my $dir, "." or die $!;
 my @dir = grep {/csv$/} readdir $dir;
 closedir $dir;
 open ANN, "$prefix/reference/".$genome.".annotation" or die $!;
 my %hash = ();
```

```
 while (my $aa = <ANN>){
  chomp $aa;
  my @row = split /\t/, $aa;
  my $id = shift @row;
  $hash{$id} = join "\t", @row;
 }
 close ANN;
 foreach my $csv (@dir){
  open CSV, "$csv" or die $!;
  open TMP, ">tmp4" or die $!;
  while(my $bb = <CSV>){
   chomp $bb;
   my @row = split /,/, $bb;
   $row[0] =~ s/"//g;
   if(exists $hash{$row[0]}){
    print TMP "$bb,$hash{$row[0]}\n";
   }else{
    print TMP "$bb,NA\n";
   }
  }
  close CSV;
  close TMP;
  system ("mv tmp4 ".$csv);
 }

 print STDERR "\nDE miRNA analysis...\n";
 print STDOUT "\nDE miRNA analysis...\n";
 print STDERR "\tFold Change $foldchange\tP Value $pvalue\n";
 print STDOUT "\tFold Change $foldchange\tP Value $pvalue\n";
 system ("Rscript --vanilla ".$prefix."/scripts/DEM.R ".$out);

 print STDERR "\nDS gene analysis...\n";
 print STDOUT "\nDS gene analysis...\n";
 print STDERR "\tFold Change $foldchange\tP Value $pvalue\n";
 print STDOUT "\tFold Change $foldchange\tP Value $pvalue\n";
 system ("Rscript --vanilla ".$prefix."/scripts/DSG.R ".$out);

 print STDERR "\nDS TE analysis...\n";
 print STDOUT "\nDS TE analysis...\n";
 print STDERR "\tFold Change $foldchange\tP Value $pvalue\n";
 print STDOUT "\tFold Change $foldchange\tP Value $pvalue\n";
 system ("Rscript --vanilla ".$prefix."/scripts/DST.R ".$out);

 print STDERR "\nDS Promoter analysis...\n";
 print STDOUT "\nDS Promoter analysis...\n";
 print STDERR "\tFold Change $foldchange\tP Value $pvalue\n";
 print STDOUT "\tFold Change $foldchange\tP Value $pvalue\n";
 system ("Rscript --vanilla ".$prefix."/scripts/DSP.R ".$out);
}

####Cleaning

 mkdir $output if(!$nophasi || !$only);
 if(!$nophasi && !$nomapping){
  system ("mv *phasiRNA.bedgraph *phasiRNA.txt ".$output);
 }
 if(!$only){
  system ("mv *.pdf *".$out[3]."*.csv ".$output);
 }
 shift @out;
 for my $oo (@out){
  next if ($oo =~ /^\d+\.?\d*$/);
  system ("mv ".$oo."*.bedgraph ".$output);
 }
 if(!$nophasi || !$only){
  system ("mv log.txt ".$output);
 }

####END

}elsif($mode eq "mrna"){

####Mapping start

 my @out;
```

```
while(@ARGV){
 my $sample = shift @ARGV;
 push @out, $sample;
 my $num = shift @ARGV;
 die "Please provide enough biological replicates\ntype \"-h\" for more information" if($num < 1);
 push @out, $num;
 if(!$nomapping){
  my $id = "";
  my ($idt, $ind, $chr, %index);
  open GFF, "$prefix/reference/".$genome."_genes.gff" or die $!;
  while(my $aa = <GFF>){
   chomp $aa;
   my @row = split /\t/, $aa;
   next if($row[2] =~ /UTR/ || $row[2] =~ /c_transcript/ || $row[2] =~ /region/);
   next if($row[2] eq "protein" || $row[2] eq "CDS");
   next if($row[2] =~ /[^i]RNA/);
   if($row[2] =~ /gene/){
    if($row[8] =~ /^ID=(AT[\dCM]\w+\d+);/o){
     $id = $1;
     $ind = int($row[3]/100000);
     $chr = $row[0];
     $index{$chr}{$ind}{$id}{start} = $row[3];
     $index{$chr}{$ind}{$id}{end} = $row[4];
     $index{$chr}{$ind+1}{$id}{start} = $row[3];
     $index{$chr}{$ind+1}{$id}{end} = $row[4];
     $index{$chr}{$ind-1}{$id}{start} = $row[3];
     $index{$chr}{$ind-1}{$id}{end} = $row[4];
    }
   }elsif($row[8] =~ /Parent=$id/){
     $index{$chr}{$ind}{$id}{exon} .= $row[3]."\t".$row[4].";";
     $index{$chr}{$ind+1}{$id}{exon} .= $row[3]."\t".$row[4].";";
     $index{$chr}{$ind-1}{$id}{exon} .= $row[3]."\t".$row[4].";";
   }
  }
  close GFF;
  my (%out, %hash);
  foreach my $chr (sort keys %index){
   foreach my $ind (sort keys %{$index{$chr}}){
    foreach my $gene (sort keys %{$index{$chr}{$ind}}){
     my @exon = split /;/, $index{$chr}{$ind}{$gene}{exon};
     foreach my $zz (@exon){
      $hash{$zz} = "";
     }
     my @exon = sort keys %hash;
     $index{$chr}{$ind}{$gene}{exon} = join ";", @exon;
     undef %hash;
     $out{$gene}{exon_count} = 0;
     $out{$gene}{intron_count} = 0;
    }
   }
  }
  for(my $i=1;$i<=$num;$i++){
   my $file = shift @ARGV;
   my $oo = $sample."_".$i;
   print STDERR "Mapping $oo...\n";
   print STDOUT "Mapping $oo...\n";
   if($file !~ /,/){
    if($adapter ne ""){
     &trimming($file, $oo);
     unlink $oo.".rRNA.out";
     system ("hisat2 --max-intronlen 60000 -p ".$core." --dta -x ".$prefix."/reference/".$genome."_chr_all -U ".$oo."-rRNA-free.fastq -S ".
$oo.".sam 2>>log.txt");
     unlink ($oo."-rRNA-free.fastq", $oo."_trimmed.fastq");
    }
    else{
     if(! -e $file && $file =~ /^SRR\d+/){
      print STDERR "Downloading...\n";
      print STDOUT "Downloading...\n";
      system ("prefetch.2.8.2 ".$file."; mv ~/ncbi/public/sra/".$file.".sra .");
      system ("fastq-dump.2.8.2 ".$file.".sra; rm ".$file.".sra");
      $file .= ".fastq";
     }
     system ("hisat2 --max-intronlen 60000 -p ".$core." --dta -x ".$prefix."/reference/".$genome."_chr_all -U ".$file." -S ".$oo.".sam
2>>log.txt");
    }
```

```
   }else{
    my @file = split /,/, $file;
    die "Please provide correct files\ntype \"-h\" for more information" if($#file > 1);
    if($adapter ne ""){
     &trimming($file[0], $oo."_R1");
     &trimming($file[1], $oo."_R2");
     unlink ($oo."_R1.rRNA.out", $oo."_R2.rRNA.out");
     system ("hisat2 --max-intronlen 60000 -p ".$core." --dta -x ".$prefix."/reference/".$genome."_chr_all -1 ".$oo."_R1-rRNA-free.fastq -2 ".$oo."_R2-rRNA-free.fastq -S ".$oo.".sam 2>>log.txt");
     unlink ($oo."_R1-rRNA-free.fastq", $oo."_R2-rRNA-free.fastq", $oo."_R1_trimmed.fastq", $oo."_R2_trimmed.fastq");
    }else{
     if(! -e $file[0] && $file[0] =~ /^SRR\d+/){
      print STDERR "Downloading...\n";
      print STDOUT "Downloading...\n";
      system ("prefetch.2.8.2 ".$file[0]."; mv ~/ncbi/public/sra/".$file[0].".sra .");
      system ("fastq-dump.2.8.2 ".$file[0].".sra; rm ".$file[0].".sra");
      $file[0] .= ".fastq";
     }
     if(! -e $file[1] && $file[1] =~ /^SRR\d+/){
      print STDERR "Downloading...\n";
      print STDOUT "Downloading...\n";
      system ("prefetch.2.8.2 ".$file."; mv ~/ncbi/public/sra/".$file[1].".sra .");
      system ("fastq-dump.2.8.2 ".$file[1].".sra; rm ".$file[1].".sra");
      $file[1] .= ".fastq";
     }
     system ("hisat2 --max-intronlen 60000 -p ".$core." --dta -x ".$prefix."/reference/".$genome."_chr_all -1 ".$file[0]." -2 ".$file[1]." -S ".$oo.".sam 2>>log.txt");
    }
   }
   system ("samtools view -Sb ".$oo.".sam > ".$oo.".bam");
   system ("samtools sort -@ ".$core." -o ".$oo.".bam ".$oo.".bam");
   print STDERR "Start counting...\n";
   print STDOUT "Start counting...\n";
   open SAM, "<$oo.sam" or die $!;
   my $m;
   while (my $bb = <SAM>) {
    next if($bb =~ /^@/);
    $m ++;
    if($m % 500000 == 0){
     print STDERR "Read Count:$m\n";
    }
    chomp $bb;
    my @row = split /\t/, $bb;
    next if($row[5] =~ /[IDSPHX=]/);
    my $chr = $row[2];
    my $start = $row[3];
    my $ind = int($start/100000);
    my $skip = 0;
    my $length = 0;
    my $n = 0;
    my $k = 0;
    my (@xx, $yy);
    undef @xx;
    my @map = split /N/, $row[5];
    foreach my $seg (@map){
     if ($seg =~ /(\d+)M(\d+)/) {
      $yy = $start + $1 -1;
      $xx[$n] = $start.";".$yy;
      $length += $1;
      $skip += $2;
      $n ++;
      $start = $start + $1 + $2;
     }elsif ($seg =~ /(\d+)M/){
      $length += $1;
      $yy = $start + $1 -1;
      $xx[$n] = $start.";".$yy;
     }
    }
    foreach my $id (sort keys %{$index{$chr}{$ind}}){
     if ($row[3] + 5 >= $index{$chr}{$ind}{$id}{start} && $yy <= $index{$chr}{$ind}{$id}{end} + 5) {
      foreach my $part (@xx){
       my @site = split /;/, $part;
       foreach my $exon (split /;/, $index{$chr}{$ind}{$id}{exon}){
        my @tmp = split /\t/, $exon;
        if ($site[0] + 1 >= $tmp[0] && $site[1] <= $tmp[1] + 1) {
```

```
          $k ++;
          last;
         }
        }
       }
       if ($k == $n + 1) {
        $out{$id}{exon_count} ++;
       }else{
        $out{$id}{intron_count} ++;
       }

      }
     }
    }
    print STDERR "Read Count:$m\n";
    print STDOUT "Read Count:$m\n";
    close SAM;
    open OUT, ">$oo.txt" or die $!;
    print OUT "Transcript\tExon\tIntron\tTotal\tLength\n";
    foreach my $name (sort keys %out){
     my $total = $out{$name}{exon_count} + $out{$name}{intron_count};
     print OUT "$name\t$out{$name}{exon_count}\t$out{$name}{intron_count}\t$total\t$geneinfo{$name}{long}\n";
    }
    unlink $oo.".sam";
   }
  }
 }

###Statistic Analysis

 if(!$only){
  print STDERR "Fold Change\t$foldchange\tP Value\t$pvalue\n";
  print STDOUT "Fold Change\t$foldchange\tP Value\t$pvalue\n";
  my $put = join " ", @out;
  system ("Rscript --vanilla ".$prefix."/scripts/DEG.R ".$pvalue." ".$foldchange." ".$put);
  system ("mkdir ".$output."; mv *.pdf *.csv *.txt ".$output);
  system ("mv nohup.out ".$output) if(-e "nohup.out");
 }

###END

}elsif($mode eq "tt"){

### Mapping Start

 open GFF, "$prefix/reference/$genome\_miRNA_miRNA_star.gff" or die $!;
 my %mir;
 while (my $bb = <GFF>){
  chomp $bb;
  my @row = split /\t/, $bb;
  if($row[6] eq "+"){
   $mir{$row[0]}{$row[3]}{name} = $row[8];
   $mir{$row[0]}{$row[3]}{strand} = 0;
   $mir{$row[0]}{$row[3]}{length} = $row[4] - $row[3] + 1;
  }else{
   $mir{$row[0]}{$row[4]}{name} = $row[8];
   $mir{$row[0]}{$row[4]}{strand} = 16;
   $mir{$row[0]}{$row[4]}{length} = $row[4] - $row[3] + 1;
  }
 }
 close GFF;

 my @out;
 while(@ARGV){
  my $sample = shift @ARGV;
  push @out, $sample;
  my $num = shift @ARGV;
  die "Please provide enough biological replicates\ntype \"-h\" for more information\n" if($num < 1);
  push @out, $num;

###Mapping start

  my $tag;
  for(my $i=1;$i<=$num;$i++){
   my $file = shift @ARGV;
```

```
  $tag = $sample."_".$i;
  print STDERR "Mapping $tag...\n";
  print STDOUT "Mapping $tag...\n";
  &trimming($file, $tag);
  unlink $tag.".rRNA.out";
  system ("bowtie -v 0 -p ".$core." -t --un ".$tag.".unmapped_0.fastq --al ".$tag.".mapped.fastq ".$prefix."/reference/".
$genome."_chr_all ".$tag."-rRNA-free.fastq ".$tag.".out 2>>log.txt");
  my ($seq, $plus, $qua, $id);
  for(my $p = 1; $p <= 8; $p ++){
    my $j = $p - 1;
    open FQ, "<$tag.unmapped_$j.fastq";
    open OUT, ">tmp.fastq";
    my $n = 1;
    while(my $aa = <FQ> ){
      chomp $aa;
      if($n == 4){
        $qua = substr($aa, 0, -1);
     if(length($seq) >= 14){
          print OUT "$id\n$seq\n$plus\n$qua\n";
     }
        $n = 1;
        next;
      }elsif($n == 3){
        $plus = $aa;
        $n = 4;
      }elsif($n == 2){
        $seq = substr($aa, 0, -1);
        $n = 3;
      }elsif($n == 1){
     my @row = split /\s/, $aa;
     $row[0] =~ s/_\d$//;
        $id = $row[0]."_".$p;
        $n = 2;
      }
    }
    close OUT;
    close FQ;
    unlink ($tag.".unmapped_$j.fastq");
    system ("bowtie -v 0 -p ".$core." -t --un ".$tag.".unmapped_".$p.".fastq --al ".$tag.".mapped_".$p.".fastq ".$prefix."/reference/".
$genome."_chr_all tmp.fastq ".$tag.".out 2>>log.txt");
  }
  system ("cat $tag.mapped*.fastq > ".$tag."_edited.fastq");
  system ("rm $tag*mapped* tmp.fastq");
  system ("ShortStack --outdir ".$tag."tmp --align_only --bowtie_m 1000 --ranmax 50 --mmap ".$mmap." --mismatches 0 --bowtie_cores
".$core." --nohp --readfile ".$tag."_edited.fastq --genomefile ".$prefix."/reference/".$genome."_chr_all.fasta 2>>log.txt");
  system ("samtools view -h ".$tag."tmp/$tag\_edited.bam > $tag");
  system ("awk '{if(\$10!=\"*\" && \$3!=\"*\") print > (FILENAME\".edited.sam\")}' ".$tag);
  system ("rm -r ".$tag."tmp $tag $tag-rRNA-free.fastq $tag\_edited.fastq $tag\_trimmed.fastq");
  print STDERR "Alignment Completed!\n";
  print STDOUT "Alignment Completed!\n";
  open SAM, "$tag.edited.sam" or die $!;
  my %out;
  while (my $cc = <SAM>){
   next if ($cc =~ /^@/);
   chomp $cc;
   my @data = split /\t/, $cc;
   $data[5] =~ s/M//;
   $data[3] = $data[3] + $data[5] - 1 if($data[1] == 16);
   my $tail = 0; my $trunc = 0;
   if(exists $mir{$data[2]}{$data[3]}{name}){
    if($data[1] == $mir{$data[2]}{$data[3]}{strand}){
     $data[5] =~ s/M//;
     my $mirna = $mir{$data[2]}{$data[3]}{name};
     if($data[5] >= $mir{$data[2]}{$data[3]}{length}){
      $tail = $data[5] - $mir{$data[2]}{$data[3]}{length};
     }else{
      $trunc = $mir{$data[2]}{$data[3]}{length} - $data[5];
      my ($seq, $tail) = split /\_/, $data[0];
     }
     $tail = 0 if(!$tail);
     $out{$mirna}{$tail}{$trunc} ++;
    }
   }
  }
  close SAM;
```

```
   unlink ("$tag.edited.sam");

   open OUT, ">$tag.out" or die $!;
   foreach my $mm (sort keys %out){
    for my $i (0 .. 10){
     for my $j (0 .. 10){
      if(exists $out{$mm}{$i}{$j}){
       print OUT "$mm\t$i\t$j\t$out{$mm}{$i}{$j}\n";
      }else{
       print OUT "$mm\t$i\t$j\t0\n";
      }
     }
    }
   }
   close OUT;
  }
 }
###Plotting
 my $put = join " ", @out;
 system ("Rscript --vanilla ".$prefix."/scripts/bubble_plot.R ".$put);
 system ("mkdir ".$output."; mv *.pdf ".$output);

###END

}elsif($mode eq "deg"){

### Mapping Start

 my @out;
 while(@ARGV){
  my $sample = shift @ARGV;
  push @out, $sample;
  my $num = shift @ARGV;
  die "Please provide enough biological replicates\ntype \"-h\" for more information" if($num < 1);
  push @out, $num;
  my $tag;
  for(my $i=1;$i<=$num;$i++){
   my $file = shift @ARGV;
   $tag = $sample."_".$i;
   print STDERR "Mapping $tag...\n";
   print STDOUT "Mapping $tag...\n";
   &trimming($file, $tag);
   unlink $tag.".rRNA.out";
   system ("ShortStack --outdir ShortStack_".$tag." --align_only --nostitch --bowtie_m 1000 --ranmax 50 --mmap ".$mmap." --mismatches
0 --bowtie_cores ".$core." --nohp --readfile ".$tag."-rRNA-free.fastq --genomefile ".$prefix."/reference/".$genome."_transcripts.fasta
2>>log.txt");
   print STDERR "Alignment Completed!\n";
   print STDOUT "Alignment Completed!\n";
   system ("samtools view -h ShortStack_".$tag."/".$tag."-rRNA-free.bam > ".$tag.".rRNA-free");
   system ("awk '{if(\$0~/^@/) print > (FILENAME\".unmapped.sam\"); if(\$10!=\"*\" && \$3!=\"*\") print > (FILENAME\".sam\"); if(\$10!
=\"*\" && \$3==\"*\") print > (FILENAME\".unmapped.sam\")}' ".$tag.".rRNA-free");
   system ("samtools view -Sb ".$tag.".rRNA-free.unmapped.sam > ".$tag.".rRNA-free.unmapped.bam");
   system ("samtools view -Sb ".$tag.".rRNA-free.sam > ".$tag.".rRNA-free.bam");
   unlink ($tag."-rRNA-free.fastq", $tag.".rRNA-free", $tag.".rRNA-free.unmapped.sam");
   system ("rm -r ShortStack_".$tag);

### Calling Peaks

   my $name = "";
   my ($tt, %hash, @counts, $mean, $std, @sites, $sum);
   my $tto = `wc -l $tag.rRNA-free.sam`;
   open SAM, "$tag.rRNA-free.sam" or die $!;
   open OUT, ">$tag.peaks" or die $!;
   while(my $bb = <SAM>){
    chomp $bb;
    next if($bb =~ /^@/);
    my @row = split /\t/, $bb;
    next if($row[2] eq "*");
    if($name eq $row[2]){
     $hash{$row[3]} ++;
     $tt ++;
    }else{
     @sites = sort {$a <=> $b} keys %hash;
     if($#sites >= 9){
      foreach my $si (@sites){
```

```
      push @counts, $hash{$si};
     }
     $mean = &average(@counts);
     $std = &stdev(@counts);
     $sum = &sum(@counts);
     for(my $i=0;$i<=$#sites;$i++){
      if($hash{$sites[$i]} - $mean >= 5 * $std && $hash{$sites[$i]} * 1000000 / $tto >= 5 && $hash{$sites[$i]} * 10 >= $sum){
       print OUT "$name\t$sites[$i]\t$#sites\t$tt\t$hash{$sites[$i]}\t$mean\t$std\n";
      }
     }
    }
    $name = $row[2];
    undef %hash;
    undef $tt;
    undef @counts;
    $hash{$row[3]} ++;
    $tt ++;
   }
  }
  @sites = sort {$a <=> $b} keys %hash;
  if($#sites >= 9){
   foreach my $si (@sites){
    push @counts, $hash{$si};
   }
   $mean = &average(@counts);
   $std = &stdev(@counts);
   $sum = &sum(@counts);
   for(my $i=0;$i<=$#sites;$i++){
    if($hash{$sites[$i]} - $mean >= 5 * $std && $hash{$sites[$i]} * 1000000 / $tto >= 5 && $hash{$sites[$i]} >= 0.05 * $sum){
     print OUT "$name\t$sites[$i]\t$#sites\t$tt\t$hash{$sites[$i]}\t$mean\t$std\n";
    }
   }
  }
  close SAM;
  close OUT;
 }
}

}elsif($mode eq "clip"){

 my @out;
 while(@ARGV){
  my $sample = shift @ARGV;
  push @out, $sample;
  my $num = shift @ARGV;
  die "Please provide enough biological replicates\ntype \"-h\" for more information" if($num < 1);
  push @out, $num;
  my $tag;
  for(my $i=1;$i<=$num;$i++){
   my $file = shift @ARGV;
   $tag = $sample."_".$i;
   print STDERR "Mapping $tag...\n";
   print STDOUT "Mapping $tag...\n";
   &trimming($file, $tag);
   system ("ShortStack --outdir ShortStack_".$tag." --align_only --nostitch --bowtie_m 1000 --ranmax 50 --mmap ".$mmap." --mismatches
".$mis." --bowtie_cores ".$core." --nohp --readfile ".$tag."_trimmed.fastq --genomefile ".$prefix."/reference/".$genome."_chr_all.fasta
2>>log.txt");
   print STDERR "Alignment Completed!\n";
   print STDOUT "Alignment Completed!\n";
   system ("samtools view -h ShortStack_".$tag."/".$tag."_trimmed.bam > ".$tag."_trimmed");
   system ("awk '{if(\$3!=\"*\" && \$10!=\"*\") print > (FILENAME\".sam\")}' ".$tag."_trimmed");
   system ("awk '{OFS=\"\t\"}{if(\$2==16){\$2=0}else if(\$2==0){\$2=16}; print \$0}' ".$tag."_trimmed.sam > tmp; mv tmp ".
$tag."_trimmed.sam");
   system ("samtools view -Sb ".$tag."_trimmed.sam > ".$tag."_trimmed.bam");
   unlink ($tag."_trimmed.fastq", $tag."_trimmed");
   system ("rm -r ShortStack_".$tag);
   print STDERR "Calling Peaks...";
   print STDOUT "Calling Peaks...";
   system ("clipper ".$pre." --minreads=5 --FDR=0.01 --threshold-method=binomial --processors=".$core." -s ".$genome." -b ".
$tag."_trimmed.bam -o ".$tag."_fitted_clusters.bed 2>>log.txt");
   unlink ($tag."_trimmed.sam");
   print STDERR "CLIPper Complete!";
   print STDOUT "CLIPper Complete!";
  }
 }
```

```
}

print STDERR "\nMission Completed!\n";
print STDOUT "\nMission Completed!\n";
$time = localtime;
print STDERR "End: $time\n";
print STDOUT "End: $time\n";
close STDOUT;
close STDERR;

sub sum{
    my $total = 0;
    foreach (@_) {
  $total += $_;
    }
    return $total;
}

sub average{
    my $total = &sum(@_);
    my $average = $total / @_;
    return $average;
}

sub stdev{
    my $average = &average(@_);
    my $sqtotal = 0;
    foreach(@_) {
        $sqtotal += ($average-$_) ** 2;
    }
    my $std = ($sqtotal / (@_-1)) ** 0.5;
    return $std;
}

sub trimming{
 my ($file, $tag) = @_;

 #Step 1 Remove adapter sequences
 if(-e $file){
  if($file =~ /bz2$/){
   print STDERR "Decompressing...\n";
   print STDOUT "Decompressing...\n";
   system ("bzip2 -dc ".$file." > ".$tag.".fastq");
  }elsif($file =~ /gz$/){
   print STDERR "Decompressing...\n";
   print STDOUT "Decompressing...\n";
   system ("gzip -dc ".$file." > ".$tag.".fastq");
  }elsif($file =~ /fastq$|fq$/){
   if ($file ne $tag.".fastq"){
    print STDERR "Renaming...\n";
    print STDOUT "Renaming...\n";
    system ("cp ".$file." ".$tag.".fastq");
   }else{
    print STDERR "Backing up...\n";
    print STDOUT "Backing up...\n";
    system ("cp ".$file." ".$tag.".fastq.bak");
   }
  }
 }elsif($file =~ /^SRR\d+/){
  print STDERR "Downloading...\n";
  print STDOUT "Downloading...\n";
  system ("prefetch.2.8.2 ".$file."; mv ~/ncbi/public/sra/".$file.".sra .");
  system ("fastq-dump.2.8.2 ".$file.".sra; mv ".$file.".fastq ".$tag.".fastq; rm ".$file.".sra");
 }else{
  die "Please provide the seq file or the seq file in correct format!";
 }
 print STDERR "Start trimming...\r";
 if($mode eq "srna"){
  system ("cutadapt -m 18 -M 42 --discard-untrimmed --trim-n -f fastq -a ".$adapter." -o ".$tag."_trimmed.fastq ".$tag.".fastq
2>>log.txt");
 }elsif($mode eq "mrna"){
  system ("cutadapt -m 20 --discard-untrimmed --trim-n -f fastq -a ".$adapter." -o ".$tag."_trimmed.fastq ".$tag.".fastq 2>>log.txt");
 }elsif($mode eq "tt"){
  system ("cutadapt -m 14 -M 42 --discard-untrimmed --trim-n -f fastq -a ".$adapter." -o ".$tag."_trimmed.fastq ".$tag.".fastq
2>>log.txt");
```

```
}elsif($mode eq "deg"){
 system ("cutadapt -m 19 -M 21 --discard-untrimmed --trim-n -f fastq -a ".$adapter." -o ".$tag."_trimmed.fastq ".$tag.".fastq
2>>log.txt");
 }elsif($mode eq "clip"){
 system ("cutadapt -u 3 -m 10 --discard-untrimmed --trim-n -f fastq -a ".$adapter." -o ".$tag."_trimmed.fastq ".$tag.".fastq 2>>log.txt");
 }
 unlink ($tag.".fastq");
 print STDERR "Trimming Completed!\n";
 print STDOUT "Trimming Completed!\n";
 #Step 2 Filter and remove reads with homology to rRNA, tRNA, etc.
 if($mode ne "clip"){
  system ("bowtie -v 2 -k1 -p ".$core." -t --un ".$tag."-rRNA-free.fastq --al ".$tag.".rRNA.fastq ".$prefix."/reference/".
$genome."_other_rna ".$tag."_trimmed.fastq ".$tag.".rRNA.out 2>>log.txt");
  unlink ($tag.".rRNA.fastq");
  print STDERR "other RNA Filtering Completed!\n";
  print STDOUT "other RNA Filtering Completed!\n";
 }
}
```

Data analysis

```
#!/usr/bin/perl
# Copyright (c)  2010-
# Program:   sRNA_dist_lib
# Author:   Gaolei <highlei@gmail.com or leigao@ucr.edu>
# Program Date:  2010.03.11
# Modifier:   Gaolei <highlei@gmail.com or leigao@ucr.edu>
# Last Modified: 2014.03.24
# Description: the distribution of miRNA in every lib
#**************************
# Version: 1.1 use the soap results and normalize the data.
# Version: 1.2 fix bugs; use $c
# Version: 1.3 use eq replace the =~ when compare mature miRNAs with small RNAs.
# Version: 1.4 use the input library_size
# Version: 1.5 $inclusion: perfect match or inclusion
# Version: 2.0 use the mature miRNAs
# Version: 2.1 can read fasta file without format >ath1_89_90x
# Version: 3.0 the distribution of miRNA and its truncation and tailing in every lib
#**************************
# e-mail:highlei@gmail.com

my $version="3.0";
print STDERR ("\n==================| $0 start |==========================================\n");

my $start = time();
my $Time_Start = sub_format_datetime(localtime(time())); #运行开始时间
print STDERR "Now = $Time_Start\n\n";


use Getopt::Std;
getopts("hi:d:s:e:m:b:g:a:n:c:S:N:L:O:I:A:l:0:");
my $flag0  = (defined $opt_0) ? $opt_0 : 1;
my $infile  = $opt_i;
my $libFile  = (defined $opt_d) ? $opt_d : "";
#my $startPos = (defined $opt_s) ? $opt_s : 2;
#my $endPos  = (defined $opt_e) ? $opt_e : 13;
#my $mismatch = (defined $opt_m) ? $opt_m : 1;
#my $bulge  = (defined $opt_b) ? $opt_b : 1; # = gap
#my $GUpair  = (defined $opt_g) ? $opt_g : 0.5;
#my $double  = (defined $opt_a) ? $opt_a : 2; #
my $start_len = (defined $opt_l) ? $opt_l : 8; # max truncation length
my $soap_result = (defined $opt_S) ? $opt_S : "";
my $normal_base = (defined $opt_N) ? $opt_N : 10000000;
my $lib_size = (defined $opt_s) ? $opt_s : ""; ## the libraries size
my $library  = (defined $opt_L) ? $opt_L : ""; ## the libraries size
my $opformat = (defined $opt_O) ? $opt_O : 1; # 0: +, -; 1: + plus -; 2: +; 3: -
my $inclusion = (defined $opt_I) ? $opt_I : 0; # 0: match; 1: include; 2: 4kind T&T; 3: all T&T
my $ask_input = (defined $opt_A) ? $opt_A : 1;
#my $trunction = (defined $opt_T) ? $opt_T : 0; # 0: no truncation; 1: do truncation

if ($opt_h || $infile eq ""){
 usage();
}


sub numerically{$a<=>$b};
```

```
use FileHandle;
use strict;

my ($i,$j,$k,$m,$n,$k1,$k2,$k3,$k4,$file,$line,$in,$match,$omatch,$a,$b,$end);
my (@buf,@tmp,@genome,@gnmName,@gnmLen);
my (%gnm,%seg,%num,%matureSeq);
my $key="";
my ($endLen,$minLen,$addLen,$maxLen,$foldFileNum,$bfile);
my ($foldFile,$foldFileOut);

#================================================================================================================
#====================                    main
#================================================================================================================
#my $flag0 = 1;
my $yesorno = "y";
while ($flag0) {
 print STDERR ("\n------------------------------------------------------------\n");
 print STDERR ("\n $0 version $version\n\n");
 print STDERR ("Settings for this run:");
 printf STDERR ("\n i  %55s : %-25s","input miRNA file",$infile);#%45s
 printf STDERR ("\n d  %55s : %-25s","input library file(s)",$libFile);
 printf STDERR ("\n S  %55s : %-25s","input soap result file(s)",$soap_result);
 printf STDERR ("\n N  %55s : %-25s","input the base for normalization",$normal_base);
 printf STDERR ("\n s  %55s : %-25s","input library size",$lib_size);
 printf STDERR ("\n L  %55s : %-25s","input library",$library);
 printf STDERR ("\n O  %55s : %-25s","output format",$opformat);
 printf STDERR ("\n I  %55s : %-25s","perfect match or inclusion or Truncation & Tailing",$inclusion);
 printf STDERR ("\n A  %55s : %-25s","ask input or not",$ask_input);
 printf STDERR ("\n l  %55s : %-25s","input the max truncation length",$start_len);
# if($zero==1) {printf STDERR ("\n z  %45s : %-25s","output coverage region?","1");}
# elsif ($zero == 0) {printf STDERR ("\n z  %45s : %-25s","concise output","0");}
# else {printf STDERR ("\n z  %45s : %-25s","output all genome","2");}
 printf STDERR ("\n x  %55s","exit the program!");
 print STDERR ("\n\n");
 print STDERR "y to accept these or type the letter for one to change!\n";
 $yesorno = <STDIN>; $yesorno =~s/[\s|\t|\r|\n]+$//g; $yesorno = lc($yesorno);
 if ($yesorno eq "y") {print STDERR ("\n------------------------------------------------------------\n\n\n"); $flag0 = 0;}
 elsif($yesorno eq "i") {print STDERR "please input miRNA file:\n"; $infile = <STDIN>; $infile =~s/[\s|\t|\r|\n]+$//g;}
 elsif($yesorno eq "d") {print STDERR "please input library file(s):\n"; $libFile = <STDIN>;$libFile =~s/[\s|\t|\r|\n]+$//g;}
 elsif($yesorno eq "S") {print STDERR "please input soap result file(s):\n"; $soap_result = <STDIN>;$soap_result =~s/[\s|\t|\r|\n]+$//g;}
 elsif($yesorno eq "N") {print STDERR "please input the base for normalization:\n";$normal_base = <STDIN>;$normal_base =~s/[\s|\t|\r|
\n]+$//g;}
 elsif($yesorno eq "s") {print STDERR "please input library size:\n";$lib_size = <STDIN>;$lib_size =~s/[\s|\t|\r|\n]+$//g;}
 elsif($yesorno eq "L") {print STDERR "please input library:\n";$library = <STDIN>;$library =~s/[\s|\t|\r|\n]+$//g;}
 elsif($yesorno eq "O") {print STDERR "please output format (0: +,-; 1:+ plus -; 2:+; 3:-):\n";$opformat = <STDIN>;$opformat =~s/[\s|\t|\r|
\n]+$//g;}
 elsif($yesorno eq "I") {print STDERR "please input 0: match; 1: include; 2: 4kind T&T; 3: all T&T:\n";$inclusion = <STDIN>;$inclusion =~s/
[\s|\t|\r|\n]+$//g;}
 elsif($yesorno eq "A") {print STDERR "please input ask iput (1) or not (0):\n";$ask_input = <STDIN>;$ask_input =~s/[\s|\t|\r|\n]+$//g;}
 elsif($yesorno eq "l") {print STDERR "please input the max trunction length:\n";$start_len = <STDIN>;$start_len =~s/[\s|\t|\r|\n]+$//g;}

 elsif($yesorno eq "x") {print STDERR ("============================================================\n");exit(0);}
}

if ($lib_size ne "") {
 @tmp = split(/\,/,$lib_size);
 print STDERR "\tlib_size: @tmp\t","\n";
 for ($k1 = 0; $k1 < @tmp ;$k1++) {
  $i = $k1;
  $num{$k1}->[0] = $k1;
  $num{$k1}->[1] = $tmp[$k1];
  print "Soap $k1,\tnum=",$num{$i}->[1]; print STDERR "\tSoap $i, $k1,\tnum=",$num{$i}->[1];
  $num{$i}->[1] = 1.0*$num{$i}->[1]/$normal_base;
  print "\t normalize=",$num{$i}->[1],"\n"; print STDERR "\t normalize=",$num{$i}->[1],"\n";
 }
}

################################################ read file ############################################################


$k1 = 0; $k3 = 0; $k4 = 0;
$file = new FileHandle ("$infile") || die("Cannot open miRNA file: $infile\n");
while(<$file>)
{
```

```
 $_=~s/^[\s|\t]+//g;
 $_=~s/[\s|\t|\r|\n]+$//g;
 if ($_ =~/^>(\S+)/) {
  $i = $1;
  $gnm{$i}{"Seq"} = "";
  $k1++;
 } else {
  $gnm{$i}{"Seq"} .= uc($_);
 }
}
close $file || die;

print STDERR "\nNow = [",sub_format_datetime(localtime(time())),"]\tLoad file: $infile OK\t$k1\n\n";

foreach $i (keys %gnm) {
 $j = $gnm{$i}{"Seq"};
 $matureSeq{$j}{$i} = 0;
 if ($inclusion >= 2) {
  for ($k1 = 0; $k1 <= $start_len ;$k1++) {
   $matureSeq{substr($j,0,length($j)-$k1)}{$i} = $k1;
  }
 }
}
############################################### read soap result file ##########################################
$i = 0; $k3 = 0; $m = 0; my $j2 = ""; my $j3 = "";
if ($soap_result ne "") {
 if ($soap_result =~/^(.+\/)([^\/]*)\*([^\*]+)$/) {        # data/a*b data/*b
  $k = $3; $j = $1; $j2 = $2;print STDERR "1.j2=$j2,k=$k,j=$j\n"; # $k = b; $j = data/
 } elsif ($soap_result =~/^([^\/]*)\*([^\*]+)$/) {        # a*b, *b
  $k = $2; $j = "./"; $j2 = $1;print STDERR "2.j2=$j2,k=$k,j=$j\n"; # $k = b; $j = ./
 } elsif ($soap_result =~/^(.+\/)([^\/]+)$/) {        # data/a
  $k = $2; $j = $1; $j2 = "";print STDERR "3.j2=$j2,k=$k,j=$j\n"; # $k = a; $j = data/
 } elsif ($soap_result =~/^([^\*]+)$/) {          # a
  $k = $1; $j = "./"; $j2 = "";print STDERR "4.j2=$j2,k=$k,j=$j\n"; # $k = a; $j = ./
 } else {
  print STDERR $soap_result;
  die("reinput $soap_result!\n");
 }
 $k = quotemeta($k); if ($j2 ne "") {$j2 = quotemeta($j2)};
 opendir(FDIR, $j) || die("Can not open dir: $j\n");
 foreach $file (sort readdir(FDIR)) {
# }
# while ($file=readdir(FDIR)) {
  if ($file=~/$j2(.*)$k$/) {
   if ($1 ne "") {
    $num{$i}->[0] = $1;
 #   print STDERR "$i=$1, num=";
   } else {
    $num{$i}->[0] = $i;
 #   print STDERR "$i=$1";
   }
   $num{$i}->[1] = 0;
   $bfile = $j ."/$file";
   print STDERR "\n\tthe $i file lib $num{$i}->[0]:\t$bfile\n";

#-----------------------------------------------------------------------------------------------
$k1 = 0; $b = ""; $a = ""; my %nam;
$file = new FileHandle ("$bfile") || die("Cannot open miRNA file: $bfile\n");
while(<$file>)
{
# $_=~s/^[\s|\t]+//g;
# $_=~s/[\s|\t|\r|\n]+$//g;
 if ($_ =~/^(\S+)\s+/) {
  $a = $1;
  if (!exists($nam{$a})) {
#   $a =~/^(\S+)\_(\S+)\_(\d+)x?/;
#   $num{$i}->[1] += $3;
#   $k1++;
   if ($a =~/^(\S+)\_(\S+)\_(\d+)x/) {
    $num{$i}->[1] += $3;
    $k1++;
   } else {
    $num{$i}->[1] += 1;
    $k1++;
   }
```

```
 }
 } else {
  print STDERR "wrong format $_\n";
 }
}
undef(%nam);
close $file || die;
print "Soap $1,\tnum=",$num{$i}->[1]; print STDERR "\tSoap $i, $1,\tnum=",$num{$i}->[1];
$num{$i}->[1] = 1.0*$num{$i}->[1]/$normal_base;
print "\t normalize=",$num{$i}->[1],"\n"; print STDERR "\t normalize=",$num{$i}->[1];

print STDERR "\n\tLoad file: $bfile OK\t$k1\n";
 $k3+=$k1;
#-----------------------------------------------------------------------------------------------
  $i++; #print STDERR "i=$i\n"
 }

 }
 closedir(FDIR);
print STDERR "\nNow = [",sub_format_datetime(localtime(time())),"]\tLoad all soap result files OK. $i,$k1\n\n";
}

############################################### read library ###########################################
$i = 0; $k3 = 0; $m = 0; my $j2 = ""; my $j3 = "";
if ($library ne "") {
 if ($library =~/^(.+\/)([^\/]*)\*([^\*]+)$/) {       # data/a*b data/*b
  $k = $3; $j = $1; $j2 = $2;print STDERR "1.j2=$j2,k=$k,j=$j\n"; # $k = b; $j = data/
 } elsif ($soap_result =~/^([^\/]*)\*([^\*]+)$/) {        # a*b, *b
  $k = $2; $j = "./"; $j2 = $1;print STDERR "2.j2=$j2,k=$k,j=$j\n"; # $k = b; $j = ./
 } elsif ($soap_result =~/^(.+\/)([^\/]+)$/) {         # data/a
  $k = $2; $j = $1; $j2 = "";print STDERR "3.j2=$j2,k=$k,j=$j\n"; # $k = a; $j = data/
 } elsif ($soap_result =~/^([^\*]+)$/) {          # a
  $k = $1; $j = "./"; $j2 = "";print STDERR "4.j2=$j2,k=$k,j=$j\n"; # $k = a; $j = ./
 } else {
  print STDERR $library;
  die("reinput $library!\n");
 }
 $k = quotemeta($k); if ($j2 ne "") {$j2 = quotemeta($j2)};
 opendir(FDIR, $j) || die("Can not open dir: $j\n");
 foreach $file (sort readdir(FDIR)) {
# }
# while ($file=readdir(FDIR)) {
  if ($file=~/$j2(.*)$k$/) {
   if ($1 ne "") {
    $num{$i}->[0] = $1;
 #   print STDERR "$i=$1, num=";
   } else {
    $num{$i}->[0] = $i;
 #   print STDERR "$i=$1";
   }
   $num{$i}->[1] = 0;
   $bfile = $j ."/$file";
   print STDERR "\n\tthe $i file lib $num{$i}->[0]:\t$bfile\n";

#-----------------------------------------------------------------------------------------------
$k1 = 0; $b = ""; $a = ""; my %nam;
$file = new FileHandle ("$bfile") || die("Cannot open miRNA file: $bfile\n");
while(<$file>)
{
# $_=~s/^[\s|\t]+//g;
# $_=~s/[\s|\t|\r|\n]+$//g;
 if ($_ =~/^>(\S+)\s+/) {
  $a = $1;
  if (!exists($nam{$a})) {
   if ($a =~/^(\S+)\_(\S+)\_(\d+)x/) {
    $num{$i}->[1] += $3;
    $k1++;
   } else {
    $num{$i}->[1] += 1;
    $k1++;
   }
  }
 } else {
#  print STDERR "wrong format $_\n";
 }
```

```
}
undef(%nam);
close $file || die;
print "Soap $1,\tnum=",$num{$i}->[1]; print STDERR "\tSoap $i, $1,\tnum=",$num{$i}->[1];
$num{$i}->[1] = 1.0*$num{$i}->[1]/$normal_base;
print "\t normalize=",$num{$i}->[1],"\n"; print STDERR "\t normalize=",$num{$i}->[1];

print STDERR "\n\tLoad file: $bfile OK\t$k1\n";
 $k3+=$k1;
#----------------------------------------------------------------------------------------------------------
   $i++; #print STDERR "i=$i\n"
  }

 }
 closedir(FDIR);
print STDERR "\nNow = [",sub_format_datetime(localtime(time())),"]\tLoad all library OK. $i,$k1\n\n";
}


#sub_end_program();
################################################ read file ############################################################
$i = 0; $k3 = 0; $m = 0; my $ss1;
if ($libFile ne "") {
 if ($libFile =~/^(.+\/)([^\/]*)\*([^\*]+)$/) {       # data/a*b data/*b
  $k = $3; $j = $1; $j2 = $2;print STDERR "1.j2=$j2,k=$k,j=$j\n"; # $k = b; $j = data/
 } elsif ($libFile =~/^([^\/]*)\*([^\*]+)$/) {       # a*b, *b
  $k = $2; $j = "./"; $j2 = $1;print STDERR "2.j2=$j2,k=$k,j=$j\n"; # $k = b; $j = ./
 } elsif ($libFile =~/^(.+\/)([^\/]+)$/) {        # data/a
  $k = $2; $j = $1; $j2 = "";print STDERR "3.j2=$j2,k=$k,j=$j\n"; # $k = a; $j = data/
 } elsif ($libFile =~/^([^\*]+)$/) {          # a
  $k = $1; $j = "./"; $j2 = "";print STDERR "4.j2=$j2,k=$k,j=$j\n"; # $k = a; $j = ./
 } else {
  print STDERR $libFile;
  die("reinput $libFile!\n");
 }
 $k = quotemeta($k); if ($j2 ne "") {$j2 = quotemeta($j2)};
 opendir(FDIR, $j) || die("Can not open dir: $j\n");
 foreach $file (sort readdir(FDIR)) {
# }
# while ($file=readdir(FDIR)) {
  if ($file=~/$j2(.*)$k$/) {
   if ($1 ne "") {
    $seg{$i} = $1; # number ==> name
   } else {
    $seg{$i} = $i; # number ==> number
   }
   $bfile = $j ."/$file";
   print STDERR "\n\tthe $i file lib $seg{$i}:\t$bfile\n";


   foreach $key (keys %gnm) {
    if (!exists($gnm{$key}{$i})) {
     if ($inclusion == 2) {
      $gnm{$key}{$i}{0} = 0; # match
      $gnm{$key}{$i}{1} = 0; # truncation
      $gnm{$key}{$i}{2} = 0; # tailing
      $gnm{$key}{$i}{3} = 0; # truncation and tailing
     } elsif ($inclusion > 2) {
      for ($k4 = 0; $k4 <= $start_len ;$k4++) {
       for ($n = 0; $n <= $start_len ;$n++) {
        $gnm{$key}{$i}{$k4."_".$n} = 0;
       }
      }
     } else {
      $gnm{$key}{$i} = 0;
      $gnm{$key}{",$i"} = 0;
     }

    } else {
     print STDERR "lib name is wrong:$key,$i!\n";
    }
   }
#----------------------------------------------------------------------------------------------------------
$k1 = 0; $b = ""; $a = ""; my $c = 0;
$file = new FileHandle ("$bfile") || die("Cannot open miRNA file: $bfile\n");
```

```
while(<$file>)
{
 $_=~s/^[\s|\t]+//g;
 $_=~s/[\s|\t|\r|\n]+$//g;
 if ($_ =~/^>(\S+)\_(\S+)\_(\d+)x/) {
  $a = $c; $c = $3;
  if ($b ne "") {
   my $b1 = reverseDNAString($b);
   if ($inclusion == 0) {
    if (exists($matureSeq{$b})) {
     foreach $key (keys %{$matureSeq{$b}}) {
      $gnm{$key}{$i} += $a;
     }
    }
    if (exists($matureSeq{$b1})) {
     foreach $key (keys %{$matureSeq{$b1}}) {
      $gnm{$key}{",$i"} += $a;
     }
    }
#   foreach $key (keys %gnm) {
# #   if ($b=~/$gnm{$key}{"Seq"}/i || $gnm{$key}{"Seq"} =~ /$b/i) {
#    if ($b eq $gnm{$key}{"Seq"}) {
#     $gnm{$key}{$i} += $a;
# #   } elsif ($b1=~/$gnm{$key}{"Seq"}/i || $gnm{$key}{"Seq"} =~ /$b1/i) {
#    } elsif ($b1 eq $gnm{$key}{"Seq"}) {
#     $gnm{$key}{",$i"} += $a;
#    }
#   }
   } elsif ($inclusion == 1) {
    foreach $key (keys %gnm) {
     if ($b=~/$gnm{$key}{"Seq"}/i || $gnm{$key}{"Seq"} =~ /$b/i) {
      $gnm{$key}{$i} += $a;
     } elsif ($b1=~/$gnm{$key}{"Seq"}/i || $gnm{$key}{"Seq"} =~ /$b1/i) {
      $gnm{$key}{",$i"} += $a;
     }
    }
   } elsif ($inclusion == 2) {
    my %tmpkey=();
    for ($k4 = 0; $k4 <=$start_len ;$k4++) {
     $n = substr($b,0,length($b)-$k4);
     if (exists($matureSeq{$n})) {
      foreach $key (keys %{$matureSeq{$n}}) {
       if (exists($tmpkey{$key})) {
        next;
       } else {
        $tmpkey{$key} = 1;
       }
    #   print STDERR "$b\t$a\n";
       if ($matureSeq{$n}{$key}==0 && $k4 ==0 ) {
        $gnm{$key}{$i}{0} += $a;
       } elsif ($matureSeq{$n}{$key}>0 && $k4 ==0 ) {
        $gnm{$key}{$i}{1} += $a;
       } elsif ($matureSeq{$n}{$key}==0 && $k4 > 0 ) {
        $gnm{$key}{$i}{2} += $a;
       } elsif ($matureSeq{$n}{$key}>0 && $k4 >0 ) {
        $gnm{$key}{$i}{3} += $a;
       }
      }
     }
    }
   } else {
    my %tmpkey=();
    for ($k4 = 0; $k4 <=$start_len ;$k4++) {
     $n = substr($b,0,length($b)-$k4);
     if (exists($matureSeq{$n})) {
      foreach $key (keys %{$matureSeq{$n}}) {
       if (exists($tmpkey{$key})) {
        next;
       } else {
        $tmpkey{$key} = 1;
       }
       $gnm{$key}{$i}{$matureSeq{$n}{$key}."_".$k4} += $a; # truncation _ tail
      }
     }
    }
```

```
   }
  }
  $b = "";
  $k1++; # $k1%10000 != 0 || print STDERR "$k1,";
 } elsif ($_=~/^>/) {
  $a = $c; $c = 1;
  if ($b ne "") {
   my $b1 = reverseDNAString($b);
   if ($inclusion == 0) {
    if (exists($matureSeq{$b})) {
     foreach $key (keys %{$matureSeq{$b}}) {
      $gnm{$key}{$i} += $a;
     }
    }
    if (exists($matureSeq{$b1})) {
     foreach $key (keys %{$matureSeq{$b1}}) {
      $gnm{$key}{",$i"} += $a;
     }
    }
   } elsif ($inclusion == 1) {
    foreach $key (keys %gnm) {
     if ($b=~/$gnm{$key}{"Seq"}/i || $gnm{$key}{"Seq"} =~ /$b/i) {
      $gnm{$key}{$i} += $a;
     } elsif ($b1=~/$gnm{$key}{"Seq"}/i || $gnm{$key}{"Seq"} =~ /$b1/i) {
      $gnm{$key}{",$i"} += $a;
     }
    }
   } elsif ($inclusion == 2) {
    my %tmpkey=();
    for ($k4 = 0; $k4 <=$start_len ;$k4++) {
     $n = substr($b,0,length($b)-$k4);
     if (exists($matureSeq{$n})) {
      foreach $key (keys %{$matureSeq{$n}}) {
       if (exists($tmpkey{$key})) {
        next;
       } else {
        $tmpkey{$key} = 1;
       }
       if ($matureSeq{$n}{$key}==0 && $k4 ==0 ) {
        $gnm{$key}{$i}{0} += $a;
       } elsif ($matureSeq{$n}{$key}>0 && $k4 ==0 ) {
        $gnm{$key}{$i}{1} += $a;
       } elsif ($matureSeq{$n}{$key}==0 && $k4 > 0 ) {
        $gnm{$key}{$i}{2} += $a;
       } elsif ($matureSeq{$n}{$key}>0 && $k4 >0 ) {
        $gnm{$key}{$i}{3} += $a;
       }
      }
     }
    }
   } else {
    my %tmpkey=();
    for ($k4 = 0; $k4 <=$start_len ;$k4++) {
     $n = substr($b,0,length($b)-$k4);
     if (exists($matureSeq{$n})) {
      foreach $key (keys %{$matureSeq{$n}}) {
       if (exists($tmpkey{$key})) {
        next;
       } else {
        $tmpkey{$key} = 1;
       }
       $gnm{$key}{$i}{$matureSeq{$n}{$key}."_".$k4} += $a; # truncation _ tail
      }
     }
    }
   }
  }
  $b = "";
  $k1++; # $k1%10000 != 0 || print STDERR "$k1,";
 }else {
  $b .= uc($_);
 }
}
$a = $c;
if ($b ne "") {
```

```
 my $b1 = reverseDNAString($b);
# foreach $key (keys %gnm) {
# # if ($b=~/$gnm{$key}{"Seq"}/i || $gnm{$key}{"Seq"} =~ /$b/i) {
#  if ($b eq $gnm{$key}{"Seq"}) {
#   $gnm{$key}{$i} += $a;
# # } elsif ($b1=~/$gnm{$key}{"Seq"}/i || $gnm{$key}{"Seq"} =~ /$b1/i) {
#  } elsif ($b1 eq $gnm{$key}{"Seq"}) {
#   $gnm{$key}{",$i"} += $a;
#  }
# }
 if ($inclusion == 0) {
  if (exists($matureSeq{$b})) {
   foreach $key (keys %{$matureSeq{$b}}) {
    $gnm{$key}{$i} += $a;
   }
  }
  if (exists($matureSeq{$b1})) {
   foreach $key (keys %{$matureSeq{$b1}}) {
    $gnm{$key}{",$i"} += $a;
   }
  }
#  foreach $key (keys %gnm) {
#  # if ($b=~/$gnm{$key}{"Seq"}/i || $gnm{$key}{"Seq"} =~ /$b/i) {
#   if ($b eq $gnm{$key}{"Seq"}) {
#    $gnm{$key}{$i} += $a;
#  # } elsif ($b1=~/$gnm{$key}{"Seq"}/i || $gnm{$key}{"Seq"} =~ /$b1/i) {
#   } elsif ($b1 eq $gnm{$key}{"Seq"}) {
#    $gnm{$key}{",$i"} += $a;
#   }
#  }
 } elsif ($inclusion == 1) {
  foreach $key (keys %gnm) {
   if ($b=~/$gnm{$key}{"Seq"}/i || $gnm{$key}{"Seq"} =~ /$b/i) {
    $gnm{$key}{$i} += $a;
   } elsif ($b1=~/$gnm{$key}{"Seq"}/i || $gnm{$key}{"Seq"} =~ /$b1/i) {
    $gnm{$key}{",$i"} += $a;
   }
  }
 } elsif ($inclusion == 2) {
  my %tmpkey=();
  for ($k4 = 0; $k4 <=$start_len ;$k4++) {
   $n = substr($b,0,length($b)-$k4);
   if (exists($matureSeq{$n})) {
    foreach $key (keys %{$matureSeq{$n}}) {
     if (exists($tmpkey{$key})) {
      next;
     } else {
      $tmpkey{$key} = 1;
     }
     if ($matureSeq{$n}{$key}==0 && $k4 ==0 ) {
      $gnm{$key}{$i}{0} += $a;
     } elsif ($matureSeq{$n}{$key}>0 && $k4 ==0 ) {
      $gnm{$key}{$i}{1} += $a;
     } elsif ($matureSeq{$n}{$key}==0 && $k4 > 0 ) {
      $gnm{$key}{$i}{2} += $a;
     } elsif ($matureSeq{$n}{$key}>0 && $k4 >0 ) {
      $gnm{$key}{$i}{3} += $a;
     }
    }
   }
  }
 } else {
  my %tmpkey=();
  for ($k4 = 0; $k4 <=$start_len ;$k4++) {
   $n = substr($b,0,length($b)-$k4);
   if (exists($matureSeq{$n})) {
    foreach $key (keys %{$matureSeq{$n}}) {
     if (exists($tmpkey{$key})) {
      next;
     } else {
      $tmpkey{$key} = 1;
     }
     $gnm{$key}{$i}{$matureSeq{$n}{$key}."_".$k4} += $a; # truncation _ tail
    }
   }
```

```
   }
  }
 }
close $file || die;

print STDERR "\tLoad file: $bfile OK\t$k1\n";
 $k3+=$k1;
#--------------------------------------------------------------------------------------------------
   $i++;
  }

 }
 closedir(FDIR);
print STDERR "\nNow = [",sub_format_datetime(localtime(time())),"]\tLoad all lib files: $i,$k1\n";
}

############################################## output ######################################################
print "Name\tLength"; my %libst=(); $i = 0;
foreach $key (sort numerically keys %seg) {
 if (exists($libst{$seg{$key}})) {
  $i = 1;
 } else {
  $libst{$seg{$key}} = $key;# name ==> number
 }
}
$i = 1;
if ($i == 0) {
 print STDERR "\nLib:";
 foreach $key (sort keys %libst) {
  if ($opformat == 0) {
   print "\tRaw_$key\tRaw_$key*";
#   print STDERR "\tRaw_$key\tRaw*";
  } elsif ($opformat == 1) {
   print "\tRaw_",$key;
#   print STDERR "\tRaw_",$key;
  } elsif ($opformat == 2) {
   if ($inclusion == 2) {
    print "\t$key\t$key\_Tr\t$key\_Ta\t$key\_T&T";
   } else {
    print "\t$key+";
   }
#   print STDERR "\tRaw_$key+";
  } elsif ($opformat == 3) {
   print "\tRaw_$key-";
#   print STDERR "\tRaw_$key-";
  }
 }
 foreach $key (sort keys %libst) {
  if ($opformat == 0) {
   print "\t",$key,"\t$key*";
   print STDERR "\t",$key,"\t*";
  } elsif ($opformat == 1) {
   print "\t",$key;
   print STDERR "\t",$key;
  } elsif ($opformat == 2) {
   if ($inclusion == 2) {
    print "\t$key\t$key\_Tr\t$key\_Ta\t$key\_T&T";
   } else {
    print "\t$key+";
   }
   print STDERR "\t$key+";
  } elsif ($opformat == 3) {
   print "\t$key-";
   print STDERR "\t$key-";
  }
 }
 print "\n"; print STDERR "\n";
 if ($soap_result ne "" || $library ne "" || $lib_size ne "") {
  $j2 = 0;
  print STDERR "\nSoap:";
  foreach $key (sort numerically keys %num) {
   print STDERR "\t$num{$key}->[0]\t$key";
   if ($normal_base > 1) {
    $num{$key}->[1] = 1.0*$num{$key}->[1];#/$normal_base;
   }
```

```
 $buf[$j2] = $j2;# print STDERR "buf[$j2]=$buf[$j2]\t";
 $j2++;
}
if ($ask_input == 1) {
 print STDERR "\n\n1.please input the num in soap result: e.g. 3,2,0,1; \[0,1,2,3\]\n";
 $ss1 = <STDIN>; $ss1 =~s/[\s|\t|\r|\n]+$//g;
 if ($ss1 ne "") {
  @buf=split(/\,/,$ss1);
 }
}
foreach $key (sort keys %gnm) {
 print $key,"\t",length($gnm{$key}{"Seq"}); $j2 = 0;
 foreach $k1 (sort keys %libst) {
  if ($opformat == 0) {
   print "\t",$gnm{$key}{$libst{$k1}},"\t",$gnm{$key}{",$libst{$k1}"};
  } elsif ($opformat == 1) {
   print "\t",$gnm{$key}{$libst{$k1}}+$gnm{$key}{",$libst{$k1}"};
  } elsif ($opformat == 2) {
   if ($inclusion < 2) {
    print "\t",$gnm{$key}{$libst{$k1}};
   } elsif ($inclusion == 2) {
    print "\t",$gnm{$key}{$libst{$k1}}{0},"\t",$gnm{$key}{$libst{$k1}}{1},"\t",
     $gnm{$key}{$libst{$k1}}{2},"\t",$gnm{$key}{$libst{$k1}}{3};
   } else {
    print "\t";
    for ($k4 = 0; $k4 <= $start_len ;$k4++) {
     for ($n = 0; $n <= $start_len ;$n++) {
      print ";",$gnm{$key}{$libst{$k1}}{$k4."_".$n};
     }
    }
   }
  } elsif ($opformat == 3) {
   print "\t",$gnm{$key}{",$libst{$k1}"};
  }
 }
 foreach $k1 (sort keys %libst) {
  if ($opformat == 0) {
   printf("\t%.1f\t%.1f",$gnm{$key}{$libst{$k1}}/$num{$buf[$j2]}->[1],$gnm{$key}{",$libst{$k1}"}/$num{$buf[$j2]}->[1]);
  } elsif ($opformat == 1) {
   printf("\t%.1f",$gnm{$key}{$libst{$k1}}/$num{$buf[$j2]}->[1]+$gnm{$key}{",$libst{$k1}"}/$num{$buf[$j2]}->[1]);
  } elsif ($opformat == 2) {
   # printf("\t%.1f",$gnm{$key}{$libst{$k1}}/$num{$buf[$j2]}->[1]);
   if ($inclusion < 2) {
   # print "\t",$gnm{$key}{$libst{$k1}};
    printf("\t%.1f",$gnm{$key}{$libst{$k1}}/$num{$buf[$j2]}->[1]);
   } elsif ($inclusion == 2) {
    printf("\t%.1f\t%.1f\t%.1f\t%.1f",$gnm{$key}{$libst{$k1}}{0}/$num{$buf[$j2]}->[1],$gnm{$key}{$libst{$k1}}{1}/$num{$buf[$j2]}->[1],
     $gnm{$key}{$libst{$k1}}{2}/$num{$buf[$j2]}->[1],$gnm{$key}{$libst{$k1}}{3}/$num{$buf[$j2]}->[1]);
#   print "\t",$gnm{$key}{$libst{$k1}}{0}"\t",$gnm{$key}{$libst{$k1}}{1}"\t",$gnm{$key}{$libst{$k1}}{2}"\t",$gnm{$key}{$libst{$k1}}{3};
   } else {
    $m = "\t";
    for ($k4 = 0; $k4 <= $start_len ;$k4++) {
     for ($n = 0; $n <= $start_len ;$n++) {
      $m.= ";",$gnm{$key}{$libst{$k1}}{$k4."_".$n}/$num{$buf[$j2]}->[1];
     }
    }
    print $m;
   }
  } elsif ($opformat == 3) {
   printf("\t%.1f",$gnm{$key}{",$libst{$k1}"}/$num{$buf[$j2]}->[1]);
  }
  $j2++;
 }
 print "\n";
}
} else {
foreach $key (sort keys %gnm) {
 print $key,"\t",length($gnm{$key}{"Seq"});
 foreach $k1 (sort keys %libst) {
  if ($opformat == 0) {
   print "\t",$gnm{$key}{$libst{$k1}},"\t",$gnm{$key}{",$libst{$k1}"};
  } elsif ($opformat == 1) {
   print "\t",$gnm{$key}{$libst{$k1}}+$gnm{$key}{",$libst{$k1}"};
  } elsif ($opformat == 2) {
  # print "\t",$gnm{$key}{$libst{$k1}};
```

```
    if ($inclusion < 2) {
     print "\t",$gnm{$key}{$libst{$k1}};
    } elsif ($inclusion == 2) {
     print "\t",$gnm{$key}{$libst{$k1}}{0},"\t",$gnm{$key}{$libst{$k1}}{1},"\t",
      $gnm{$key}{$libst{$k1}}{2},"\t",$gnm{$key}{$libst{$k1}}{3};
    } else {
     print "\t";
     for ($k4 = 0; $k4 <= $start_len ;$k4++) {
      for ($n = 0; $n <= $start_len ;$n++) {
       print ";",$gnm{$key}{$libst{$k1}}{$k4."_".$n};
      }
     }
    }
   } elsif ($opformat == 3) {
    print "\t",$gnm{$key}{",$libst{$k1}"};
   }
  }
  print "\n";
 }
}
} else {
 foreach $key (sort numerically keys %seg) {
  if ($opformat == 0) {
   print "\tRaw_$seg{$key}\tRaw*";
#   print STDERR "\tRaw_$seg{$key}\tRaw*";
  } elsif ($opformat == 1) {
   print "\tRaw_",$seg{$key};
#   print STDERR "\tRaw_",$seg{$key};
  } elsif ($opformat == 2) {
  # print "\tRaw_$seg{$key}+";
   if ($inclusion == 2) {
    print "\t$seg{$key}\t$seg{$key}\_Tr\t$seg{$key}\_Ta\t$seg{$key}\_T&T";
   } else {
    print "\t$seg{$key}";
   }
#   print STDERR "\tRaw_$seg{$key}+";
  } elsif ($opformat == 3) {
   print "\tRaw_$seg{$key}-";
#   print STDERR "\tRaw_$seg{$key}-";
  }
 }
 foreach $key (sort numerically keys %seg) {
  if ($opformat == 0) {
   print "\t",$seg{$key},"\t*";
   print STDERR "\t",$seg{$key},"\t*";
  } elsif ($opformat == 1) {
   print "\t",$seg{$key};
   print STDERR "\t",$seg{$key};
  } elsif ($opformat == 2) {
   if ($inclusion == 2) {
    print "\t$seg{$key}\t$seg{$key}\_Tr\t$seg{$key}\_Ta\t$seg{$key}\_T&T";
   } else {
    print "\t$seg{$key}";
   }
  # print "\t$seg{$key}+";
  # print STDERR "\t$seg{$key}+";
  } elsif ($opformat == 3) {
   print "\t$seg{$key}-";
   print STDERR "\t$seg{$key}-";
  }
 }
 print "\n"; print STDERR "\n";
 if ($soap_result ne "" || $library ne "" || $lib_size ne "") {
  $j2 = 0;
#  for (;$j2 < 4 ;$j2++) {
#   $buf[$j2] = $j2;
#  }
  foreach $key (sort numerically keys %num) {
   print STDERR "\t$key\t",$num{$key}->[0];
   if ($normal_base > 1) {
    $num{$key}->[1] = 1.0*$num{$key}->[1];#/$normal_base;
   }
   $buf[$j2] = $j2; #print STDERR "buf[$j2]=$buf[$j2]\t";
   $j2++;
  }
```

```
 if ($ask_input == 1) {
  print STDERR "\n2.please input the num in soap result: e.g. 3,2,0,1; [0,1,2,3]\n";
  $ss1 = <STDIN>; $ss1 =~s/[\s|\t|\r|\n]+$//g;
  if ($ss1 ne "") {
#   @buf=split(/\,/,$ss1);
  }
 }

 foreach $key (sort keys %gnm) {
  print $key,"\t",length($gnm{$key}{"Seq"}); $j2 = 0;
  foreach $k1 (sort numerically keys %seg) {
   if ($opformat == 0) {
    print "\t",$gnm{$key}{$k1},"\t",$gnm{$key}{",$k1"};
   } elsif ($opformat == 1) {
    print "\t",$gnm{$key}{$k1}+$gnm{$key}{",$k1"};
   } elsif ($opformat == 2) {
    #print "\t",$gnm{$key}{$k1};
    if ($inclusion < 2) {
     print "\t",$gnm{$key}{$k1};
    } elsif ($inclusion == 2) {
     print "\t",$gnm{$key}{$k1}{0},"\t",$gnm{$key}{$k1}{1},"\t",$gnm{$key}{$k1}{2},"\t",$gnm{$key}{$k1}{3};
    } else {
     print "\t";
     for ($k4 = 0; $k4 <= $start_len ;$k4++) {
      for ($n = 0; $n <= $start_len ;$n++) {
       print ";",$gnm{$key}{$k1}{$k4."_".$n};
      }
     }
    }
   } elsif ($opformat == 3) {
    print "\t",$gnm{$key}{",$k1"};
   }
  }
  foreach $k1 (sort numerically keys %seg) {
   if ($opformat == 0) {
    printf("\t%.1f\t%.1f",$gnm{$key}{$k1}/$num{$buf[$j2]}->[1],$gnm{$key}{",$k1"}/$num{$buf[$j2]}->[1]);
   } elsif ($opformat == 1) {
    printf("\t%.1f",$gnm{$key}{$k1}/$num{$buf[$j2]}->[1]+$gnm{$key}{",$k1"}/$num{$buf[$j2]}->[1]);
   } elsif ($opformat == 2) {
    #printf("\t%.1f",$gnm{$key}{$k1}/$num{$buf[$j2]}->[1]);
    if ($inclusion < 2) {
    # print "\t",$gnm{$key}{$k1};
     printf("\t%.1f",$gnm{$key}{$k1}/$num{$buf[$j2]}->[1]);
    } elsif ($inclusion == 2) {
     printf("\t%.1f\t%.1f\t%.1f\t%.1f",$gnm{$key}{$k1}{0}/$num{$buf[$j2]}->[1],$gnm{$key}{$k1}{1}/$num{$buf[$j2]}->[1],
      $gnm{$key}{$k1}{2}/$num{$buf[$j2]}->[1],$gnm{$key}{$k1}{3}/$num{$buf[$j2]}->[1]);
 #   print "\t",$gnm{$key}{$k1}{0}"\t",$gnm{$key}{$k1}{1}"\t",$gnm{$key}{$k1}{2}"\t",$gnm{$key}{$k1}{3};
    } else {
     $m = "\t";
     for ($k4 = 0; $k4 <= $start_len ;$k4++) {
      for ($n = 0; $n <= $start_len ;$n++) {
       $m.= ";",$gnm{$key}{$k1}{$k4."_".$n}/$num{$buf[$j2]}->[1];
      }
     }
     print $m;
    }
   } elsif ($opformat == 3) {
    printf("\t%.1f",$gnm{$key}{",$k1"}/$num{$buf[$j2]}->[1]);
   }
   $j2++;
  }
  print "\n";
 }
} else {
 foreach $key (sort keys %gnm) {
  print $key,"\t",length($gnm{$key}{"Seq"});
  foreach $k1 (sort numerically keys %seg) {
   if ($opformat == 0) {
    print "\t",$gnm{$key}{$k1},"\t",$gnm{$key}{",$k1"};
   } elsif ($opformat == 1) {
    print "\t",$gnm{$key}{$k1}+$gnm{$key}{",$k1"};
   } elsif ($opformat == 2) {
   # print "\t",$gnm{$key}{$k1};
    if ($inclusion < 2) {
     print "\t",$gnm{$key}{$k1};
```

```
    } elsif ($inclusion == 2) {
     print "\t",$gnm{$key}{$k1}{0},"\t",$gnm{$key}{$k1}{1},"\t",$gnm{$key}{$k1}{2},"\t",$gnm{$key}{$k1}{3};
    } else {
     print "\t";
     for ($k4 = 0; $k4 <= $start_len ;$k4++) {
      for ($n = 0; $n <= $start_len ;$n++) {
       print ";",$gnm{$key}{$k1}{$k4."_".$n};
      }
     }
    }
   } elsif ($opformat == 3) {
    print "\t",$gnm{$key}{",$k1"};
   }
  }
  print "\n";
 }
 }
}
sub_end_program();


################################################################################################################
####################################                    ################################
####################################      "main end"      ################################
####################################                    ################################
################################################################################################################


sub usage
{
 print "Program :\t$0\n";
 print "Version :\t$version\n";
 print "Author  :\tLei Gao, UC,Riverside\n";
 print "Contact :\tLei Gao <highlei\@gmail.com>\n";
 print "\nUsage: $0 [options]\n";
 print "\t-i <str> input the miRNA file.";
 print " eg: maize10.2cn20.-CDS.1.all.mature.fa\n";
 print "\t-d <str> input the library file(s).";
 print " eg: \"data/zea.*fa\"\n";
 print "\t-S <str> input soap result file(s).";
 print " eg: \"data/zea.*soap\"\n";
 print "\t-N <int> input the base for normalization.";
 print " [$normal_base]\n";
 print "\t-s <int> the library size.";
 print " [$lib_size]\n";
 print "\t-L <str> input library.";
 print " [$library]\n";
 print "\t-O <int> output format (0: +,-; 1:+ plus -; 2:+; 3:-).";
 print " [$opformat]\n";
 print "\t-I <int> 0: match; 1: include; 2: 4kind Truncation&Tailing; 3: all T&T.";
 print " [$inclusion]\n";
 print "\t-A <int> ask iput (1) or not (0).";
 print " [$ask_input]\n";
 print "\t-l <int> the max truncation length.";
 print " [$start_len]\n";
# print "\t-c <int> the score is allowed.";
# print " [$score]\n";
 print "\n\t-h display this help\n";
# print "  Note: please add quotation mark, if you input parameter in command line!\n";
 print "\nExample:\n";
 print "$0 -i maize10.2cn20.-CDS.1.all.mature.fa -d \"data/zea.*fa\"\n";
 print ("==========================| $0  end  |==================================\n\n");

   exit(0);
}
###############################################################################################################
######################                sub_format_datetime
###############################################################################################################

sub sub_format_datetime #时间子程序
{
   my ($sec, $min, $hour, $day, $mon, $year, $wday, $yday, $isdst) = @_;
   sprintf("%4d-%02d-%02d %02d:%02d:%02d", $year+1900, $mon+1, $day, $hour, $min, $sec);
}
```

```
####################################################################################################
######################                  sub_end_program
####################################################################################################
sub sub_end_program
{
 print STDERR ("\n............................................................\n");
 my $Time_End = sub_format_datetime(localtime(time()));
 print STDERR "Running from [$Time_Start] to [$Time_End]\n";
 $end = time();
 printf STDERR ("Total execute time : %.2f s\n",$end-$start);
 print STDERR ("==========================| $0  end  |==================================\n\n");
 exit(0);

}
####################################################################################################
######################                  reverseDNAString
####################################################################################################

sub reverseDNAString
{
 my($rdstr) = @_;
 my ($sr1,$sr2);
 $rdstr = reverse($rdstr);
 $rdstr =~tr/ACGTRYMKacgtrymk/TGCAYRKMtgcayrkm/;
 return $rdstr;
}
```

For manuscripts utilizing custom algorithms or software that are central to the research but not yet described in published literature, software must be made available to editors/reviewers upon request. We strongly encourage code deposition in a community repository (e.g. GitHub). See the Nature Research guidelines for submitting code & software for further information.

Data

Policy information about availability of data

All manuscripts must include a data availability statement. This statement should provide the following information, where applicable:

- Accession codes, unique identifiers, or web links for publicly available datasets
- A list of figures that have associated raw data
- A description of any restrictions on data availability

All raw data and processed data were deposited in NCBI GEO (http://www.ncbi.nlm.nih.gov/geo/) with the accession number GSE111240.

Field-specific reporting

Please select the best fit for your research. If you are not sure, read the appropriate sections before making your selection.

☒ Life sciences ☐ Behavioural & social sciences ☐ Ecological, evolutionary & environmental sciences

For a reference copy of the document with all sections, see nature.com/authors/policies/ReportingSummary-flat.pdf

Life sciences study design

All studies must disclose on these points even when the disclosure is negative.

Sample size	15-day-old Arabidopsis seedlings, as the whole seedlings are suitable for a set of experiments.
Data exclusions	No data were excluded from the analyses.
Replication	Three biological replicates were used in this study.
Randomization	This is not relevant to this study.
Blinding	This is not relevant to this study.

Reporting for specific materials, systems and methods

Materials & experimental systems

n/a	Involved in the study
☐	☒ Unique biological materials
☐	☒ Antibodies
☒	☐ Eukaryotic cell lines
☒	☐ Palaeontology
☒	☐ Animals and other organisms
☒	☐ Human research participants

Methods

n/a	Involved in the study
☒	☐ ChIP-seq
☒	☐ Flow cytometry
☒	☐ MRI-based neuroimaging

Unique biological materials

Policy information about availability of materials

Obtaining unique materials	The mutants in our study are unique biological material.

Antibodies

Antibodies used	Anti-SNC1 polyclonal antibody produced in rabbit was generated against a SNC1-specific peptide (RKTMTPSDDFGDC) at GenScript. Other antibodies used in immunoblotting experiments include anti-SUL45 (dilution, 1:1000)and anti-GAPDH (Santa Cruz Biotechnology, sc-365062) (dilution, 1:1000). The secondary antibodies were horseradish peroxidase-conjugated goat-anti-rabbit IgG (Bio-Rad, cat.#172-1019) (dilution, 1:2000) and goat-anti-mouse IgG (Bio-Rad, cat.#170-6516) (dilution, 1:2000).
Validation	We have validated these antibodies in this study.
